# MRE11-Deficiency Associated with Improved Long-Term Disease Free Survival and Overall Survival in a Subset of Stage III Colon Cancer Patients in Randomized CALGB 89803 Trial

**DOI:** 10.1371/journal.pone.0108483

**Published:** 2014-10-13

**Authors:** Thomas Pavelitz, Lindsay Renfro, Nathan R. Foster, Amber Caracol, Piri Welsch, Victoria Valinluck Lao, William B. Grady, Donna Niedzwiecki, Leonard B. Saltz, Monica M. Bertagnolli, Richard M. Goldberg, Peter S. Rabinovitch, Mary Emond, Raymond J. Monnat, Nancy Maizels

**Affiliations:** 1 Department of Immunology, University of Washington, Seattle, Washington, United States of America; 2 Division of Biomedical Statistics and Informatics, Mayo Clinic, Rochester, Minnesota, United States of America; 3 Molecular and Cellular Biology Graduate Program, University of Washington, Seattle, Washington, United States of America; 4 Department of Genome Sciences, University of Washington Medical School, Seattle, Washington, United States of America; 5 Clinical Research Division, Fred Hutchinson Cancer Research Center, Seattle, Washington, United States of America; 6 Department of Surgery, University of Washington Medical School, Seattle, Washington, United States of America; 7 Department of Medicine, University of Washington Medical School, Seattle, Washington, United States of America; 8 Cancer and Leukemia Group B Statistical Center, Duke University Medical Center, Durham, North Carolina, United States of America; 9 Memorial Sloan-Kettering Cancer Center, New York, New York, United States of America; 10 Dana-Farber Cancer Institute and Brigham and Women’s Hospital, Boston, Massachusetts, United States of America; 11 The Ohio State University, Columbus, Ohio, United States of America; 12 Department of Pathology, University of Washington Medical School, Seattle, Washington, United States of America; 13 Department of Biostatistics, University of Washington, Seattle, Washington, United States of America; 14 Department of Biochemistry, University of Washington, Seattle, Washington, United States of America; 15 Department of Chemistry, University of Washington, Seattle, Washington, United States of America; Institute of Experimental Endocrinology and Oncology G. Salvatore' (IEOS), Italy

## Abstract

**Purpose:**

Colon cancers deficient in mismatch repair (MMR) may exhibit diminished expression of the DNA repair gene, *MRE11*, as a consequence of contraction of a T_11_ mononucleotide tract. This study investigated MRE11 status and its association with prognosis, survival and drug response in patients with stage III colon cancer.

**Patients and Methods:**

Cancer and Leukemia Group B 89803 (Alliance) randomly assigned 1,264 patients with stage III colon cancer to postoperative weekly adjuvant bolus 5-fluorouracil/leucovorin (FU/LV) or irinotecan+FU/LV (IFL), with 8 year follow-up. Tumors from these patients were analyzed to determine stability of a T_11_ tract in the *MRE11* gene. The primary endpoint was overall survival (OS), and a secondary endpoint was disease-free survival (DFS). Non-proportional hazards were addressed using time-dependent covariates in Cox analyses.

**Results:**

Of 625 tumor cases examined, 70 (11.2%) exhibited contraction at the T_11_ tract in one or both *MRE11* alleles and were thus predicted to be deficient in MRE11 (dMRE11). In pooled treatment analyses, dMRE11 patients showed initially reduced DFS and OS but improved long-term DFS and OS compared with patients with an intact MRE11 T_11_ tract. In the subgroup of dMRE11 patients treated with IFL, an unexplained early increase in mortality but better long-term DFS than IFL-treated pMRE11 patients was observed.

**Conclusions:**

Analysis of this relatively small number of patients and events showed that the dMRE11 marker predicts better prognosis independent of treatment in the long-term. In subgroup analyses, dMRE11 patients treated with irinotecan exhibited unexplained short-term mortality. MRE11 status is readily assayed and may therefore prove to be a useful prognostic marker, provided that the results reported here for a relatively small number of patients can be generalized in independent analyses of larger numbers of samples.

**Trial Registration:**

ClinicalTrials.gov NCT00003835

## Introduction

Colorectal cancer (CRC) is the third most common cancer, and the second most common cause of cancer-related death in the US, after lung cancer [1]. There will be an estimated 143,000 new cases in the US in 2013, and more than 51,000 deaths due to this cancer. It is important to identify markers that report on disease prognosis.

Like many other types of cancer, CRC is characterized by deficiencies in DNA repair pathways that can affect evolution of the tumor, its response to chemotherapy, and survival in the short and long term [2]. Approximately 15% of sporadic CRC and most hereditary CRC are characterized by deficient mismatch repair (MMR-D), which is also common in other cancers, including endometrial and gastric tumors [3]–[5]. MMR-D CRC are recognized as a distinct pathological and clinical subclass, with better long-term prognosis but possibly limited response to standard adjuvant chemotherapy consisting of 5-fluorouracil (FU) and leucovorin (LV) [2], [6], [7].

Deficient MMR elevates the somatic mutation rate and destabilizes simple sequence repeats, or microsatellites, which in turn can affect gene sequence and gene functions [8]. Deficient MMR could affect prognosis directly or indirectly, by altering function of another gene or genes. One consequence of deficient MMR is contraction of a T_11_ tract in intron 4 of the *MRE11* gene, which is evident in over 60% of MMR-D CRC [9]–[11]. The T_11_ polypyrimidine tract promotes lariat formation in splicing of exon 4 to exon 5 of the MRE11 transcript. Contraction of that tract impairs splicing, resulting in exon skipping and synthesis of a mRNA carrying an out-of-frame stop codon. This mRNA encodes a truncated MRE11 polypeptide, with potentially dominant negative effect on function of the normal protein [12]. The status of the *MRE11* T_11_ tract can be readily determined by the standard clinical assay used to determine MMR status based on instability of neutral microsatellite markers [13].

MRE11-deficiency may affect both clonal evolution within a tumor as well as therapeutic response. MRE11 forms one component of the highly conserved MRE11/RAD50 complex, which is essential for DNA double-strand break repair mediated by both homologous recombination and non-homologous end-joining, for telomere maintenance, and for signaling in response to DNA damage [14]–[18]. MRE11 may in particular influence the response to topoisomerase 1 poisons, which function by trapping the normally transient covalent bond that topoisomerase 1 forms with DNA in order to relax supercoiling. This class of drugs includes the naturally occurring compound camptothecin, and its derivatives irinotecan and topotecan. MRE11 is highly conserved, and genetic analysis in the yeast, *S. cerevisiae*, has shown that MRE11-deficiency causes extreme sensitivity to camptothecin [19]. In vitro, purified recombinant MRE11/RAD50 can cleave the covalent tyrosyl-DNA bond formed by topoisomerase 1 and resect the DNA end for repair [20]. In addition, a limited study of five CRC cell lines found that those that were MRE11/RAD50-deficient were more sensitive to irinotecan [21], but did not determine whether irinotecan resistance could be restored by complementing the MRE11/RAD50-deficiency/.

The observations summarized above lead to two hypotheses. First, MRE11-deficiency might be a useful marker for tumor prognosis; and second, that MRE11-deficient (dMRE11) tumors might respond better to treatment with topoisomerase 1 poisons than MRE11-proficient (pMRE11) tumors. These possibilities were of particular interest because of the readiness with which MRE11 status can be assayed during standard clinical molecular profiling [13].

The utility of irinotecan has been assessed for adjuvant treatment of stage III CRC in a two arm Cancer and Leukemia Group B (CALGB) 89803 clinical trial, which compared DFS and OS in patients treated with FU/LV alone or in combination with irinotecan. No difference in OS or DFS was reported overall, but patients with MMR-D tumors exhibited somewhat improved and extended DFS if treatment included irinotecan [22]. This trial therefore provided informative samples for addressing the question of whether MRE11 status correlated with DFS, OS, or with response to irinotecan. In order to determine whether MRE11 status predicts DFS, OS, or response to IFL, we analyzed MRE11 status in 625 tumor samples from patients in the CALGB 89803 clinical trial, both overall and by treatment with FU/LV therapy with or without irinotecan, while accounting for potential relationships with other patient characteristics including MMR.

## Materials and Methods

The protocol for this trial and supporting CONSORT checklist are available as supporting information; see Protocol S1 and Checklist S1.

### Study population

**Figure 1:**
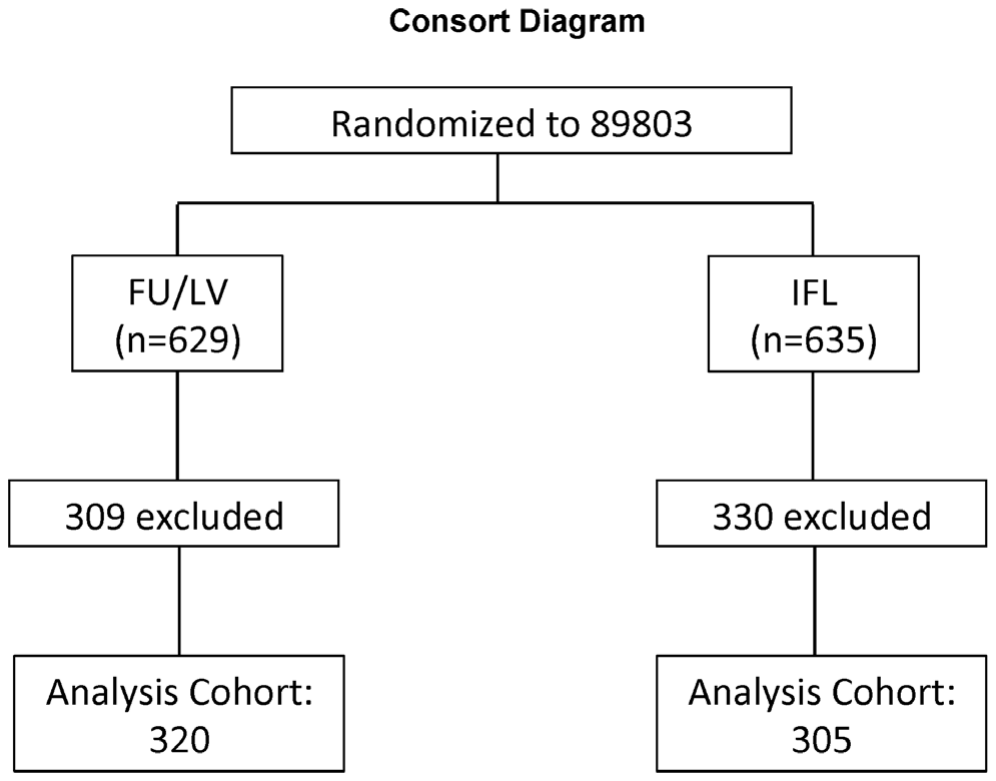
Consort diagram. Outline of CALGB 89803 randomized trial which generated the 625 samples tested.

Patients in this study were participants in the NCI-sponsored Cancer and Leukemia Group B (CALGB) adjuvant therapy trial for stage III colon cancer comparing therapy with the weekly Roswell Park regimen of 5-fluorouracil (FU) and leucovorin (FU/LV) with the weekly bolus regimen of irinotecan, FU, and leucovorin (CALGB 89803; [23]). A total of 1264 patients were recruited between April, 1999, and April, 2001. All patients underwent complete surgical resection and started chemotherapy between postoperative days 21 to 56. Patients were randomly assigned by computer to two treatment arms, 629 patients to FU plus LV and 635 patients to irinotecan plus FU plus LV (Figure 1). The primary study endpoint was overall survival (OS). Disease-free survival (DFS) was a secondary endpoint. Follow-up was captured as of March, 2008.

### Ethics statement

The study was approved by the Mayo Clinic Institutional Review Board and the North Central Cancer Treatment Group (now part of Alliance for Clinical Trials in Oncology). CALGB protocol 89803 was reviewed by the institutional review board of each participating center. All patients gave written informed consent before participation.

### Trial structure and organization

This trial was conducted by CALGB with participation by the North Central Cancer Treatment Group, National Cancer Institute of Canada Clinical Trials Group, Eastern Cooperative Oncology Group, Southwest Oncology Group, and the National Cancer Institute Cancer Trials Support Unit. The protocol and list of participating sites are available as Supporting Information. The CALGB data safety monitoring board reviewed safety data twice yearly and efficacy data at protocol-specified intervals in accordance with CALGB policies. The CALGB Statistical Center at Duke University in Durham, NC, maintained the clinical and laboratory database.

### Treatment

After central registration, eligible patients were randomly assigned (by computer, using a randomized fixed block design) to receive FU/LV or FU/LV in combination with irinotecan (IFL). Treatment has previously been described in detail [24]. In brief, the FU/LV group received the Roswell Park regimen, consisting of weekly LV 500 mg/m^2^ intravenously over 2 hours, with a bolus of FU 500 mg/m^2^ by intravenous injection 1 hour after initiation of LV, for 6 consecutive weeks followed by a 2-week rest, for four cycles (32 weeks). The IFL group received weekly irinotecan 125 mg/m^2^ over 90 minutes followed immediately by intravenous bolus injections of LV 20 mg/m^2^, then FU 500 mg/m^2^, for 4 consecutive weeks followed by a 2-week rest, for five cycles (30 weeks).

### DNA extraction

DNA was extracted from archived formalin-fixed paraffin-embedded tumor tissue by incubating the paraffin-extracted, rehydrated tissue in 50 mM Tris HCl (pH 8.5) with 0.5% Tween 20 and 20 mg/ml proteinase K for 3 hr at 55°C; or by incubating the tissue in Instagene (BioRad, Hercules, CA) and 30 mg/ml proteinase K for 3 hr at 55°C. After the incubation, the sample was then incubated at 95°C for 9 minutes, vortexed briefly, and then subjected to centrifugation to pellet any undigested material or the Instagene, respectively. The extracted DNA was then aliquoted and stored at −20°C until needed for the PCR based assays.

### Determination of *MRE11* and *RAD50* mononucleotide tract lengths

Mononucleotide tract length was determined by PCR amplification and DNA sequencing, using a well-established approach widely used to characterize the heterogeneity in mononucleotide tracts characteristic of MMR-deficient colorectal, gastric and endometrial tumors, and not evident in normal tissue or MMR-proficient tumor samples (e.g. [9]–[11], [25]). The assay involves PCR amplification of the region carrying mononucleotide tract, followed by DNA sequence analysis. This same simple procedure is used to assess microsatellite instability diagnostic of mismatch repair deficiency.

A total of 625 samples generated DNA suitable for analysis of the region of *MRE11* intron 4 containing the T_11_ tract, 320 from the FU/LV study arm and 305 from the IFL arm (Figure 1).

Nested PCR primers were used to amplify a region containing the mononucleotide tract of interest in MRE11 intron 4. MRE11 amplification was with first round primers, MRE11×1F, 5′-GTGGTCATATGCCAATGTAGATTATGC-3′, and MRE11×1R, 5′-CCCTGTGGGATCGTCATGATTGCC-3′, produced a 211 bp product; and with second round primers, MRE11×2F, 5′-GGAGGAGAATCTTAGGGAAAACAGC-3′, and MRE11×2R, 5′-GATTGCCATGAATACTAAACACTGG-3′, produced a 139 bp product. MRE11 was sequenced in both forward and reverse directions with the second round PCR primers.

RAD50 status was determined for 34 CRCs with contractions in *MRE11*. Amplification was with first round primers R50×1F, 5′-CTCCCAGTTCATTACTCAGC-3′, and R50×1R, 5′-GACAGGGCATACCAGCT-3′, produced a 326 bp product; and with second round primers R50×2F, 5′-GCTAACAGACGAAAACCAG-3′, and R50×2R, 5′-CATACCAGCTCAGAGTCC-3′, produced a 301 bp product. RAD50 was sequenced in reverse orientation with primer 5′-CATACCAGCTCAGAGTCC-3′.

MRE11 status and RAD50 status were determined by visual inspection of tracings from automated sequencing in both the forward and reverse direction by investigators blinded to MMR status, which had been determined independently [22], [26]. Results are presented in Tables 1 and 2.

**Table 1:** Demographics of Study Population by MRE11 Status.

	Ineligible MRE11 Analyses(N = 639)	Eligible MRE11 Analyses(N = 625)	Total(N = 1264)	p value
**Treatment arm**				0.3121^1^
5FU/LV	309 (48.4%)	320 (51.2%)	629 (49.8%)	
CPT-11/5FU/LV	330 (51.6%)	305 (48.8%)	635 (50.2%)	
**Age at study entry**				**0.0144** 2
Mean (SD)	59.2 (11.5)	60.5 (11.4)	59.9 (11.5)	
Median	59.0	63.0	61.0	
Range	(21.0–85.0)	(24.0–85.0)	(21.0–85.0)	
**Gender**				0.6415^1^
Male	359 (56.2%)	343 (54.9%)	702 (55.5%)	
Female	280 (43.8%)	282 (45.1%)	562 (44.5%)	
**Tumor Site**				0.5030^1^
Missing	17	11	28	
Distal	268 (43.1%)	253 (41.2%)	521 (42.2%)	
Proximal	354 (56.9%)	361 (58.8%)	715 (57.8%)	
**Performance status**				0.4169^1^
Missing	18	9	27	
0	458 (73.8%)	467 (75.8%)	925 (74.8%)	
1	158 (25.4%)	147 (23.9%)	305 (24.7%)	
2	5 (0.8%)	2 (0.3%)	7 (0.6%)	
**Positive Nodes**				0.1703^2^
N	624	616	1240	
Mean (SD)	3.4 (3.3)	3.7 (3.6)	3.6 (3.4)	
Median	2.0	3.0	2.0	
Range	(0.0–29.0)	(1.0–24.0)	(0.0–29.0)	
**Histologic Grade**				0.2419^1^
Missing	17	10	27	
Grade 1/2	478 (76.8%)	455 (74.0%)	933 (75.4%)	
Grade 3/4	144 (23.2%)	160 (26.0%)	304 (24.6%)	
**T-Stage**				0.7941^1^
Missing	18	13	31	
T12	83 (13.4%)	74 (12.1%)	157 (12.7%)	
T3	486 (78.3%)	487 (79.6%)	973 (78.9%)	
T4	52 (8.4%)	51 (8.3%)	103 (8.4%)	
**MMR Status**				0.7890^1^
Missing	310	44	354	
MMR-I	283 (86.0%)	496 (85.4%)	779 (85.6%)	
MMR-D	46 (14.0%)	85 (14.6%)	131 (14.4%)	
**BRAF600**				0.8919^1^
Missing	567	40	607	
Wild-Type	61 (84.7%)	492 (84.1%)	553 (84.2%)	
Mutant	11 (15.3%)	93 (15.9%)	104 (15.8%)	
**KRAS**				0.8518^1^
Missing	569	41	610	
Wild-Type	45 (64.3%)	382 (65.4%)	427 (65.3%)	
Mutant	25 (35.7%)	202 (34.6%)	227 (34.7%)	
**P53**				0.2703^1^
Missing	439	216	655	
Wild-Type	103 (51.5%)	230 (56.2%)	333 (54.7%)	
Mutant	97 (48.5%)	179 (43.8%)	276 (45.3%)	

1 chi-squared tests, for difference between MRE11-eligible and MRE11-ineligible patients.

according to relevant factors.

2 Wilcoxon test for difference between MRE11-eligible and MRE11-ineligible patients according to relevant factors.

**Table 2:** Demographics by MRE11 Status (dMRE11 vs. pMRE11).

	dMRE11 (N = 70)	pMRE11 (N = 555)	Total (N = 625)	p value
**Treatment arm**				0.2194^1^
5FU/LV	31 (44.3%)	289 (52.1%)	320 (51.2%)	
CPT-11/5FU/LV	39 (55.7%)	266 (47.9%)	305 (48.8%)	
**Age at study entry**				0.2133^2^
Mean (SD)	60.8 (14.0)	60.5 (11.1)	60.5 (11.4)	
Median	66.0	62.0	63.0	
Range	(24.0–81.0)	(24.0–85.0)	(24.0–85.0)	
**Gender**				0.2603^1^
Male	34 (48.6%)	309 (55.7%)	343 (54.9%)	
Female	36 (51.4%)	246 (44.3%)	282 (45.1%)	
**Tumor site**				**<0.0001** 1
Missing	2	9	11	
Distal	10 (14.7%)	243 (44.5%)	253 (41.2%)	
Proximal	58 (85.3%)	303 (55.5%)	361 (58.8%)	
**Performance status**				0.8624^1^
Missing	2	7	9	
0	51 (75.0%)	416 (75.9%)	467 (75.8%)	
1	17 (25.0%)	130 (23.7%)	147 (23.9%)	
2	0 (0.0%)	2 (0.4%)	2 (0.3%)	
**Positive nodes**				0.4688^2^
N	68	548	616	
Mean (SD)	4.2 (4.1)	3.7 (3.5)	3.7 (3.6)	
Median	3.0	3.0	3.0	
Range	(1.0–22.0)	(1.0–24.0)	(1.0–24.0)	
**Histologic Grade**				**0.0001** 1
Missing	2	8	10	
Grade 1/2	37 (54.4%)	418 (76.4%)	455 (74.0%)	
Grade 3/4	31 (45.6%)	129 (23.6%)	160 (26.0%)	
**T-Stage**				0.1056^1^
Missing	2	11	13	
T12	6 (8.8%)	68 (12.5%)	74 (12.1%)	
T3	52 (76.5%)	435 (80.0%)	487 (79.6%)	
T4	10 (14.7%)	41 (7.5%)	51 (8.3%)	
**MMR Status**				**<0.0001** 1
Missing	3	41	44	
MMR-I	14 (20.9%)	482 (93.8%)	496 (85.4%)	
MMR-D	53 (79.1%)	32 (6.2%)	85 (14.6%)	
**BRAF600**				**<0.0001** 1
Missing	4	36	40	
Wild-Type	33 (50.0%)	459 (88.4%)	492 (84.1%)	
Mutant	33 (50.0%)	60 (11.6%)	93 (15.9%)	
**KRAS**				**<0.0001** 1
Missing	3	38	41	
Wild-Type	59 (88.1%)	323 (62.5%)	382 (65.4%)	
Mutant	8 (11.9%)	194 (37.5%)	202 (34.6%)	
**P53**				**0.0300** 1
Missing	22	194	216	
Wild-Type	34 (70.8%)	196 (54.3%)	230 (56.2%)	
Mutant	14 (29.2%)	165 (45.7%)	179 (43.8%)	
**RAD50**				0.8062^1^
Missing	36	544	580	
Wild-Type	23 (67.6%)	7 (63.6%)	30 (66.7%)	
Mutant	11 (32.4%)	4 (36.4%)	15 (33.3%)	

1 chi-squared test.

2 Wilcoxon Rank-Sum test.

### Determination of MMR status

MMR status of tumor samples had been previously determined by IHC, supplemented in some cases by analysis of microsatellite stability using the Bethesda panel markers [22], [26]. MMR status of a subset of samples was independently confirmed in a blinded analysis by PCR amplification and sequencing 5 microsatellite markers (Promega, Madison, WI). Samples were classified as MMR-D if 3 or more markers exhibited instability.

### Statistical methods

The goal of this study was to determine whether tumor MRE11 status was associated with outcome for patients with stage III colon cancer treated either with FU/LV alone or in combination with irinotecan. The joint primary endpoints were OS, measured from entry onto the clinical trial until death from any cause; and DFS, measured from study entry until documented progression of disease or death from any cause. OS and DFS distributions were estimated overall and within categories defined by MRE11 and treatment, using Kaplan-Meier methodology. Differences in OS and DFS between groups were tested using the log-rank test. The effects of MRE11 on OS and DFS were analyzed using Cox proportional hazards models. Where the Cox proportional hazards assumption was significantly violated according to the method of Grambsch and Therneau [27], time-varying coefficients were introduced through automated selection of one or more cutpoints on the time axis, which in turn optimally satisfied the proportional hazards assumption in a piecewise fashion [28]. The potential predictive ability of MRE11 was explored through two-way interactions with treatment arm, and through partial interactions when the effect of MRE11 was modeled as time-dependent. Interactions that were both statistically significant (p<0.01) and of interpretable clinical relevance were required to conclude meaningful predictive ability of MRE11. Multivariable Cox models were used to study the MRE11 effect while controlling for treatment and clinicopathologic factors including age, sex, tumor location, performance status, number of positive lymph nodes, tumor stage, and tumor grade. Potential MRE11 interactions with and adjustments by MMR, KRAS, and BRAF were also explored. For the purposes of this analysis, follow-up was limited to 8 years. All statistical analyses were performed by Alliance statisticians.

## Results

### Determination of MRE11 status

**Figure 2:**
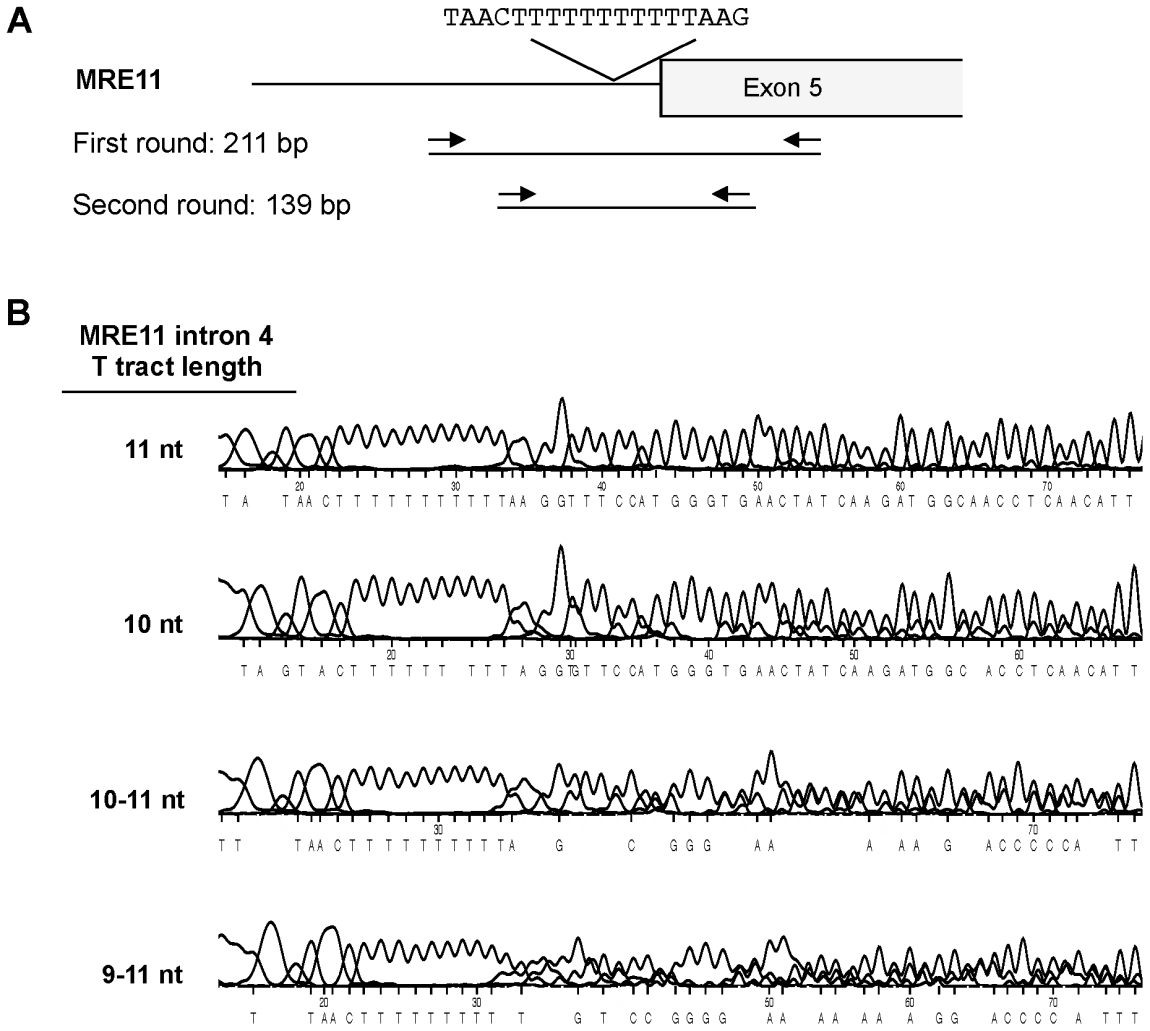
Genomic PCR assay of *MRE11* intron 4 T_11_ tract. (A) Diagram of the *MRE11* intron 4/exon 5 junction, showing the T_11_ tract in intron 4, flanking sequence and primers. Contraction of the T_11_ tract impairs the lariat formation step in mRNA splicing and leads to skipping of exon 5. The resulting mutant mRNA encodes a truncated MRE11 polypeptide with potentially dominant negative effect on protein function [12]. MRE11 is essential for cell viability, and the *MRE11* mutations that occur in MMR-D CRC are not null alleles but reduce expression and activity of the MRE11 protein. (B) Sequence traces of the region of *MRE11* intron 4 that carries the T_11_ tract in four tumor samples. Lengths of tracts in nt shown at left.

Tumor DNA suitable for PCR amplification was extracted from a total of 625 CRC specimens, 320 from the FU/LV group and 305 from the ILV group (Consort Diagram, Figure 1). The 625 specimens represent 49% of the 1264 patients enrolled on CALGB 89803 [23]. The region of *MRE11* intron 4 containing the T_11_ tract was amplified and sequenced (Figure 2A), using a well-established assay (e.g. [9]–[11], [25]). Examples show sequences of a control sample with uniform T_11_ tracts on both alleles, and of samples in which both alleles carried 10 nt tracts, or tracts ranging from 10–11 or 9–11 nt (Figure 2B). Tract length heterogeneity was not a PCR or sequencing artefact, as length heterogeneity was not evident in DNA from normal cells, and identical tract lengths were deduced from sequencing a single sample in both directions (not shown). Heterogeneity could reflect the presence of multiple sub-clonal populations within the tumor.

Cases were scored based on the number of nucleotides lost by contraction: an 11 nt tract was scored as 0; a 10–11 nt tract as 1; tracts of 9–11 nt as 2; and tracts of 10 nt (due to loss of 1 nt on each allele) as 2. A total of 555 cases (89%) carried an intact T_11_ tract (score 0); while 70 cases (11%) had contractions of 1–6 nt in length (1 nt, n = 36; 2 nt, n = 26; 3 nt, n = 6; 4 nt, n = 1; and 6 nt, n = 1). The overall frequency of *MRE11* T_11_ contractions was 11%, comparable that reported in other analyses of CRC specimens [9]–[11], [25]. Approximately equal numbers of cases exhibited contractions of 1 nt (36 cases, 5.8%) or 2 or more nt (34 cases, 5.8%). Due to an imbalance of cases with no contractions versus any contraction, and the small number of cases in each contraction category, we defined a dichotomous *MRE11* T_11_ tract variable: no contractions or MRE11 proficient (pMRE11, 555 cases) versus any contraction or MRE11 deficient (dMRE11, 70 cases). Using this dichotomized classification, *MRE11* T_11_ tract contraction status was found to be significantly associated with tumor site, histological grade, MMR status, and BRAF, KRAS, and P53 mutation status (Table 2).

### Determination of RAD50 status

In some MMR-D CRC, an exonic A_9_ tract in the *RAD50* gene is destabilized, causing a frameshift mutation and synthesis of a truncated protein [29]. In an exploratory analysis, we determined this frequency among tumor DNAs extracted from 34 CRCs with contractions in *MRE11* (18 cases with MRE11 scores of 1, and 16 with scores of 2). The *RAD50* A_9_ tract was unstable in 11/34 cases (3 with MRE11 scores of 1, and 8 with scores of 2) or 32% of CRCs analyzed, similar to the frequency of 40% previously reported [11].

### Determination of MMR status

MMR status of all CALGB 89803 samples had previously been determined by IHC, genomic sequencing with the Bethesda panel markers, or both [26]. That analysis classified MMR as intact (MMR-I) in 86% of the samples, and deficient (MMR-D) in 14% (Table 1). Instability of the *MRE11* T_11_ tract was strongly associated with MMR deficiency thus determined: 79% of dMRE11 CRCs were MMR-D, and 21% MMR-I (chi-squared test, p<0.0001; Table 2).

In an exploratory analysis, MMR status was separately determined by PCR and sequencing at the University of Washington (UW) clinical diagnostic facility, exclusively using commercial primers (Promega) that interrogate different neutral and non-polymorphic markers than the Bethesda panel markers. A total of 83 CRCs were analyzed, including 63 of the 70 dMRE11 samples. Of these, 50 CRC were classified as MMR-D by CALGB and 56 by the UW (chi-squared test, p<0.0001).

Instability at the *MRE11* T_11_ tract and the *RAD50* A_9_ tract is predicted to correlate with MMR-D. To get a sense of whether the different assays used by CALGB and UW might over-count or under-count MMR-D tumors, we asked if the 9 CRC classified as MMR-I by the CALGB assays but as MMR-D by the UW assay were dMRE11 or pMRE11. All of these were dMRE11, and two of them were also dRAD50. This raises the possibility that the *MRE11* T_11_ tract might be usefully included among markers for determination of MMR status.

### Tumor MRE11 status is significantly prognostic for DFS and OS

**Figure 3:**
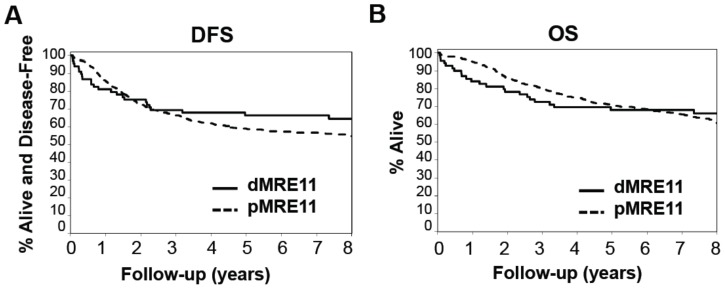
Tumor MRE11 status is significantly prognostic for DFS and OS. (A) Disease free survival for dMRE11 (n = 70; events = 24); 5-yr rate: 67% (95% CI: 56–79%) vs. pMRE11 (n = 555; events = 240); 5-yr rate: 59% (95% CI: 55–63%). (B) Overall survival for dMRE11 (n =  = 70; events = 23); 5-yr rate: 68% (95% CI: 58–80%) vs. pMRE11 (n = 555; events = 194); 5-yr rate: 71% (95% CI: 67–75%).

The relationship between MRE11 status and DFS and OS was determined using data captured as of March 10, 2008, representing a median follow-up of >6.0 years. Univariate analyses of OS and DFS based solely on MRE11 status of the 625 CRCs assayed, independent of chemotherapeutic regimen, showed that dMRE11 patients exhibited no significant improvement in OS (HR 0.98; 95% CI, 0.64 to 1.51) or DFS (HR 0.80; 95% CI, 0.52 to 1.21) relative to pMRE11 patients. However, Kaplan-Meier plots of OS and DFS by MRE11 status (Figure 3) revealed a possible violation of the proportional hazards assumption, with the two curves crossing during the follow-up period for each endpoint. Non-proportionality was statistically confirmed, with the null hypothesis of proportional hazards rejected for both DFS (p = 0.0038) and OS (p = 0.0005).

To resolve this violation of the Cox modeling assumptions, a piecewise proportional hazard model was constructed for each endpoint (DFS and OS), resulting in time dependent coefficients (HRs) for MRE11 status, as follows. First, an automated searching algorithm over a grid of time points {t_1_ = 0.1 years, t_2_ = 0.2 years, …, t_79_ = 7.9 years} was used to identify the cutpoint t* for which the proportional hazards assumption was optimally satisfied on either side of the cutpoint; specifically, t* is defined as the value of t yielding the largest maximized log partial likelihood among Cox models containing separate MRE11 effects (HRs) for the two time intervals defined by t. Piecewise proportionality was then tested and confirmed in the final models for OS and DFS, producing the final (univariate) models for MRE11. The same cutpoints and time-dependent coefficients were used in subsequent interaction and multivariable Cox models.

The optimal cut-point identified for OS was 3.4 years. Prior to 3.4 years, dMRE11 patients experience significantly worse OS relative to pMRE11 patients (HR = 10.95, 95% CI: 6.83 to 17.55, p<0.0001), while after 3.4 years dMRE11 is associated with improved OS (HR = 0.09, 95% CI: 0.02 to 0.37, p = 0.0008), independent of treatment arm. The cut-point identified for DFS was 3.3 years. Prior to 3.3 years, dMRE11 was associated with worse outcomes (HR = 7.02, 95% CI: 4.49 to 10.99, p<0.0001), while after 3.3 years DFS is improved relative to pMRE11 patients (HR = 0.07, 95% CI: 0.02 to 0.30, p = 0.0002). MRE11 status remained significant overall when adjusted for clinical/tumor variables (age, sex, number of positive nodes, tumor stage, grade, and site of primary tumor).

In relation to other patient biomarkers, MRE11 is jointly significant in multivariable models with KRAS (OS and DFS), BRAF (OS and DFS), and P53 mutation status (OS only). Furthermore, MRE11 remains a significant predictor for both OS (p<0.0001) and DFS (p<0.0001) after adjustment for MMR, while MMR is not significant in these models (DFS p = 0.799; OS p = 0.647). No significant interactions between MRE11 and biomarkers were observed.

Univariate analyses for RAD50 status showed no significant relationship with OS or DFS. Covariate-adjusted models for RAD50 were not performed due to the limited sample size (n = 34) and small number of events.

### Assessment of tumor MRE11 status as predictive of benefit from IFL and FU/LV

**Figure 4:**
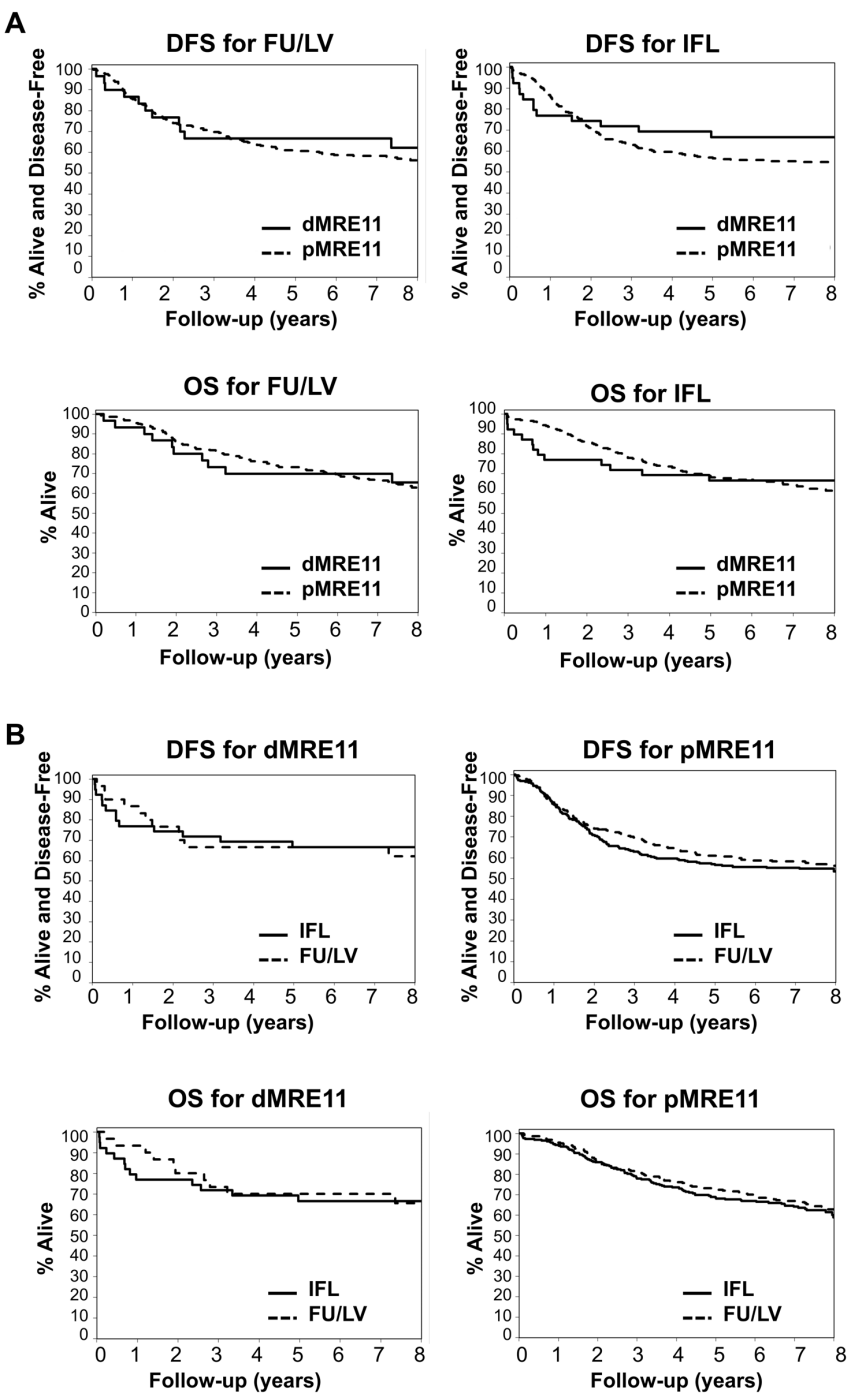
Assessment of tumor MRE11 status as predictive of benefit from FU/LV and IFL. (A) Top: Disease free survival for dMRE11 vs. pMRE11 treated with FU/LV [n = 320; dMRE11 n = 31; events = 11; 5-yr rate: 67% (95% CI: 52–86%); pMRE11 n = 289; events = 122; 5-yr rate: 61% (95% CI: 56–67%)] or with IFL [n = 305; dMRE11 n = 39; events = 13; 5-yr rate: 67% (95% CI: 53–83%); pMRE11 n = 266; events = 118; 5-yr rate: 57% (95% CI: 51–63%)]. Bottom: Overall survival for dMRE11 vs. pMRE11, treated with FU/LV [n = 320; dMRE11 n = 31; events = 10; 5-yr rate: 70% (95% CI: 55–89%); pMRE11 n = 289; events = 98); 5-yr rate: 73% (95% CI: 68–79%)] or with IFL [n = 305; dMRE11 n = 39; events = 13; 5-yr rate: 67% (95% CI: 53–83%); pMRE11 n = 266; events = 96; 5-yr rate: 69% (95% CI: 63–75%)]. (B) Top: Disease-free survival for IFL vs. FU/LV treated dMRE11 [n = 70; IFL N = 39; events = 13; 5-yr rate: 67% (95% CI: 53–83%); FU/LV n = 31; events = 11; 5-yr rate: 67% (95% CI: 52–86%)] or pMRE11 (n = 555; IFL n = 266; events = 118; 5-yr rate: 57% (95% CI: 51–63%; FU/LV n = 289; events = 122; 5-yr rate: 61% (95% CI: 56–67%)]. Bottom: Overall survival for IFL vs. FU/LV-treated dMRE11 [n = 70; IFL n = 39; events = 13; 5-yr rate: 67% (95% CI: 53–83%); FU/LV n = 31; events = 10; 5-yr rate: 70% (95% CI: 55–89%) or pMRE11 [(n = 555; IFL n = 266; events = 96; 5-yr rate: 69% (95% CI: 63–75%); FU/LV n = 289; events = 98; 5-yr rate: 73% (95% CI: 68–79%)].

MRE11 status was assessed as a potential predictor benefit from IFL through partial interactions with treatment in piecewise Cox (non-proportional hazards) models for DFS and OS. Kaplan-Meier plots for the MRE11 effect are presented by treatment arm in Figure 4A, while plots for the corresponding treatment effect are presented by MRE11 status in Figure 4B. While some differences in the treatment effect by MRE11 status are visually apparent, the treatment-by-MRE11 interaction was not significant for either endpoint. Among irinotecan-treated patients (Figure 4A, right), however, dMRE11 patients exhibited worse DFS than pMRE11 patients during the first year of follow-up (based on a subset-selected cut-point; p<0.0001), but improved outcomes thereafter (p = 0.004). Similarly, dMRE11 patients were at increased risk of death for the first 3.5 years of follow-up relative to pMRE11 patients (p<0.0001), and decreased risk thereafter (p = 0.011). These relationships remained significant when adjusted for age, sex, nodal status, tumor stage, grade, and site of primary tumor, but this subgroup analysis among IFL-treated patients should be considered as exploratory given the small sample size and non-significance of the treatment-by-MRE11 interaction.

## Discussion

This analysis of treated outcomes in a cohort of stage III CRC patients treated with FU/LV or IFL showed that, after controlling for unexpected non-proportional hazards, MRE11 status is significantly prognostic for both DFS and OS, and remains significant when adjusted for clinicopathologic variables and published significant markers such as MMR, KRAS, BRAF, and p53. Furthermore, after adjusting for MMR, MRE11 remains a significant prognostic marker, while the converse is not true. In an exploratory subgroup analysis, MRE11 status was associated with differences in OS and DFS among patients treated with IFL. The latter finding is clinically interesting, but based on a relatively small number of patients, and could be further investigated in studies enrolling larger numbers of patients. The impact on response might best be assessed in a study of stage IV patients with measurable metastatic disease, although it has been shown on many occasions that the effects of anti-tumor therapies differ between stage III and stage IV disease.

MMR status had previously been shown to be both a prognostic marker [2], [6], [7] and predictor of response to IFL relative to FU/LV [22]. In light of the strong correlation between dMRE11 and MMR-D status noted here, it is not surprising that the prognostic and predictive patterns for the two markers parallel one another. However, in multivariable prognostic models for DFS or OS containing both markers, MRE11 but not MMR status remained highly statistically significant during both early and late time periods. Thus, the significance of MRE11 status does not reflect its dependence on MMR status.

It should be noted that dMRE11 patients treated with IFL exhibited better long-term DFS than pMRE11 patients in the same treatment arm, although dMRE11 patients had an unexplained increased mortality in the first 2 years post-treatment. There was no relationship between poor initial response and clinical factors such as age, sex or nodal status. Therapy with irinotecan extended over only 30 weeks, while the difference in response did not become evident until later (Figure 4), so early treatment-associated toxicity alone is unlikely to explain this difference.

The analysis of MRE11 function in the response to irinotecan was undertaken based on results of basic mechanistic studies showing that MRE11/RAD50 contributes to repair of DNA damage induced by topoisomerase 1 poisons [19], [20]. Topoisomerase 1 poisons (like irinotecan) and topoisomerase 2 poisons (like etoposide) both promote formation of protein-DNA adducts, which are cytotoxic if not repaired. Some insight into the considerable short-term mortality among dMRE11 patients treated with irinotecan may be provided by studies of mice deficient in the enzyme TDP2, which repairs damage induced by topoisomerase 2 poisons [30]. These mice are hypersensitive to etoposide, and respond to treatment by dramatic weight loss accompanied by villous atrophy in the small intestine and lymphoid toxicity [31]. Analogously, dMRE11 tumor cells may be hypersensitive to irinotecan, but at the single dose tested the cytotoxic response may create a local milieu conducive to tumor cell proliferation, for example by promoting expression of growth factors or by limiting the immune response to the tumor during the early stages of tumor development. If so, drug hypersensitivity could cause poor outcomes in early but not in later years post-treatment.

*MRE11* T_11_ tract instability can be measured using an easy and reliable standard clinical assay like that already used to interrogate microsatellite instability. Analysis of the relatively small number of patients and events reported here showed that the dMRE11 marker predicts better prognosis independent of treatment in the long-term, suggesting that MRE11 may be a useful prognostic marker. Generalizability will require that these findings can be independently validated in another dataset that examines larger numbers of patients.

Deficiency in MRE11 occurs not only in colon cancer but also other solid tumors with deficient mismatch repair. Analysis of independent relevant datasets will be necessary to establish whether the results reported here extend to these other cancers.

## Supporting Information

Protocol S1 **Cancer and Leukemia Group B, CALBG 89803.** Protocol for phase III intergroup trial of irinotecan (CPT-11) plus fluorouracil/leucovorin (5-FU/LV) versus fluorouracil/leucovorin alone after curative resection for patients with stage III colon cancer.
(PDF)

Checklist S1 **CONSORT 2010 checklist.** Checklist of information to include when reporting a randomised trial.
(PDF)

Institutions S1 **CALBG 89803 Institutions.** Institutions and investigators that participated in the initial CALGB 89803 study.
(PDF)
