# Supplementary material for: MRE11-Deficiency Associated with Improved Long-Term Disease Free Survival and Overall Survival in a Subset of Stage III Colon Cancer Patients in Randomized CALGB 89803 Trial
Source: PLoS One. 2014 Oct 13;9(10):e108483. doi: 10.1371/journal.pone.0108483 (PMC4195600; doi:10.1371/journal.pone.0108483)
Supplement: Protocol S1 — Cancer and Leukemia Group B, CALBG 89803. Protocol for phase III intergroup trial of irinotecan (CPT-11) plus fluorouracil/leucovorin (5-FU/LV) versus fluorouracil/leucovorin alone after curative resection for patients with stage III colon cancer. (PDF) [file pone.0108483.s001.pdf]

Activation Date: 04/15/99
Includes Update #6

CANCER AND LEUKEMIA GROUP B

CALGB 89803
NCCTG C89803/NCIC CTG CO.15/EPP C89803/ECOG 89803/SWOG C89803/ CTSU C89803

PHASE III INTERGROUP TRIAL OF IRINOTECAN (CPT-11) (NSC#616348) PLUS FLUOROURACIL/LEUCOVORIN (5-FU/LV) VERSUS FLUOROURACIL/LEUCOVORIN ALONE AFTER CURATIVE RESECTION FOR PATIENTS WITH STAGE III COLON CANCER

Study Chair
Leonard B. Saltz, M.D.
Memorial Sloan-Kettering Cancer Center
1275 York Avenue
New York, NY 10021
Tel: 212-639-2501 Fax: 212-794-7186
saltzl@mskcc.org

CALGB Correlative Sciences Co-Chair
Robert S. Warren, M.D.
Tel: 415-476-4089
Fax: 415-476-8696
warrenr@surgery.ucsf.edu

CALGB Cancer Control Co-Chair
Charles Fuchs, M.D.
Tel: 617-632-2225
Fax: 617-632-3161
charles_fuchs@dfci.harvard.edu

GI Committee Chair
Richard Goldberg, M.D.
Tel: 919-966-5720
Fax: 919-966-4131
goldberg@med.unc.edu

Pathology Committee Chair
Carolyn Compton, M.D., Ph.D.
Tel: 617-726-6875
Fax: 617-720-0215
carolyn.compton@mcgill.ca

GI Correlative Sciences Working Group Chair
Monica M. Bertagnolli, M.D.
Tel: 212-746-2195
Fax: 212-746-8765
mbertagn@mail.med.cornell.edu

Cancer Control Committee Chair
Electra Paskett, Ph.D.
Tel: 336-716-6946
Fax: 336-716-5425
epaskett@rc.phs.wfubmc.edu

NCCTG Study Coordinator
Frank A. Sinicrope, M.D
Mayo Clinic
Tel: 507-266-5635

EPP Contact
Gity Nasim
Tel: 301-299-8655
Fax: 301-299-3991
gnasim@emmes.com

NCIC-CTG Study Coordinator
David Klaassen, M.D.
BCCA-Vancouver Cancer Centre
goldberg.richard@mayo.edu

SWOG Study Coordinator
Alexander Hantel, M.D.
Tel: 630-527-3788
Fax: 630-527-3790
ahantel@luc.edu

CTSU Study Coordinator
Anita Nelson
Tel: 301-610-5581
Fax: 301-738-8379
nelsona@westat.com

ECOG Study Coordinator
James P. Thomas, M.D., Ph.D.
Tel: 608-265-8756
Fax:608-263-8613
jpt@medicine.wisc.edu

Faculty Statistician
Donna Niedzwiecki, Ph.D.
Tel: 919-681-5030 Fax: 919-681-8028
dniedzwiecki@ccstat.mc.duke.edu

Staff Statistician
Donna R. Hollis,M.S.
Tel: 919-681-5027 Fax: 919-681-8028
dhollis@ccstat.mc.duke.edu

Data Coordinator
Gwyndolyn Carver
Tel: 919-668-9391 Fax: 919-668-9348
g.carver@duke.edu

Protocol Editor
John R. Taylor, MA
Tel: 773-702-1767 Fax: 312-345-0117
jtaylor1@uchicago.edu

CALGB Central Office
230 West Monroe Street, Suite 2050
Chicago, IL 60606
(773) 702-9171
Fax: (312) 345-0117

ADR Reporting:
CALGB Central Office: (773) 702-9860
NCI - Investigative Drug Branch: (301) 230-2330

CALGB Statistical Center
Hoch Plaza
2424 Erwin Road, Suite 802
Durham, NC 27705
(919) 668-9350
Data Operations Fax: (919) 668-9348
Biostatistics Fax: (919) 681-8028

CALGB Patient Registration: (919) 668-9396

NCCTG Patient Registration:
Fax: (507) 284-0085

NCIC CTG Study Contact
Cathy Sears
Intergroup Trials Assistant
Tel: 613-533-6430

EPP Patient Registration: See Appendix V

CALGB Pathology Coordinating Office
The Ohio State University
320 West 10th Ave.
M364 Starling-Loving Hall
Columbus, OH 43210
Tel: (614) 293-7073
Fax: (614) 293-7967

Eastern Cooperative Oncology Group

ECOG Study Coordinator
James P. Thomas, M.D., Ph.D.
UW Comprehensive Cancer Center K4/638
Clinical Science Center
600 Highland Ave
Madison, WI 53792
Tel: 608-265-8756
Fax:608-263-8613
jpt@medicine.wisc.edu

ECOG Patient Registration:
(Tel) 617-632-2022

Southwest Oncology Group

SWOG Study Coordinator
Alexander Hantel, M.D.
Loyola University/Edward Hospital

120 Spalding Drive, Suite 400
Naperville, IL 60540
Tel: 630-527-3788
Fax: 630-527-3790
ahantel@luc.edu

SWOG Patient Registration:
(Tel) 206 667-4623

North Central Cancer Treatment Group

Medical Oncology Co-Chair
Richard M. Goldberg, M.D.
Mayo Clinic
200 First Street SW
Rochester, MN 55905
Tel: 507-266-0029 Fax: 507-284-1803
goldberg.richard@mayo.edu

Surgery Co-Chair

Heidi Nelson, M.D.
Mayo Clinic
Tel: 507-284-2511

NCCTG Patient Registration:
(Fax) 507-284-0885

National Cancer Institute of Canada – Clinical Trials Group

Study Coordinator
David Klaassen, M.D.
Division of Medical Oncology
BCCA-Vancouver Cancer Centre
600 West 10th Ave.
Vancouver, BC
Canada V5Z 4E6

NCIC-CTG Patient Registration:
Tel: 613-533-6430

Extended Participation Project

EPP Contact
Gity Nasim
Tel: 301-299-8655
Fax: 301-299-3991
gnasim@emmes.com

EPP Patient Registration:
See Appendix VII

Clinical Trials Support Unit

CTSU Contact
Anita Nelson
Tel: 301-610-5581 Fax: 301-738-8379
nelsona@westat.com

CTSU Website
www.ctsu.org

CTSU General information
Tel: 1-888-823-5923
ctsucontact@westat.com

CTSU Patient Registration and Adverse Event Reporting
Tel:1-888-462-3009 Fax: 1-888-691-8039

PHASE III INTERGROUP TRIAL OF IRINOTECAN (CPT-11) (NSC#616348) PLUS FLUOROURACIL/LEUCOVORIN (5-FU/LV) VERSUS FLUOROURACIL/LEUCOVORIN ALONE AFTER CURATIVE RESECTION FOR PATIENTS WITH STAGE III COLON CANCER

Patient Eligibility

Histologically documented adenocarcinoma of the colon.
The gross inferior (caudad) margin of the primary tumor must lie above the peritoneal reflection.
Completely resected tumor, with pathologically documented Modified Astler-Coller Stage C (T_x,N_{1-2},M_0) see Sec. 5.1.
No history of distant metastatic disease.
No prior chemotherapy or radiotherapy for this malignancy.
No previous or concurrent malignancy except as specified in Section 5.2.2.
Age ≥ 18 years.
Zubrod Performance status: 0-2
Non-pregnant, non-lactating.

Required Initial Laboratory Values

Granulocytes	≥ 1,500/µl
Platelet Count	≥ 100,000/µl
Creatinine	≤ 1.5 x Upper Limit of Normal (ULN)
Bilirubin	≤ ULN

SCHEMA

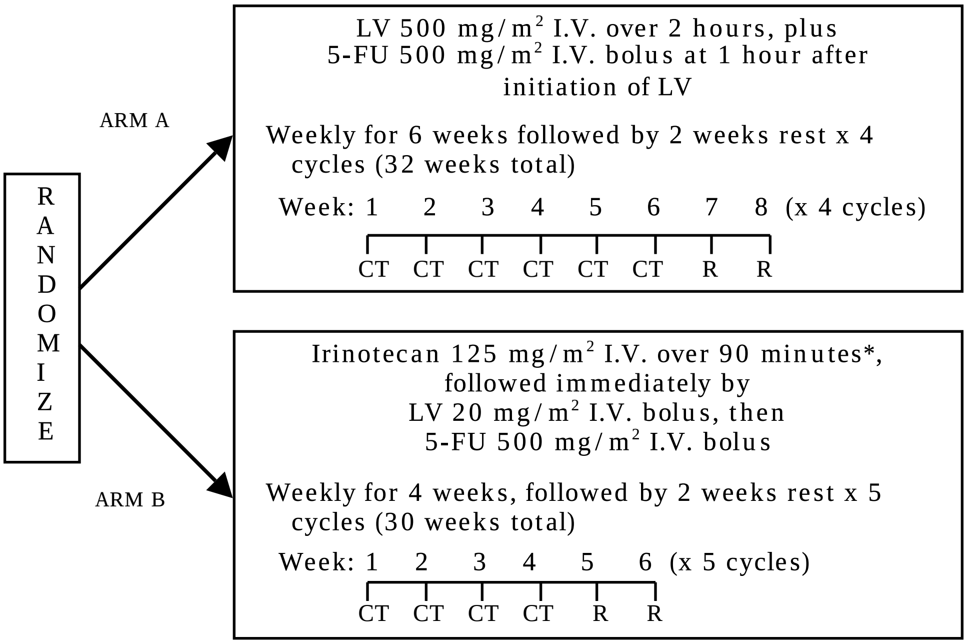

CT = chemotherapy; R = Rest

* For the first two cycles of irinotecan, patients should remain in the treatment area for a minimum of one hour following completion of the irinotecan infusion in case acute abdominal cramping develops.

Patients must begin first treatment no earlier than 21 days and no later than 56 days post-surgical resection.

Stratification

1. Lymph node involvement
   1. 1-3 involved lymph nodes.
   2. 4 or more involved lymph nodes.
2. Histology
   1. High (poorly differentiated or undifferentiated)
   2. Low (well or moderately differentiated)
3. Preoperative serum CEA
   1. < 5.0 ng/ml
   2. ≥ 5.0 ng/ml
   3. Unknown

Registration: Registration will be accepted only through institutions with direct registration privileges. Registration must occur prior to initiation of therapy. Confirm eligibility criteria (Sec. 5.0). Call the CALGB Registrar (919-286-4704, Monday-Friday, 9 AM-5 PM Eastern Time).
NCCTG, NCIC CTG, ECOG, SWOG and CTSU Institutions see Section 6 for randomization instructions.
EPP Institutions see Appendix V for randomization instructions.

TABLE OF CONTENTS

SECTION .... PAGE

1.0 INTRODUCTION 6

2.0 OBJECTIVES 9

3.0 INCLUSION OF WOMEN AND MINORITIES 10

4.0 ON-STUDY GUIDELINES 10

5.0 ELIGIBILITY CRITERIA 11

6.0 RANDOMIZATION, STRATIFICATION, DATA SUBMISSION, AND SAMPLE PROCUREMENT & SUBMISSION 11

7.0 REQUIRED DATA 23

8.0 TREATMENT PLAN 24

9.0 DOSE MODIFICATIONS AND MANAGEMENT OF TOXICITY 26

10.0 DRUG FORMULATION, AVAILABILITY, AND PREPARATION 31

11.0 ANCILLARY THERAPY 34

12.0 REMOVAL OF PATIENTS FROM PROTOCOL THERAPY 35

13.0 STATISTICAL CONSIDERATIONS 36

14.0 ADVERSE EVENT (AER) REPORTING 37

15.0 REFERENCES 45

16.0 MODEL CONSENT FORM 48

APPENDIX I:....... MOLECULAR MARKERS OF PROGNOSIS IN STAGE III COLON CARCINOMA

APPENDIX II:.. CALGB DATA COLLECTION PACKAGE

APPENDIX III:.. CLINICAL TRIALS AGREEMENT (CTA) LANGUAGE

APPENDIX IV:.. A PROSPECTIVE STUDY of Diet and Other Lifestyle Factors among Patients with Stage III Colon Cancer

APPENDIX V:.. EXPANDED PARTICIPATION PROJECT (EPP) Registration, Data Submission, Pathology Submission, and Adverse Event Reporting for EPP participants are included in Appendix V

APPENDIX VI:................. PATHOLOGY PRACTICE GUIDELINES FOR CALGB INVESTIGATORS

APPENDIX VII:..................... PATHOLOGIC PROGNOSTIC FACTORS IN STAGE III COLON CARCINOMA

1.0 INTRODUCTION

1.1 Disease Incidence and Prognosis

Colorectal cancers are among the most common malignant tumors in the Western world. In 1998, 131,600 new cases were expected in the United States alone (1). The disease can be highly lethal, with 56,500 deaths anticipated in this calendar year. A large subset of patients present with disease limited to the locoregional area and are able to undergo resection with curative intent (2,3,4). Many previous studies have demonstrated that patients with early stage tumors (Modified Astler-Coller stages A and B1 [T_{1-2} N_o M_o]) have a >90% 5-year survival; additional treatment postoperatively is felt to be unnecessary for this favorable prognostic group. However, 40-60% of all patients who undergo resection for potential cure are found to have more advanced disease (Modified Astler-Coller stage B2 [T_{3-4} N_0 M_0] or C [T_x N_{1-2} M_o]). In this population the risks of tumor recurrence are significantly higher. The 5-year disease-free survival for patients with Modified Astler-Coller stage B2 ranges from 75-85%. For all patients with Modified Astler-Coller stage C disease, 5-year survival is approximately 50% without postoperative treatment (5-19).

1.2 Standard Adjuvant Therapy

Data from several trials have established 5-FU/LV as the current standard of care for adjuvant treatment of colon cancer. The National Surgical Adjuvant Project for Great and Bowel Cancers (NSABP) C-04 trial randomly assigned 2,151 Dukes B and C patients to receive one of three postoperative treatments: 5-FU/high-dose LV on a weekly x 6 schedule for 6 cycles, the same 5-FU/LV schedule with the addition of standard dose oral levamisole, or 5-FU/levamisole, all for one year of therapy (20). The addition of levamisole to 5-FU/LV in this trial did not appear to be beneficial. The 5-FU/LV-containing arms appear to have done slightly better (not statistically significant at this point) than the non-leucovorin containing arm (5-FU/levamisole).

A four-arm trial (2x2 design) by the North Central Cancer Treatment Group (NCCTG) and the National Cancer Institute of Canada (NCIC) randomly assigned patients to 6 months of 5-FU/levamisole versus 12 months of 5-FU/levamisole, with or without the addition of low-dose leucovorin (21). The conclusions of this trial indicate that 12 months of chemotherapy is not superior to 6 months, and that three drugs (FU/LV/Lev) are not superior to two (FU/Lev) (22).

INT 0089 (CALGB 8896) compared 52 weeks of standard 5-FU/levamisole against 28 weeks of 5-FU/low-dose LV on a daily x 5 schedule, six months of 5-FU/low-dose leucovorin on a daily x 5 schedule plus levamisole, and 32 weeks of high-dose weekly LV plus 5-FU (23). Updated data submitted in abstract form to the 1998 ASCO meeting show 5-year survivals ranging from 63-67% for the four study arms, with no significant differences between the arms. The two 5-FU/LV arms (daily x 5 for 28 weeks or weekly x 32 weeks) have now both become widely accepted as the current standard adjuvant regimens for colon cancer. The weekly high-dose LV regimen for 32 weeks has been selected for the control arm of the proposed new trial in order to provide symmetry with the weekly CPT-11-containing investigational regimen.

1.3 Irinotecan (CPT-11)

CPT-11 is a semisyntheticderivative of camptothecin, a plant alkaloid obtained from the *Camptotheca acuminata* tree (24-26). CPT-11 possesses greater aqueous solubility, greater *in vitro* and *in vivo* activity, and is associated with less severe and more predictable toxicity than camptothecin (27-31). CPT-11 has demonstrated antitumor activity against metastatic colorectal cancer, both in 5-FU-refractory patients and in chemotherapy-naive patients (32-34).

1.4 Phase II Trials of CPT-11 in 5-FU-Refractory Colorectal Cancer

Data from three single-agent clinical studies (P&U studies 01, 3R, 06) involving a total of 304 previously treated patients in 51 centers also support the use of CPT-11 in the therapy of patients with metastatic cancer of the colon or rectum (34). Patients in these studies all had colorectal cancers that had recurred or progressed following treatment with at least one 5-FU-based therapy. In each study, CPT-11 was administered in repeated 6-week cycles comprising 90-minute I.V. infusions once weekly for 4 weeks, followed by a 2-week rest period. Starting doses of CPT-11 in these trials were 100, 125, or 150 mg/m^2.

In the intent-to-treat analysis of the pooled data across all three studies, 193 of the 304 patients began therapy at the starting dose of 125 mg/m^2, which is proposed for testing in the current trial. Among these 193 patients, 2 complete and 26 partial responses were observed for an overall response rate of 14.5% (95% Confidence Interval (CI), 9.5 to 19.5%) at this starting dose. An additional 53.9% (104/193) of the patients treated at a starting dose of 125 mg/m^2 achieved a best response of stable disease by formal response criteria, but many of these patients experienced substantial reductions in their total tumor burden at some time during the study. The majority of responses were observed within the first two cycles of therapy, and all of the responses were observed by the fourth cycle of therapy. The median duration of response for patients beginning therapy at 125 mg/m^2 was 5.2 months (range, 2.6 to 15.1 months).

1.5 Phase II Trials of CPT-11 in Previously Untreated Colorectal Cancer

At Memorial Sloan-Kettering Cancer Center, 41 patients with previously untreated metastatic colorectal cancer were enrolled and treated in an NCI-sponsored trial (33). There were 25 females and 16 males with a median age of 60 years (range, 19 to 84 years). Thirty-two percent (13/41, 95% CI: 18-46%) achieved partial responses. Eighteen additional patients (44%) had either a less than 50% reduction in tumor size or stable disease, and ten (24%) had disease progression as their best response. The median time to tumor progression was 4.0 months. For the 13 major responders, the median duration of response was 4.9 months. The median survival time for all patients was 10.9 months. The most common serious medical events were grade 3 or 4 late (occurring >24 hours post-infusion) diarrhea (27%); leukopenia (12%); neutropenia (20%), and nausea/vomiting (9.8%). The incidence of grade 3 or 4 late diarrhea, initially 56% (10/18), was reduced to 9% (2/23) with early and frequent use of loperamide and diphenhydramine.

The NCCTG completed enrollment in a multicenter phase II study of 31 patients with previously untreated colorectal cancer (35). There were 24 males and 7 females with an average age of 66 years (range, 32 to 81 years). In these patients the response rate was 29% (9/31, 95% CI: 13-45%); all were partial responses. An additional 16 patients (52%) had a minor response or stable disease, and six (19%) had disease progression. The median time to tumor progression was 4.4 months. For the 9 responders, the median duration of response was 4.4 months. The median survival time for all patients was 11.7 months. This protocol also demonstrated that late diarrhea and myelosupression were the most common serious toxicities. grade 3 or 4 late diarrhea was observed in 25.8% of the patients. grade 3 or 4 leukopenia and neutropenia were also observed in 25.8% of the patients.

1.6 Rationale for Combining CPT-11 with 5-FU/LV

5-FU plus LV is the most common standard care regimen for the treatment of patients with metastatic colorectal cancer in the United States. CPT-11 has demonstrated substantial clinical activity in patients with colorectal cancer, including activity in patients with no prior therapy and in patients with documented cancer progression during prior 5-FU chemotherapy. In view of these clinical observations, there is a clear rationale for combining these two agents in an attempt to achieve maximal antitumor effect with acceptable toxicity to the patient.

1.7 Phase I Study Supporting the Present Study Regimen

The combination of CPT-11 and 5-FU/LV in this study is based upon a study (MSKCC #94-46, P&U #M6475-0007) conducted by Saltz, et al., at Memorial Sloan-Kettering Cancer Center in New York (36). Each six-week cycle consisted of four weeks of treatment followed by two weeks of rest, the schedule of CPT-11 administration most extensively evaluated in North America. A total of 42 patients with solid tumors were enrolled, 38 of whom had colorectal cancer. The CPT-11 dose was initially fixed at 100 mg/m^2. The LV dose was fixed at 20 mg/m^2. 5-FU dosing was begun at 210 mg/m^2 (N=3) and escalated to 265 mg/m^2 (N=3), 340 mg/m^2 (N=3), and 425 mg/m^2 (N=6). The protocol was subsequently amended to increase dosing to 500 mg/m^2 5-FU (N=3) and later to 500 mg/m^2 5-FU with 125 mg/m^2 (N=3) or 150 mg/m^2 (N=3) CPT-11. With careful use of intensive loperamide therapy at the first sign of loose stool, diarrhea was of minimal clinical significance in this trial. No patient developed grade 3 or 4 diarrhea during the dose escalation phase of the study. Leukopenia and neutropenia proved to be dose limiting at combined doses of 150 mg/m^2 of CPT-11 and 500 mg/m^2 of 5-FU. Based on these results, the recommended phase II/III dose level was 125 mg/m^2 of CPT-11, 500 mg/m^2 of 5-FU, and 20 mg/m^2 of LV. An additional 14 patients were treated at the recommended phase II doses. Only three of these patients had grade 3 diarrhea. Neutropenic fever was acceptably low, occurring in only four of the patients. Substantial evidence of objective tumor activity was observed with 15.8% (6/38) of the colorectal cancer patients responding to treatment. It was concluded that this combination regimen of CPT-11 and 5-FU/LV can be safely administered and has evidence of promising antitumor activity.

Pharmacokinetic sampling in MSKCC 94-46 was designed to address the possibility that 5-FU might inhibit conversion of CPT-11 to its active metabolite, SN-38. Only CPT-11 was given on day 1 of the first treatment week, and pharmacokinetic samples for CPT-11 and SN-38 were obtained over 24 hours (CPT-11 alone). 5-FU/LV administration was delayed to day 2 for only this week. At week 2, 5-FU/LV was administered immediately after CPT-11 (CPT-11 then 5-FU/LV) and pharmacokinetic assessments were repeated. In the second six-week treatment cycle, 5-FU/LV was given immediately before CPT-11 (5-FU/LV then CPT-11) and a third pharmacokinetic assessment was performed. Thus, each patient had CPT-11 sampling performed with and without 5-FU/LV and with 5-FU/LV given before and after CPT-11.

No substantial differences in mean CPT-11 pharmacokinetic parameters when CPT-11 was administered with and without 5-FU/LV were observed. A small reduction in SN-38 C_{max} and AUC_{0-24} values was observed on week 2 (CPT-11 then 5-FU/LV) compared with week 1 (CPT-11 alone). However, the mean percent decrease in AUC_{0-24} values among patients was only 8% when compared with the corresponding values determined when CPT-11 was given alone. In addition, it is unlikely that 5-FU/LV altered the conversion of CPT-11 to SN-38 since SN-38 pharmacokinetic parameters determined when CPT-11 was given immediately after 5-FU/LV (5-FU/LV then CPT-11) were similar to those when CPT-11 was given alone. Based on these data, it was concluded that 5-FU/LV does not appear to exert a substantial effect on the metabolism of CPT-11 to its active metabolite.

Phase III trial in advanced disease. A Pharmacia & UpJohn-sponsored phase III trial is in progress in stage IV colorectal cancer comparing the CPT-11/5-FU/LV regimen developed by Saltz, et al. (36) with 5-FU/LV (Mayo clinic low-dose schedule) and with CPT-11 alone. Accrual to this study is continuing. An interim analysis of severe adverse events is pending.

Rationale for current adjuvant trial. The demonstration of activity of irinotecan in metastatic colorectal cancer, including FU-refractory disease, suggests that there may be a role for irinotecan in the adjuvant treatment of colorectal cancer. The rationale for this concurrent administration schedule is that by giving substantial doses of putatively non-cross-resistant chemotherapies concurrently, the ability to eradicate residual micrometastatic disease may be improved over the use of either agent alone. The proposed adjuvant study will test the effectiveness of this CPT-11/5-FU/LV regimen versus a standard 5-FU/LV regimen. Major endpoints will be disease-free survival and overall survival.

2.0 OBJECTIVES

2.1 Primary Objectives

2.1.1 To determine whether overall survival is prolonged in patients with stage III ($T_xN_{1-2}M_0$) colon cancer who are treated with a weekly adjuvant therapy regimen of irinotecan (CPT-11), fluorouracil (5-FU), and leucovorin (LV) over 30 weeks when compared to patients who are treated with a standard weekly 5-FU/LV regimen for 32 weeks.

2.1.2 To determine whether disease-free survival is prolonged in patients with stage III ($T_xN_{1-2}M_0$) colon cancer who are treated with a weekly adjuvant therapy regimen of irinotecan (CPT-11), fluorouracil (5-FU), and leucovorin (LV) over 30 weeks when compared to patients who are treated with a standard weekly 5-FU/LV regimen for 32 weeks.

2.2 Secondary Objectives

2.2.1 To prospectively assess putative prognostic markers (TS, p53 status, p21, p27, VEGF, MVD, DCC, MSI, and topo-1) and correlate their expression with disease-free survival and overall survival.

2.2.2 To prospectively assess the influence of diet, body mass index, and physical activity on the risk of cancer-recurrence and survival among patients with stage III colon cancer.

2.2.3 To assess the influence of diet, obesity, and physical activity on the risk of toxicity associated with adjuvant therapy.

2.3 To determine whether pathologic features including tumor grade; tumor mitotic (proliferation) index; tumor border configuration; host lymphoid response to tumor; and lymphatic vessel, venous vessel and perineural invasion predict outcome in this patient population.

(Please see Appendix VI for pathology companion study design, methods, and statistical considerations).

3.0 INCLUSION OF WOMEN AND MINORITIES

Women and minorities will be eligible for this study without alteration in eligibility criteria. The following table is based on results from Int-0089. We expect that the gender and minority composition of the 89803 patient group will be as follows:

	American Indian or Alaskan Native	Asian or Pacific Islander	Black, not of Hispanic Origin	Hispanic	White, not of Hispanic Origin	Total
Female	0.5%	1.0%	5%	1.5%	47%	55%
Male	0.5%	0.5%	5%	1.0%	38%	45%
Unknown	0	0	0	0	0	0
Total	1.0%	1.5%	10%	2.5%	85%	100%

HHS Racial and Ethnic Categories

American Indian or Alaskan Native: A person having origins in any of the original peoples of North America, and who maintains cultural identification through tribal affiliation or community recognition.

Asian or Pacific Islander: A person having origins in any of the original peoples of the Far East, Southeast Asia, the Indian subcontinent, or the Pacific Islands. This area includes China, India, Japan, Korea, the Philippine Islands and Samoa.

Black, not of Hispanic Origin: A person having origins in any of the black racial groups of Africa.

Hispanic: A person of Mexican, Puerto Rican, Cuban, Central or South American or other Spanish culture or origin regardless of race.

White, not of Hispanic Origin: A person having origins in any of the original peoples of Europe, North Africa, or the Middle East.

4.0 ON-STUDY GUIDELINES

4.1 General Information

This clinical trial can fulfill its objectives only if patients appropriate for this trial are enrolled. All relevant medical and other considerations should be taken into account when deciding whether this protocol is appropriate for a particular patient. Physicians should consider the risks and benefits of any therapy, and therefore only enroll patients for whom this treatment is appropriate. Although they will not be considered as formal eligibility (exclusion) criteria, as part of this decision making process, physicians should recognize that the following may seriously increase the risk to the patient entering this protocol:

- Psychiatric conditions which would prevent compliance with treatment or adequate informed consent.
- A medical condition such as uncontrolled infection or cardiac disease which, in the opinion of the treating physician, would make this protocol unreasonably hazardous for the patient.

- Active uncontrolled bacterial, viral (including HIV or clinically defined AIDS), or fungal infection.
- Allergies to fluorouracil, leucovorin, or irinotecan.

5.0 ELIGIBILITY CRITERIA

5.1 Required Tumor Parameters

5.1.1 Patients must have histologically documented adenocarcinoma of the colon. The gross inferior (caudad) margin of the primary tumor must lie above the peritoneal reflection. Tumor must have been completely resected, including negative radial resecting margins.

5.1.2 There must be no history of distant metastatic disease at the time of registration.

5.1.3 Pathological evaluation must show Modified Astler-Coller stage C ($T_xN_{1-2}M_0$) disease.

5.2 Prior Therapy

5.2.1 No prior chemotherapy or radiation therapy for treatment of this malignancy.

5.2.2 No previous or concurrent malignancy is allowed except for adequately treated basal cell or squamous cell skin cancer, in situ cervical cancer, or other cancer for which the patient has been disease-free for five years.

5.3 Patient Characteristics:

5.3.1 Age ≥18 years.

5.3.2 Zubrod performance status of 0-2.

5.3.3 Non-pregnant and not nursing, as chemotherapy is thought to present substantial risk to the fetus/infant. Men and women of reproductive potential may not participate unless they have agreed to use an effective contraceptive method while in this study.

5.4 Required Initial Laboratory Values:

Granulocytes	≥ 1,500/µl
Platelet count	≥ 100,000/µl
Creatinine	≤ 1.5 x upper limits of normal
Bilirubin	≤ upper limit of normal

6.0 RANDOMIZATION, STRATIFICATION, DATA SUBMISSION, AND SAMPLE PROCUREMENT & SUBMISSION

6.1 **Informed Consent:** The patient must be aware of the neoplastic nature of his/her disease and willingly consent after being informed of the procedure to be followed, the experimental nature of the therapy, alternatives, potential benefits, side-effects, risks, and discomforts. Human protection committee approval of this protocol and a consent form is required.

6.2 Patients must be randomized (and begin treatment)no later than 56 days post-surgical resection. **Patients must begin first treatment no earlier than 21 days and no later**

than 56 days post-surgical resection. No patient may begin therapy without prior study registration.

6.3 Randomization Procedures

6.3.1 CALGB Institutions

Registration will be accepted only through institutions with direct registration privileges. Registrations must occur prior to initiation of therapy. Confirm eligibility criteria (Sec. 5.0). Call the CALGB Registrar (919-286-4704, Monday-Friday, 9 AM-5 PM, Eastern Time) with the following information:

Your name
Study #
Institution #
Treating Physician
Patient' s Social Security #
Patient' s Name, I.D.#
Date of Signed Informed Consent
Race, Sex, Date of Birth
Zip code of residence
Method of payment
Diagnosis, Date of Diagnosis
Eligibility Criteria met (Sec. 5.0) (no, yes)
Stratification Factors
List prior CALGB protocols
Date of most recent Institutional Review Board approval (<1 year)

The Main Member Institution will receive a Confirmation of Registration. Please check for errors. Submit corrections in writing to CALGB Data Management Center, First Union Plaza, Suite 340, 2200 West Main Street, Durham, NC 27705.

6.3.2 NCCTG Randomization Procedures

A signed HHS 310 form must be on file for this study at the NCCTG Randomization Center before a NCCTG institution may enter a patient.

To register a patient, fax (507/284-0885) a completed eligibility checklist to the NCCTG Randomization Center between 8:00 AM and 3:30 PM Central Time, Monday through Friday. The NCCTG Randomization Center will obtain and confirm all eligibility criteria.

The NCCTG Randomization Center will then contact the CALGB Registrar to register the patient. Copies of the CALGB Confirmation of Randomization form will be provided by the NCCTG Operations Office to the referring institution.

6.3.3 NCIC CTG Randomization Procedures

The following documentation must be on file at the NCIC CTG central office prior to randomization.

For all Principal and Co-Investigators:

- A signed HPB 3005 form
- Current c.v. (updated within the past 2 years)
- Documentation of REB approval of the study and consent form and REB receipt of the investigator's brochure, copy of REB approved consent form (on institutional letterhead), REB membership (consistent with ICH and OPRR membership requirements), completed NCIC CTG participant's list, a current

Cooperative Project Assurance number, laboratory certification and lab normal values. Please note that documented annual approval of the study is required as long as patients are being followed on the study.

- In addition, all investigators must have an NCI US Investigator number or must complete and sign the FDA Form 1572 prior to activation. FDA forms should be returned by investigators directly to the NCI US, where NCI US Investigator numbers will be assigned.

Randomizations will be accepted on Monday to Friday between 8:00 AM and 5:00 PM Eastern Time. The eligibility checklist must be completed prior to randomization, The randomization may be done by telephone (613-533-6430) or by fax (613-533-2941). As soon as eligibility is ascertained (including an REB approval date within 1 year prior and the existence of a signed consent form) a call will be placed to the CALGB Registrar between 9:00 AM and 5:00 PM Eastern Time. NCIC CTG will then relay the treatment assignment to the centre and confirm it in writing.

6.3.4 ECOG Randomization Procedures

A signed HHS 310 Form, a copy of the institution's IRB-approved informed consent document and a written justification for any changes made to the informed consent for this protocol must be on file at the ECOG Coordinating Center before an ECOG institution may enter patients. These will be submitted to:

ECOG Coordinating Center
FSTRF
ATTN: IRB
303 Boylston Street
Brookline, MA, 02445.

To register an eligible patient on the study, the investigator will telephone the Randomization Desk at the ECOG Coordinating Center at 617-632-2022, Monday-Friday, between the hours of 9:00 AM and 4:30 PM ET to allow time to call the CALGB that same day. ECOG members should not call the CALGB directly. The following information will be requested:

b) Protocol Number

c) InvestigatorIdentification (including institutionand/or affiliate name and Investigators name)

d) Patient identification (including patient's name or initials, chart number, social security number)

e) Patient demographics(sex, birth date, race, nine-digit zip code, and method of payment)

f) Eligibility verification

Patients must meet all of the eligibility requirements listed in Section 5.0. The randomization specialist will verify eligibility by asking questions from the checklist, and will also verify IRB approval. The ECOG randomization Desk will then contact CALGB to enter the patient, after which the ECOG Coordinating Center will contact the institution to relay the treatment assignment for this patient. The CALGB will then forward a confirmation of treatment assignment to the ECOG Coordinating Center for routing to the ECOG participating institution.

6.3.5 SWOG Randomization Procedures

SWOG Group Member and Affiliates: Patients from Southwest Oncology Group Member and Affiliate institutions must be registered with the Southwest Oncology Group Statistical Center by telephoning 206/667-4623 between the hours of 6:30 a.m. and 1:30 p.m. (PT) Monday through Friday, excluding holidays. The

Statistical Center will confirm that the patient is eligible and will request the date informed consent was obtained and the date of IRB approval for each entry. The Statistical Center will then contact the CALGB Registrar to randomize the patient after which the Statistical Center will contact the institution to confirm registration and relay the treatment assignment for that patient. The CALGB Registrar will forward a Confirmation of Randomization to the Statistical Center for routing to the appropriate institution.

SWOG CCOP Institutions: Patients from Southwest Oncology Group CCOP institutions must be registered with the Southwest Oncology Group CCOP Office by telephoning 206/652-CCOP (206/652-2267) between the hours of 7:00 a.m. and 1:30 p.m. (PT), Monday through Fridays, excluding holidays. The CCOP Office will confirm that the patient is eligible and will request the date informed consent was obtained and the date of IRB approval for each entry. The CCOP Office will then contact the CALGB Registrar to randomize the patient after which the CCOP Office will contact the institution to confirm registration and relay the treatment assignment for that patient. The CALGB Registrar will forward a Confirmation of Randomization assignment to the Statistical Center for routing to the CCOP Office and the appropriate institution.

Please note: Southwest Oncology Group institutions will follow their normal procedures for documentation of IRB approval.

6.3.6 Registration / Randomization, CTSU Investigators:

Prior to the recruitment of a patient for this study, investigators and their institutions must be registered members of the CTSU. Each CTSU investigator or group of investigators at a clinical site must obtain IRB approval for this protocol before they can enroll patients.

CTSU Procedures for Patient Enrollment: Contact the CTSU Patient Registration Office by calling 1-888-462-3009 to alert the CTSU Patient Registrar that an enrollment is forthcoming. To enroll the patient, the investigator should complete the following forms:

- CTSU Enrollment Coversheet
- C89803 Eligibility Checklist
- CALGB Confirmation of Randomization (complete all sections except for “CALGB Patient Number”, “Eligibility Criteria”, and “Treatment”)

These forms should be faxed to the CTSU Patient Registrar at 1-888-691-8039 between the hours of 8:00 am and 4:30 p.m., Eastern time. The CTSU registrar will check the investigator and site information provided to insure that all regulatory requirements have been met. The registrar will also check the forms for completeness and follow-up with the site to resolve any discrepancies. Once investigator and patient eligibility are confirmed, the CTSU will contact the CALGB Data Management Center (DMC) to obtain a randomization assignment and assignment of a unique patient ID. The CTSU will then contact the enrolling site and convey the CALGB patient ID number (to be used on all future forms and correspondence) and the patient’s treatment assignment. This will be followed by two e-mails to the enrolling site: a CALGB-generatedConfirmation of Registration and a CALGB-generated Confirmation of Treatment.

6.4 Stratification

6.4.1 Lymph node involvement

1. 1-3 involved lymph nodes.
2. 4 or more involved lymph nodes.

6.4.2 Histology

1. High (poorly differentiated or undifferentiated)
2. Low (well or moderately differentiated)

6.4.3 Preoperative serum CEA

1. < 5.0 ng/ml
2. ≥ 5.0 ng/ml
3. Unknown

6.5 Data Submission

Submit forms to the appropriate cooperative group office listed in Section 6.5.1 at the following intervals:

	Form	Submission Schedule
C-584	GI Adjuvant Intergroup On-Study Form<br>***Prestudy Radiologic Reports***<br>***Pathology and Operative Reports****	Within one week of registration.
C-447	Tissue Sample Tracking Form - Blocks<br>*(Also include a copy of the pathology report)*	Send original with paraffin block to the Pathology Coordinating Office and send a copy to the Data Management Center. Submit within 1 month of registration.
C-586	GI Adjuvant Treatment Form	Each cycle during protocol therapy.
C-528	CALGB 89803 Toxicity Form	Each cycle during protocol therapy. After the completion of protocol therapy; submit form every 3 months for 1 year; or at recurrence; and at death.
C-585	GI Adjuvant Follow-up and Recurrence Form	Each cycle during protocol therapy. After the completion of protocol therapy; submit form every 3 months for 2 years, then every 4 months for 2 years, and at the beginning of year 5; annually for 3 years, or at recurrence, and at death.
	Food Questionnaire	At the beginning of the third cycle of treatment; 6 months following the end of treatment.
	Pathology Reports*<br>***Radiology Reports***	At any re-evaluation of patient's disease.
C-300	CALGB: Off Treatment Notice	At end of all protocol treatment.
C-215	CALGB Secondary Malignancy Form	At second malignancy.
C-113	CALGB Notification of Death Form	At time of death.

* Legible copies of institutional operative notes and pathology reports must be submitted to the CALGB Data Management Center with the other required CALGB forms.

6.5.1 Data Form Submission Data forms should be submitted to the following cooperative group offices according to the schedule outlined in Section 6.5.

CALGB Institutions submit forms to:

CALGB Statistical Center
Hoch Plaza,
2424 Erwin Road, Suite 802
Durham, NC 27705

NCCTG Institutions: The original data forms as listed in this section should be submitted at the required intervals to the NCCTG Operations Office.

NCCTG Operations Office
200 First Street Southwest
Rochester, MN 55905

Include the CALGB study number and patient number. The NCCTG Operations Office will forward the forms to the CALGB Data Management Center. In addition, the CALGB Pathology Submission Form C-447 must be completed within one month of registration.

NCIC CTG Institutions submit forms to:

NCIC Clinical Trials Group
Queens University
82-84 Barrie Street
Kingston, Ontario
Canada K7L 3N6

A single set of case report forms (CRFs) will be sent to each centre (for photocopying and use) following local activation. CRFs should be completed and submitted to the NCIC CTG central office according to the submission schedule in Section 6.5. In addition to the required forms as listed, a copy of the signed consent form must be submitted for each patient. The CALGB and NCIC CTG patient number as well as patient initials must be recorded on each form. CRFs will be forwarded to CALGB by the NCIC CTG.

ECOG Institutions:

The original Data forms as listed in Section 6.5 should be submitted at the regular intervals to the ECOG coordinating center at the following address:

ECOG Coordinating Center
FSTRF
ATTN: IRB
303 Boylston Street
Brookline, MA, 02445.

Include the CALGB and ECOG study and patient numbers. The ECOG Coordinating Center will the forward the forms to the CALGB.

Do not use ECOG forms for this study, with the exception of the Adverse Reaction (ADR) Form (#391RF) and ECOG Pathology Submission Form (#638).

SWOG Institutions:

Group Members and Affiliates: Two copies of the original data forms as listed in Section 6.5 must be submitted at the required intervals for forwarding to the CALGB Data Management Center:

The Southwest Oncology Group Statistical Center
Fred Hutchinson Cancer Research Center
1100 Fairview Avenue North
MP-557, P.O. Box 19024
Seattle, WA 98109-1024

Include the CALGB and SWOG protocol numbers and patient numbers on each page of data.

CCOP Institutions: Two copies of the original data forms as listed in Section 6.5 should be submitted at the required intervals to:

Cancer Research and Biostatistics (CRAB)
ATTN: SWOG CCOP Office
1100 Olive Way, Suite 1150
Seattle, WA 98101-1892

Include the CALGB and SWOG protocol numbers and patient numbers on each page of data. The Southwest Oncology Group CCOP Office will forward a copy to the CALGB Data Management Center.

CTSU Institutions

Data Forms and Reports: All data forms for this study are available for download from the CTSU website. CTSU investigators should use the protocol-specific CALGB forms and adhere to the CALGB schedule for data submission, including the submission of radiology, pathology, and operative reports. A CTSU data form should accompany all forms and reports submitted to the CTSU.

With the exception of the patient registration forms (which should be faxed), all original forms and reports must be mailed directly to the CTSU. The CTSU will forward all information to the CALGB.

Mail forms or data to:

CTSU Data Processing Manager
CTSU Data Center
WB 408
1441 W. Montgomery Avenue
Rockville, MD 20850-2062

6.5.2 Treatment Codes

1. Patient is receiving protocol therapy.
5. Patient has discontinued protocol therapy and is in follow-up.
6. Patient is receiving non-protocol therapy.

6.5.3 Common Toxicity Criteria: This study will utilize the Common Toxicity Criteria version 2.x for toxicity and adverse event reporting. A copy of the CTC version 2.X can be accessed from the CTEP home page (http://ctep.info.nih.gov).

6.6 Sample Procurement

Blocks will be carefully banked and processed for this study according to routine CALGB Pathology Coordinating Office (PCO) paraffin tissue bank policy procedures detailed below. The policy includes the following four components:

a) Safeguards to address medical/legal concerns of submitting institutions' pathologists;

b) Quality control of storage and sectioning of blocks;

c) Quality assurance of stored/sectioned material;

d) Scientific review process for utilization of specimens.

We respect and are sensitive to the medical/legal concerns of submitting institutions' pathologists and have developed CALGB pathology policy that incorporates the following criteria:

1. All precautions are taken to prevent exhausting the tissue block.

2. A minimum of three H & E sections (obtained at different thicknesses throughout the block) remain on file at the CALGB PCO.

3. A minimum of two unstained sections (4 micron thickness) remain on file at PCO.

4. Unused portions of blocks are stored at the CALGB PCO, unless hospital policy prohibits such storage.

5. Unused portions of blocks, H & E slides, and unstained slides are available to the submitting institution by overnight carrier for any emergent medical or legal need.

The CALGB has also instituted special considerations for the small percent (5%) of hospitals whose policy prohibits long-term storage of blocks, and the smaller percentage (4%) of hospitals whose policies prohibit release of any block. Specific questions concerning the processing and storage of blocks should be directed to The CALGB Pathology Coordinating Office at the Ohio State University.

The treating institutional pathologist should screen and select the appropriate blocks for submission.

Slides will be prepared and/or routed from the CALGB PCO to the laboratory companion investigators. Please see Appendix I for details.

6.7 Sample Submission

6.7.1 CALGB Institutions

The following are required to be submitted to:

CALGB Central Pathology Office
The Ohio State University
B054 Graves Hall
333 West 10th Ave.
Columbus, OH 43210-1239
Phone: (614) 688-3495
Fax: (614) 292-5618

1. At least one (three if possible) paraffin embedded block(s) with REPRESENTATIVE TUMOR properly identifying:
   a. Patient's name and institution
   b. CALGB patient number
   c. CALGB study number
2. One paraffin embedded block with NORMAL COLONIC MUCOSA OR UNINVOLVED LYMPH NODE properly identifying:
   a. Patient's name and institution
   b. CALGB patient number
   c. CALGB study number
3. Original completed CALGB Form C-447. **A copy of Form C-447 is to be submitted to the CALGB Data Management Center.**
4. A copy of the responsible pathologist's pathology report from the TREATING institution, and, if applicable, the REFERRING institution.
5. A copy of the operative report.

6.7.2 NCCTG Institutions

NCCTG members will forward a portion of their blocks to the NCCTG Tissue Bank Repository at the NCCTG Research Base. The NCCTG Operations Office Pathology Coordinator will forward the blocks to the CALGB who will, at the completion of all translational research, return any unused tissue to the NCCTG Operations Office. CALGB will notify NCCTG in the event a block has been depleted. All residual tissue will be retained at the NCCTG Research Base but will be accessible by NCCTG members upon request. NCCTG will notify the submitting NCCTG institutional pathologist in the event a block has been depleted.

6.7.3 NCIC CTG Institutions

Following randomization, the Queen's University Pathology Department will request submission of blocks as described in Section 6.7.1. The blocks are to be clearly marked with the patient's initials and NCIC CTG and CALGB patient and study numbers. DO NOT INCLUDE PATIENT NAMES ON THE BLOCKS. The blocks should be sent with a completed CALGB Form C-447 and pathology and operative reports to the Queen's University Pathology Department. These will be forwarded to CALBG--do not submit blocks directly to CALGB.

6.7.4 ECOG Institutions:

Paraffin blocks of the tumor tissue and normal tissue will be required for tissue banking review. The blocks, along with the completed ECOG Pathology Material Submission Form (#638), the CALGB Pathology Submission Form (C-447), and the institutional pathology report should be submitted **within one month of study entry.** If insufficient tissue is available following diagnostic pathology to provide the paraffin block, a letter stating this must be sent to the ECOG Pathology Coordinating Office within 6 months of patient registration. The blocks should be sent to the ECOG Pathology Coordinating Office at:

ECOG Pathology Coordinating Office
Evanston Hospital, Room B624
2650 Ridge Avenue
Evanston, IL 60201

Include the ECOG and CALGB and patient identification numbers. The ECOG Pathology Coordinating Office will forward the slides from the blocks to CALGB Pathology Coordinating Office for the correlative studies outlined in Appendix I and VII of the protocol. A copy of the completed submission forms will be sent to the ECOG Coordinating Center by the Pathology Coordinating Office. The submitting pathologist should be informed that the blocks submitted for this protocol will be banked for further laboratory studies, and therefore, will not be returned to the submitting institution unless requested. In order to facilitate tissue collection, ECOG is requesting that when possible, extra tissue/blocks of tumor be made for patients entering the study.

NOTE: Those institutions that are unable to submit blocks should call the ECOG Pathology Coordinating Office at 847-570-1133 for slide preparation instructions.

NOTE: ECOG institutions should not send pathology materials directly to the CALGB.

6.7.5 SWOG institutions:

Southwest Oncology Group institutions should follow the Pathology Submission guidelines for CALGB institutions outlined in Section 6.7.1.

All pathology reports and routing forms should be sent directly to the CALGB Data Management Center. In addition, all Pathology Materials should be sent directly to the CALGB Pathology Coordinating Office.

6.7.6 CTSU Institutions:

CTSU institutions should follow the Pathology Submission guidelines for CALGB institutions outlined in Section 6.7.1.

Pathology specimens should *not* be sent to the CTSU. Submit the following materials and forms to the CALGB Central Pathology Office at the address below within 1 month of registration:

CALGB Central Pathology Office
The Ohio State University
B054 Graves Hall
333 West 10th Ave.
Columbus, OH 43210-1239
Ph: 614-688-3495
Fax: 614-292-5618

Copies of the CALGB Form C-447, operative report, and of the responsible pathologists' report from the treating institution, and, if applicable, from the referring institution, should also be sent to the CTSU.

6.8 Pathologic and Histologic Studies

In order to meet the histopathological objectives of this protocol, submitting institutional pathologists are expected to specify in their pathology reports which of the following histologic factors is present in the patient's tumor:

- Extracellular mucin (>50% of tumor mass)
- Small blood/lymphatic vessel invasion
- Extramural invasion
- Perineural invasion
- Infiltrating (non-pushing) tumor border
- Peri-tumoral host lymphoid response

7.0 REQUIRED DATA

Patients must be randomized (and begin treatment) no later than 56 days post-surgical resection. **Patients must begin first treatment no earlier than 21 days and no later than 56 days post-surgical resection.** No patient may begin therapy without prior study registration. Unless excepted in the table below, pre-therapy tests must be completed as follows:

Guidelines For Pre-Study Testing

To be completed within 16 DAYS before registration:
- All bloodwork, history and physical

To be completed within 90 days before registration:
- Chest x-ray

	Prior to Registration	Day 1 of each cycle	6 weeks after final dose of chemo	Post Treatment Follow up*
**Tests & Observations**				
History and Progress Notes	X	X	X	X
Physical Examination	X	X	X	X
Pulse, Blood Pressure	X	X	X	
Height/ Body surface area	X			
Weight	X	X	X	
Performance Status	X	X	X	
Drug Toxicity Assessment		Weekly**	X	
Dietary Assessment		D		E
**Laboratory Studies†**				
CBC, Differential, Platelets	X	Weekly**	X	X
Serum Creatinine, BUN	X	PRN		
AST, Alk. Phos., Bilirubin	X	PRN	X	
CEA	X		X	X
PT	X	A		
**Staging**				
Chest x-ray, PA & Lateral	X			B
Histologic Review	X			
Biopsy				C

† Lab tests completed within 14 days prior to Day 1 of Cycle 1 need not be repeated.
* At least every 3 months for 2 years, then every 4 months for 2 years, then yearly for 3 years (7 years total).
** Prior to each weekly dose of chemotherapy (not required during rest weeks).
A Weekly for patients on Arm B who are taking Coumadin.
B Annually x 5 years.
C First recurrence should be confirmed by biopsy when possible.
D At the beginning of cycle 3.
E Six months following the end of treatment.
PRN To be obtained as clinically indicated.

8.0 TREATMENT PLAN

Treatment is to begin between 21 and 56 days after the definitive surgical procedure.

Patients should be carefully monitored for evidence of thrombo-embolic disease during adjuvant treatment. Prophylactic therapy for venous thrombo-embolic disease such as mini-dose heparin or low molecular weight heparin is encouraged for patients during periods of risk for thrombotic events (such as patients with limited activity due to hospitalization). Patients with a history of coronary artery disease should be maintained on low-dose Aspirin prophylaxis if medically indicated.

8.1 ARM A

5-FU and leucovorin (4 cycles): One cycle of therapy is comprised of a six-week treatment period and a two-week rest period. Four 8-week cycles totaling 32 weeks are planned. Patients will receive leucovorin 500 mg/m^2 as a two hour intravenous infusion with 5-FU 500 mg/m^2 given as a bolus intravenous injection at one hour after initiation of leucovorin. Treatments will be given weekly for six consecutive weeks followed by a two-week rest.

8.2 ARM B

CPT-11, 5-FU, and leucovorin (5 cycles): One cycle of therapy is comprised of a four-week treatment period and a two-week rest period as outlined below. Five 6-week cycles totaling 30 weeks are planned.

8.2.1 The starting dose of CPT-11 will be 125 mg/m^2 (infused over 90 minutes) given once per week on four consecutive weeks.

For the first two cycles of CPT-11 patients should remain in the treatment area for a minimum of one hour following completion of the CPT-11 infusion in case acute abdominal cramping develops.

8.2.2 The dose of LV will remain fixed at 20 mg/m^2, administered as an intravenous bolus injection immediately following each CPT-11 dose.

8.2.3 The starting dose of 5-FU will be 500 mg/m^2, given as an intravenous bolus injection immediately following each LV dose.

8.3 Concurrent Medications

8.3.1 Loperamide (Imodium®): Patients will be instructed to begin taking loperamide at the earliest signs of a poorly formed or loose stool. Loperamide should be taken in the following manner: 4 mg at the first onset of diarrhea, then 2 mg every two hours around the clock until the patient is diarrhea-free for at least 12 hours. Patients may take loperamide every four hours during the night (see Sec. 9.6)

8.3.2 Atropine: Diarrhea or abdominal cramping that occurs during or within one hour after receiving CPT-11 can be treated with atropine (0.25 to 1 mg I.V. as indicated). Patients having recurrent problems with cholinergic symptoms may receive atropine prophylactically (sc or I.V.). Additional antidiarrheal measures may be used at the discretion of the treating physician (see Sec. 9.6)

8.3.3 Antiemetics: Patients may receive dexamethasone 10 mg I.V. as a pretreatment antiemetic before irinotecan doses, unless there is a relative or absolute contraindication to corticosteroids (i.e., diabetes, known sensitivity to corticosteroids, severe muscle weakness or myalgias, etc.). Drugs such as Ativan®,

Zofran®, or Kytril® may also be used if clinically indicated. As the majority of patients on previous trials have not experienced significant nausea, antiemetics other than decadron are recommended only for those patients who demonstrate nausea and/or vomiting despite treatment with decadron.

Routine use of antiemetics prior to treatment with standard dose 5-FU and leucovorin is rarely indicated. It is recommended that patients not be given routine antiemetics for the standard arm of the study, and that antiemetics be prescribed by the treating physician as clinically indicated if a patient develops nausea and/or vomiting.

8.3.4 Anticoagulants Patients who are taking Coumadin may participate in this study; however, it is recommended the prothrombin time be monitored carefully (at least weekly). Subcutaneous heparin is permitted.

Patients should be carefully monitored for evidence of thrombo-embolic disease during adjuvant treatment. Prophylactic therapy for venous thrombo-embolic disease such as mini-dose heparin or low molecular weight heparin is encouraged for patients during periods of risk for thrombotic events (such as patients with limited activity due to hospitalization).Patients with a history of coronary artery disease should be maintained on low-dose Aspirin prophylaxis if medically indicated.

9.0 DOSE MODIFICATIONS AND MANAGEMENT OF TOXICITY

****Attention****

New dose modifications have been added for grade 2 and 3 neutropenia and diarrhea for patients enrolled on Arm B of this study. Please read the following sections carefully as the instructions for treatment have changed.

9.1 Dose Modifications During a Cycle of Therapy The following tables are to be used for dose adjustments in patients experiencing the following toxicities <u>during a cycle of therapy</u> in either arm of the study. For subsequent cycles of therapy, see instructions in Section 9.2. All dose modifications should be based on the worst preceding toxicity. <u>The dose levels are detailed in Section 9.5</u>.

9.1.1 Neutropenia and Other Hematologic Toxicities

Table 1

**Toxicity**				**Dose Modifications**
**Grade**	**ANC**		**Platelets**	**During a Cycle of Therapy†**
2	1000 to 1499/µl	**or**	50,000-74,999/µl	↓ 1 dose level*
3	500 to 999/µl	**or**	10,000-49,999/µl	Hold dose, then ↓ 1 dose level when resolved to ≤ grade 1**
4	< 500/µl	**or**	< 10,000/µl	Hold dose, then ↓ 2 dose levels when resolved to ≤ grade 1

† For subsequent cycles of therapy, see instructions in Section 9.2.

* For patients enrolled on Arm B, irinotecan/LV/5FU should be held If <u>grade 2</u> neutropenia occurs on a planned treatment day. Proceed with treatment on the following week with a one dose level reduction if toxicity has resolved. **(note**: previous schedule called for the one dose level reduction <u>without</u> the hold in treatment).

** If grade 3 or greater neutropenia occurs after receiving the FIRST DOSE of treatment for patients enrolled on Arm B, hold irinotecan/LV/5FU until toxicity is fully resolved, then proceed with a <u>two dose level reduction</u>. (**note:** previous schedule called for a one dose level reduction of gr 3 toxicity in this setting)

9.1.2 Diarrhea

Please Note: Patients must be without a diarrheal movement (over pretreatment baseline), for at least 24 hours before the next treatment is given. If diarrhea occurs within 24 hours of planned irinotecan/LV/5FU administration, then treatment is to be held that week.

Table 2

**Toxicity**		**Dose Modifications**
**Grade**	**Stools per Day over Pretreatment Baseline**	**During a Cycle of Therapy†**
2	Increase of 4-6 stools/day, or nocturnal stools	↓ 1 dose level*
3	Increase of ≥7 stools/day or incontinence; or need for parenteral support	Hold dose, then ↓ 1 dose level when resolved to ≤ grade 1**
4	Physiologic consequences requiring intensive care; or hemodynamic collapse	Hold dose, then ↓ 2 dose levels when resolved to ≤ grade 1

† For subsequent cycles of therapy, see instructions in Section 9.2.

* For patients enrolled on Arm B, irinotecan/LV/5FU should be held If grade 2 diarrhea occurs on a planned treatment day. Proceed with treatment on the following week with a one dose level reduction if toxicity has resolved. (**note**: previous schedule called for the one dose level reduction without the hold in treatment).

** If grade 3 or greater diarrhea occurs after receiving only the FIRST DOSE of treatment, hold irinotecan/LV/5FU until toxicity is fully resolved, then proceed with a two dose level reduction. (note: previous schedule called for a one dose level reduction of gr 3 toxicity in this setting)

9.1.3 Other Non-Hematologic Toxicities*

Table 3

**Toxicity**	**Dose Modifications**
**Grade**	**During a Cycle of Therapy†**
2	↓ 1 dose level
3	Hold dose, then ↓ 1 dose level when resolved to ≤ grade 1
4	Hold dose, then ↓ 2 dose levels when resolved to ≤ grade 1

* For mucositis/stomatitis decrease only 5-FU, not CPT-11. Do not decrease doses for alopecia.

† For subsequent cycles of therapy, see instructions in Section 9.2.

9.1.4 Arm A (5-FU/LV) only:

- **Omission of Week 2 Through Week 5 Dose:**

  If a patient requires omission of a week 2 through week 5 dose due to toxicity, the patient should then receive a one-week, rather than a two-week, break after the completion of six doses in an eight-week treatment course, provided that all treatment-related toxicities have resolved after the one-week recovery period. The intention is to maintain greater dose intensity over the treatment period if possible.

  If all treatment-related toxicities have not resolved (to ≤ grade 1) by the end of the one-week recovery period, then therapy should be resumed after a two-week recovery period (provided that all treatment-related toxicities have resolved at that time).

- **Omission of Week 6 Dose:**

  Some patients will develop toxicity and require omission of the week 6 dose. In these patients, the investigator should start the next course two weeks after the last delivered dose of therapy, thus shortening the course length to seven weeks (five weeks of treatment, two weeks of rest), provided that all treatment-related toxicities have resolved (to ≤ grade 1) at that time. However, all dose modification conditions still apply. **If four or more treatment cycles are shortened from six to five weeks in this manner, then one additional treatment cycle should be added to the treatment plan.**

  If all treatment-relatedtoxicities have not resolved by two weeks after the last delivered dose of therapy, then the next course of therapy should start three weeks after the last delivered dose of therapy (course length = 8 weeks: five weeks of treatment, three weeks of rest), provided that all treatment-related toxicities have resolved at that time. Patients who have not recovered by week 8 (3 weeks after the last delivered dose of therapy) will require dose reduction as outlined in Section 9.3.

- **If treatment is delayed for > 3 weeks over planned treatment breaks, call the study chair.**

9.1.5 Arm B (CPT-11/5-FU/LV) only:

- **Omission of Week 2 or Week 3 Dose:**

  If a patient requires omission of the week 2 or week 3 dose due to toxicity, the patient should then receive a one-week, rather than a two-week, break after the completion of four doses in a six-week treatment course (i.e., treat-omit-treat-treat-treat-rest or treat-treat-omit-treat-treat-rest), provided that all treatment-related toxicities have resolved after the one-week recovery period. The intention is to maintain greater dose intensity over the treatment period if possible.

  If all treatment-related toxicities have not resolved (to ≤ grade 1) by the end of the one-week recovery period, then therapy should be resumed after a two-week recovery period (provided that all treatment-related toxicities have resolved at that time).

- **Omission of Week 4 Dose:**

  Some patients will develop toxicity and require omission of the week 4 dose. In these patients, the investigator should start the next course two weeks after the last delivered dose of therapy, thus shortening the course length to five weeks (three weeks of treatment, two weeks of rest) provided that all

treatment-related toxicities have resolved (to ≤ grade 1) at that time. However, all dose modification conditions still apply. **If four or more treatment cycles are shortened from six to five weeks in this manner, then one additional treatment cycle should be added to the treatment plan.**

If all treatment-related toxicities have not resolved by two weeks after the last delivered dose of therapy, then the next course of therapy should start three weeks after the last delivered dose of therapy (course length = 6 weeks: three weeks' treatment, three weeks' rest), provided that all treatment-related toxicities have resolved at that time. Patients who have not recovered by week 6 (3 weeks after the last delivered dose of therapy) will require dose reduction as outlined in Section 9.3.

- **If treatment is delayed for > 3 weeks over planned treatment breaks, call the study chair.**

9.2 Dose Modifications for Subsequent Cycles of Therapy If a patient experiences grade 3/4 toxicity requiring a dose reduction, then the weekly chemotherapy dose will remain decreased in subsequent treatment cycles. Dose adjustments for grade 2 toxicity may be continued in the next cycle at the discretion of the treating physician.

If a mid-cycle dose reduction is made for grade 2 toxicity, and on the following week the toxicity remains stable at grade 2, then no additional dose reduction is required.

9.3 A new cycle of treatment may begin when the granulocyte count is ≥1500/µl and the platelet count is ≥ 75,000/µl and treatment-related diarrhea or stomatitis is ≤grade 1. Additional dose reductions are not required if all toxicities resolve after one week's delay in treatment (after the end of the previous cycle). A delay greater than one week will require a dose reduction. If treatment was held for more than one additional week (after the end of the previous cycle), then reduce the chemotherapy dose by 1 dose level. If treatment was held for more than two additional weeks, then reduce the chemotherapy dose by 2 dose levels and call the study chair.

9.4 Dose Reduction Levels for LV: The dose of LV will not be adjusted due to toxicity. (For patients in Arm A, it should remain at 500 mg/m^2 for all cycles; for patients in Arm B, it should remain at 20 mg/m^2 for all cycles.) LV will be given immediately prior to each 5-FU dose.

9.5 Dose Reduction Levels:

9.5.1 Arm A (5-FU/LV) criteria for dose adjustments: The starting dose of 5-FU is 500 mg/m^2, and the dose of LV is 500 mg/m^2. Subsequent doses of 5-FU can be adjusted depending upon individual patient tolerance of treatment (see Table 4). Patients should be carefully monitored for toxicity.

Dose reduction steps for 5-FU are shown in the following table.

Table 4

Dose Reduction Steps for 5-FU - Arm A*				
	Starting Dose	Dose Level - 1	Dose Level - 2	Dose Level - 3**
5-FU	500 mg/m^2	400 mg/m^2	320 mg/m^2	260 mg/m^2

* LV dose remains fixed at 500 mg/m^2 (not adjusted).
** Further dose levels (-4, -5, etc.) will be 20% dose reductions from the previous level. Notify the study chair if dose reduction reaches – 4.

9.5.2 ARM B (CPT-11/5-FU/LV) criteria for dose adjustments: The starting dose of CPT-11 is 125 mg/m^2, the starting dose of 5-FU is 500 mg/m^2, and the dose of LV is 20 mg/m^2. Subsequent doses can be adjusted depending upon individual patient tolerance of treatment (see Table 5). Patients should be carefully monitored for toxicity.

Dose reduction steps for CPT-11/5-FU are shown in the following table.

Table 5

Dose Reduction Steps for 5-FU/CPT-11 - Arm B*†				
	Starting Dose	Dose Level - 1	Dose Level - 2	Dose Level - 3**
CPT-11	125 mg/m^2	100 mg/m^2	75 mg/m^2	50 mg/m^2
5-FU	500 mg/m^2	400 mg/m^2	320 mg/m^2	260 mg/m^2

* LV dose remains fixed at 20 mg/m^2 (not adjusted).
** Further dose levels (-4, -5, etc.) will be 20% dose reductions from the previous level. Notify the study chair if dose reduction reaches – 4.
† For mucositis reduce 5-FU only, do not reduce CPT-11.

9.6 Management of Gastrointestinal Toxicity

9.6.1 Patients will be instructed to begin taking loperamide at the earliest signs of a poorly formed or loose stool. Loperamide should be taken in the following manner: 4 mg at the first onset of diarrhea, then 2 mg every two hours around the clock until the patient is diarrhea-free for at least 12 hours. Patients may take loperamide every four hours during the night. If there is no relief within 24-36 hours, add deodorized Tincture of Opium 4 drops in 1/4 to 1/2 cup of bland liquid or juice every 4 hours around the clock.

9.6.2 Atropine Diarrhea or abdominal cramping that occurs during or within one hour after receiving CPT-11 can be treated with atropine (0.25 to 1 mg I.V. as indicated). Patients having recurrent problems with cholinergic symptoms may receive atropine prophylactically (sc or I.V.). Additional antidiarrheal measures may be used at the discretion of the treating physician.

9.7 Dose Modification for Obese Patients

There is no clearly documented adverse impact of treatment of obese patients when dosing is performed according to actual body weight. Therefore, **all dosing is to be determined solely by (1) the patient's BSA as calculated from actual weight or (2) actual weight without any modification unless explicitly described in the protocol**. This will eliminate the risk of calculation error and the possible introduction of variability in dose administration. **Failure to use actual body weight in the calculation of drug dosages will be considered a major protocol deviation.** Physicians who are uncomfortable with administering chemotherapy dose based on actual body weight should not enroll obese patients on CALGB protocols.

10.0 DRUG FORMULATION, AVAILABILITY, AND PREPARATION

10.1 Qualified personnel who are familiar with procedures that minimize undue exposure to themselves and to the environment should undertake the preparation, handling, and safe disposal of chemotherapeutic agents in a self-contained, protective environment.

10.2 Discard unused portions of injectable chemotherapeutic agents that do not contain a bacteriostatic agent or are prepared with unpreserved diluents (i.e., Sterile Water for Injection USP or 0.9% Sodium Chloride for Injection USP) within eight hours of vial entry to minimize the risk of bacterial contamination.

10.3 The total administered dose of chemotherapy may be rounded up or down within a range of 5% of the actual calculated dose.

10.4 **5-Fluorouracil** (5–FU; fluorouracil; Efudex®; Adrucil®)

Availability

5-FU is commercially available as an antimetabolite that interferes with RNA and DNA synthesis. Fluorouracil Injection (Roche Laboratories): 50 mg/ml, 10ml vials; clear, yellow, aqueous solution; Fluorouracil (Cetus, Lyphomed, Americal): 50 mg/ml; 10 ml, 20 ml, 100 ml vials, 10 ml ampules; Fluorouracil (Solopak): 50 mg/ml; 10 ml, 50 ml, 100 ml vials; 10 ml ampules; Adrucil® (Adria Laboratories): 50 mg/ml, 10 ml ampules.

Preparation

Inspect for precipitate; if found, agitate or gently heat in water bath. Filter ampules with aspiration needle (5 µm). Compatible with D_5W, 0.9% NaCl, D_5LR.

Additive Incompatibility: carboplatin, cisplatin, cytarabine, diazepam, doxorubicin, droperidol, and epirubicin.

Y–Site Incompatibility: Filgrastim, Ondansetron, Vinorelbine, and Dexamethasone.

Storage and Stability

Store at room temperature and protect from light. Dark yellow color indicates decomposition. Stable in polypropylene syringes. Stable in PVC reservoirs for infusion pump for 12 days. May adsorb to glass surfaces. Stable in cellulose nitrate/acetate ester or Teflon filters.

Administration

I.V. push, usually over 1–2 minutes: does not require further dilution,.

Ensure vein patency before administration.

Toxicity

Nausea, vomiting (mild); Ileus; stomatitis: 5–8 days after treatment initiation; gastric ulceration; myelosuppression: leukopenia, granulocytopenia (9–14 days); thrombocytopenia (7–14 days); Alopecia; loss of nails; hyperpigmentation; photosensitivity; Maculopapular rash; palmar–plantar erythrodysethesias: (42–82% receiving continuous infusion); CNS effects: disorientation, confusion (rare); Cardiotoxicity: MI, angina; asymptomatic S–T changes 68%; also rare ocular effects.

Drug Interactions

Allopurinol: possible decreased effect of 5-FU.

LV enhances the cytotoxicity of 5-FU by forming a more stable tertiary complex with thymidylate synthetase.

10.5 Leucovorin Calcium (Folinic Acid) Leucovorin Calcium (calcium folinate; citrovorum factor; N 5–formyltetrahydrofolate; 5–formyl–FH_4; folinic acid; folinic acid–SF; (6RS)–folinic acid; Wellcovorin®).

Availability

Leucovorin calcium is commercially available, and is a stable reduced formyl derivative and the active form of folic acid. The following products are available:

Immunex (formally available from Lederle): 50 mg vial, 100 mg vial, 350 mg vial.

Burroughs-Wellcome (Wellcovorin®): 100 mg vial.

Chiron Therapeutics: 50 mg vial, and 100 mg vial.

Preparation

LV may be reconstituted with Bacteriostatic Water for Injection (BWI). Reconstitute 50 mg with 5 ml BWI, 100 mg vial with 10 ml BWI. Both of these will yield a solution of 10 mg/ml. Reconstitute 350 mg vial with 17 ml BWI to yield a solution of 20 mg/ml. Use bacteriostatic water only with doses <10 mg/m^2.

Storage and Stability

Unreconstituted vials are stored at room temperature and protected from light. The reconstituted 10 mg/ml or 20 mg/ml solution is stable for at least 7 days at room temperature.

Administration

Leucovorin will be administered intravenously, either by push (Arm B) or continuous infusion (Arm A).

Toxicity

The only adverse reaction for LV is rare reports of allergic reactions to parenteral injections of leucovorin. However, this is extremely uncommon.

10.6 Irinotecan (NSC #616348, IND # 42459): Irinotecan hydrochloride trihydrate [CPT-11, (4S)-4,11-diethyl-4-hydroxy-9-[(4-piperidinopiperidino)carbonyloxy]-lH-pyroano[3',4':6,7]indolzino[1,2-b]quino line-3,14(4H,12H)dione hydrochloride trihydrate] is a topoisomerase I inhibitor.

The agent (hereinafter referred to as "Agent"), Irinotecan (CPT-11), used in this protocol was provided to the NCI under a Clinical Trials Agreement (CTA) or a Cooperative Research and Development Agreement (CRADA) between Pharmacia & Upjohn Company (hereinafter referred to as "Collaborator") and the NCI Division of Cancer Treatment and Diagnosis. Therefore, the provisions in the "Intellectual Property Option to Collaborator" terms of award modifications (see http://ctep.info.nih.gov/InfoBioTech/TERMS%20OF%20AWARD%20ADDITIONS.htm) applies to the use of the Agent in this study.

1. Agent may not be used for any purpose outside the scope of this protocol, nor can Agent be transferred or licensed to any party not participating in the clinical study. Neither the institution nor the investigator shall charge any third party

payer or patient enrolled in the study for the Agent, nor shall the institution or investigator include the cost of such drug in any cost report to third party payers. Collaborator data for Agent are confidential and proprietary to Collaborator and shall be maintained as such by the investigators.

2. Collaborator Confidential Information includes any scientific, technical, trade or business information provided, directly or indirectly, by Collaborator which is treated by Collaborator as confidential or proprietary, which is labeled or identified as "Confidential." Confidential Information is to be used solely for the purpose of conducting the research described in the protocol. Information shall be considered Confidential Information if reduced to written summary and marked as such within thirty (30) days of disclosure.

3. TERMS OF AWARD ADDITIONS / Intellectual Property Option to Collaborator. For further information on this subject, please see http://ctep.info.nih.gov/InfoBioTech/TERMS%20OF%20AWARD%20ADDITIONS.htm

4. The results of Cancer and Leukemia Group B (CALGB) studies are published by the CALGB or its investigators in accordance with the "Guidelines for CALGB Publications" contained in the CALGB Policies and Procedures.

Availability

CPT-11 is manufactured by Pharmacia-Upjohn and distributed by the Division of Cancer Treatment and Diagnosis. CPT-11 for injection is supplied in 5 ml vials containing 100 mg of drug and 2 ml vials containing 40 mg of drug. The drug is supplied in brown vials and appears as a pale-yellow-to-yellow crystalline powder and pale yellow transparent solution when reconstituted.

Drug Ordering and Accountability

Irinotecan, NSC #616348, may be requested by the Principal Investigator (or their authorized designees) at each participating institution. Pharmaceutical Management Branch (PMB) policy requires that drug be shipped directly to the institution where the patient is to be treated. PMB does not permit the transfer of agents between institutions (unless prior approval from PMB is obtained). Completed Clinical Drug Requests (NIH-986) should be submitted to the PMB by fax (301) 480-4612 or mailed to the Pharmaceutical Management Branch, CTEP, DCTDC, NCI, 9000 Rockville Pike, EPN, Rm. 707, Bethesda, MD 20892.

Drug Inventory Records: The Investigator, or a responsible party designated by the investigator, must maintain a careful record of the inventory and disposition of all drugs received from DCTDC, using the NCI Drug Accountability Record Form. (See the NCI Investigators Handbook for procedures for Drug Accountability and Storage.)

Preparation

CPT-11 will be mixed in 500 cc of 5% Dextrose in Water, USP. Nothing else should be added to the bag. Due to possible microbial contamination during dilution, it is recommended that the reconstituted solution be used within 24 hours of reconstitution when refrigerated or within 6 hours if kept at room temperature.

Storage and Stability

Irinotecan vials must be stored in a cool, dry place, protected from light in a locked cabinet accessible only to authorized individuals. It is stable for at least three years at room temperature. Irinotecan is stable for 24 hours in glass bottles or plastic bags after reconstitution with D_5W.

Administration

Irinotecan will be infused over 90 minutes intravenously (weekly x 4 postoperative schedule). For the first two cycles of CPT-11 patients should remain in the treatment area for a minimum of one hour following completion of the CPT-11 infusion in case acute abdominal cramping develops. If abdominal cramping develops, administer atropine (see Sec. 8.3.2).

Toxicity

Virtually all phase I and II studies of CPT-11 have reported neutropenia and/or late diarrhea (diarrhea occurring more than 8 hours after CPT-11 administration) as the dose-limiting toxicities (depending on the schedule). Other commonly observed adverse events include nausea and vomiting, anorexia, abdominal cramping, alopecia, asthenia, lymphocytopenia, and anemia. Dehydration has occurred as a result of diarrhea, particularly when associated with severe vomiting. Patients may have an acute syndrome of lacrimation, diaphoresis, abdominal cramping, and early diarrhea (during or shortly after CPT-11 administration); this syndrome is thought to be cholinergically mediated. Sporadic cases of pulmonary toxicity, manifested as shortness of breath, nonproductive cough, and transient infiltrates on chest x-ray have been reported. Infrequent occurrences of mucositis or colitis (sometimes with gastrointestinal bleeding) have been observed. Occasionally, abnormalities of serum creatinine, hepatic enzymes, or thrombocytopenia have been observed. CPT-11 may cause local irritation at infusion sites. Extravasation necrosis of the skin has not been reported in U.S. studies.

Drug Interactions

Patients who are taking Coumadin (warfarin) may participate in this study; however, it is recommended the prothrombin time be monitored carefully (at least weekly). Subcutaneous heparin is permitted.

Valproic acid is known to inhibit the glucoronidation and may potentially enhance the toxicity of CPT-11. High-dose intravenous cyclosporine should not be administered concurrently with CPT-11.

11.0 ANCILLARY THERAPY

11.1 Patients should receive *full supportive care,* including transfusions of blood and blood products, erythropoietin, antibiotics, antiemetics, etc., when appropriate.

11.2 Treatment with *hormones or other chemotherapeuticagents* may not be administered except for steroids given for adrenal failure; hormones administered for non-disease-related conditions (e.g., insulin for diabetes); and intermittent use of dexamethasone as an antiemetic.

11.3 CALGB Policy Concerning the Use of Growth Factors

11.3.1 Erythropoietin (EPO)

Use of erythropoietin (EPO) in this protocol is permitted at the discretion of the treating physician.

11.3.2 Filgrastim (G-CSF) and sargramostim (GM-CSF)

Filgrastim (G-CSF) and sargramostim (GM-CSF) treatment for patients on this protocol is discouraged.

Filgrastim and sargramostim may not be used:

a. to avoid dose reductions or delays as specified in the protocol,

b. prophylactically because of concern about myelosuppressionfrom a prior cycle of chemotherapy.

For the treatment of febrile neutropenia the use of CSFs should not be routinely instituted as an adjunct to appropriate antibiotic therapy. However, the use of CSFs may be indicated in patients who have prognostic factors that are predictive of clinical deterioration such as pneumonia, hypotension, multi-organ dysfunction (sepsis syndrome) or fungal infection, as per the ASCO guidelines. Investigators should therefore use their own discretion in using the CSFs in this setting.

If filgrastim or sargramostim are used, they must be obtained from commercial sources.

12.0 REMOVAL OF PATIENTS FROM PROTOCOL THERAPY

12.1 Duration of Treatment: The scheduled duration of treatment for patients in Arm A is 32 weeks; for patients on Arm B the duration of treatment is scheduled to be 30 weeks (see Section 8.0)

12.1.1 Disease Relapse: Therapy will be discontinued if there is documentation of tumor recurrence. First recurrence should be confirmed by biopsy when possible. **Elevation in CEA alone will not be considered evidence of tumor recurrence, but should prompt appropriate investigations to rule out recurrence.**

12.1.2 Extraordinary Medical Circumstances: If, at any time the constraints of this protocol are detrimental to the patient's health and/or the patient no longer wishes to continue protocol therapy, protocol therapy shall be discontinued. In this event:

- Notify the Study Chair.
- Document the reason(s) for discontinuation of therapy on forms.
- Follow the patient for recurrence and survival for seven years after the end of treatment.

13.0 STATISTICAL CONSIDERATIONS

13.1 Sample Size and Power Estimates

Sample size and power estimates are based on overall survival (OS). OS will be measured from the beginning of treatment until death from any cause. A total of 1260 patients will be accrued to this trial. With 1260 Stage III patients there is 82% power to detect an improvement in median survival from 8.0 years with 5-FU/LV alone to 10.5 years with 5-FU/LV plus irinotecan, a hazard ratio of 1.3. (An improvement in median survival from 8.0 years with 5-FU/LV alone to 11.0 years with 5-FU/LV plus irinotecan, hazard ratio 1.38, can be detected with 90% power.) These estimates were obtained based on a one-sided logrank test at significance level 0.05, exponential survival, a 2.8 year accrual period, and 3 years of follow-up. Accrual is expected to be 37.5 patients per month (450 patients per year). Analysis of overall survival will be according to intent-to-treat. Disease-free survival and safety data will be analyzed among patients who actually received treatment according to the treatment received.

13.2 Interim Monitoring

In accordance with CALGB Policies and Procedures, this study will be monitored throughout the accrual and follow-up periods by the CALGB Data and Safety Monitoring Board (DSMB). In addition, accrual, feasibility, and overall toxicity will be monitored by the study and committee chairs.

Formal analyses of the primary efficacy endpoint, overall survival (OS), and secondary endpoint, disease-free survival (DFS), will be conducted after 15% of the expected number of deaths (53/256) are observed. From that time, interim analyses will be performed approximately every six months to coincide with DSMB reviews

Upper and lower boundaries will be monitored. Simulations by Freidlin, et.al., show that for the logrank test the loss of power or increase in Type I error associated with frequent looks at the data are acceptably small. [37] A maximum of eight interim analyses is anticipated with two of these occurring before accrual is ended.

Upper Boundaries: The Lan and DeMets analog of the O'Brien-Fleming group sequential boundary will be used to calculate boundary significance levels at each of the interim analyses to maintain the overall 5% significance level of the test. Actual p-values less than 0.005 at the time of analysis will be truncated at 0.005. This truncation should only trivially affect the power of the trial with appropriate interim boundaries less extreme than some commonly used procedures (37). Simulations will be used to estimate the actual significance level at the time of the final analysis for a maximum of eight interim looks. We expect this estimated significance level to be approximately 0.04. A total of 356 deaths are expected at the time of final analysis.

Lower Boundaries: At each interim analysis the one-sided upper 99% confidence limit will be constructed for the observed hazard ratio. If the targeted hazard ratio of 1.5 is greater than this limit consideration will be given to stopping the trial and accepting the hypothesis of no difference in median survival.

Unless one of the boundaries is reached at an interim analysis, it is anticipated that the results will continue to be monitored by the DSMB with release of the data to the Study Committee two years after accrual is closed. This is the planned time of final analysis.

The CALGB Statistical and Data Management Center will submit quarterly reports to CTEP by electronic means using the Clinical Data Update System (CDUS).

13.3 Patient follow-up and publication of results: Each patient will be followed for recurrence and survival for 7 years after the end of treatment. It is expected that a manuscript will be submitted for publication within 4 years of protocol closure. The trial will close to accrual within four weeks of reaching the targeted accrual of 1260 patients.

14.0 ADVERSE EVENT (AER) REPORTING

Investigators are required by Federal Regulation to report possible adverse drug reactions. CALGB investigators are required to notify the IDB, the CALGB Central Office, the Study Chair, and their Institutional Review Board. As a tracking mechanism, **CALGB requires investigators to route toxicity reports through the Central Office** (see below). All investigators are required to report secondary malignancies occurring on or following treatment on NCI-sponsored protocols using commercial drugs. Reporting of cases of secondary AML/MDS is to be performed using the NCI/CTEP Secondary AML/MDS Report Form. This form should be used in place of the form FDA #3500 (Medwatch) or the DCT Adverse Event Form for reporting this type of second malignancy. All other secondary malignancies should be reported using the form FDA #3500 (Medwatch). CALGB Form C-215, CALGB Notice of Second Malignancy, must also be completed for all cases of secondary malignancy.

14.1 Direct questions regarding drug therapy to the Study Chair.

14.2 CALGB Investigators:

Reporting requirements and procedures depend upon: (1) whether investigational agents are suspected of causing toxicity, (2) whether the possibility of such a toxicity was reported in the protocol, consent form, or manufacturer's literature (expected toxicity), (3) the severity or grade of the toxicity, and (4) the phase of the study. All reactions in a "reportable" category must be reported unless it is documented on follow-up forms that the treatment is definitely *not* responsible for the toxicity.

Adverse Event Reporting Table

**Phase III**	Grade 1	Grade 2	Grade 3	Grade 4	Grade 5
**Investigational•**					
Expected	Form C-528	Form C-528	Form C-528	Written*++	Phone/Written#
Unexpected	Form C-528	Written	Phone/Written#	Phone/Written++	Phone/Written+
**Commercial••**					
Expected	Form C-528	Form C-528	Form C-528	Written*	Ref. to Study
Unexpected	Form C-528	Form C-528	Form C-528	Phone/Written#	Phone/Written#

• The DCT Adverse Reaction Form for Investigational Drugs is used for reporting Phase I, II, and III studies using investigational agents. "Investigational" includes all investigational agents as well as commercially available drugs supplied by NCI under IND held by NCI.

•• The FDA form 3500 is used for reporting commercial drug toxicities.

+ Phone the Study Chair, the Central Office (773-702-9860)and NCI (301-230-2330)within 24 hours. Complete the DCT Adverse Reaction Form for Investigational Drugs and submit to the CALGB Central Office within 5 working days, either by mail or by fax (312-345-0117). In addition, toxicity must be reported in the patient's chart.

++ **Do not write or call until bilirubin > 6 X normal.**

* Grade 4 hematosuppression does not have to be reported for agents known and expected to cause hematosuppression at the dose used.

# Phone information **ONLY** to the Study Chair and to the CALGB Central Office within 24 hours. The notification of the Study Chair and to the Central Office will allow suspension/modification of the study, if the toxicity is greater than anticipated before a large number of patients are put at risk. Complete the appropriate adverse drug reaction form and submit to the CALGB Central Office within 5 working days, either by mail or by fax (312-345-0117). In addition, toxicity must be reported on the flow sheets and in the patient's chart (unless flow sheets are part of the patient's record).

The reporting of adverse reactions described in the table in the table above is in addition to and does not supplant the reporting of toxicities as part of the report of the results of the clinical trial, e.g., study summary forms, cooperative group data reporting or Clinical Trials Monitoring Service case report forms. **All adverse reactions should also be reported to your local Institutional Review Board.**

All deaths during treatment or within 30 days following completion of active protocol therapy must be reported within 5 working days.

14.3 NCCTG Investigators:

Treatment that contains both investigational and commercial agents should be reported according to the investigational guidelines. However, if the reaction is clearly a known reaction of the commercially available agent involved, it should be reported according to the commercial agent guidelines.

ADR reporting is based on the NCI Common Toxicity Criteria. Adverse reactions requiring submission of a DCT ADR form to NCCTG Operations Office must also be reported to the local IRB.

	Unknown[1]		Known[2]	Secondary AML/MDS[3]
	Grade 2-3	Grade 4-5	Grade 4-5	
Call IDB within 24 hours[4]		X		
Call NCCTG[5] within 24 hours		X		
DCT ADR Form to NCCTG within 5 working days[6]	X	X	X	
AML/MDS Report Form to NCCTG within 15 working days[6]				X

1. Reactions definitely not treatment-related should not be reported. However, a report should be submitted if there is a reasonable suspicion of drug effect.
2. Grade 4 myelosuppression need not be reported. In leukemia patients, aplasia resulting in death (grade 5) must be reported.
3. Reporting for this toxicity required during or after treatment.
4. Contact Investigational Drug Branch (IDB) 301-230-2330 (available 24 hours/day).
5. If after hours, notify NCCTG the next working day.
6. Fax or mail: NCCTG Operations Office
200 First Street SW
Rochester, MN 55905
Fax: 507/284-1902

The NCCTG Operations Office will immediately forward a copy of the ADR form to the CALGB and to the IDB.

14.4 NCIC CTG Investigators

NCIC CTG investigators are to report all adverse events as listed in Section 14.2. **In addition**, any adverse events (i.e., toxicities) considered serious and unexpected and related to protocol treatment (see definitions below) must be reported to NCIC CTG who will in turn report these events to the Therapeutic Products Programme of Health Canada,

"Serious" adverse events include any untoward medical occurrence that at any dose:

- results in death
- is life threatening
- requires hospitalization or prolongation of existing hospitalization
- results in persistent or significant disability/incapacity, or
- is a congenital anomaly/birth defect

"Unexpected" toxicities include those which are not consistent in terms of nature or severity with the protocol agent information contained in the investigator brochure or product monograph.

"Related" toxicities include any toxicities possibly, probably or definitely related to protocol treatment.

Medical and scientific judgement should be exercised in deciding whether expedited reporting is appropriate in other situations, such as important medical events that may not be immediately life-threatening or result in death or hospitalization, but may jeopardize the patient or may require intervention to prevent one of the other outcomes listed in the definition above. These should also usually be considered serious.

Adverse events must be reported on the NCIC CTG Serious Adverse Event Form and reported by telephone (613-533-6430) and/or fax (613-533-2941) within 24 hours of the event. Reporting of cases of secondary AML/MDS is to be performed using the NCI/CTEP Secondary AML/MDS Report Form* as well as the NCIC CTG Serious Adverse Event Form.

* This form should be used in place of the form FDA #3500 (Medwatch) or the DCT Adverse Event Form for reporting this type of second malignancy. All other secondary malignancies should be reported using the form FDA #3500 (Medwatch).

Adverse events will be forwarded to the CALGB Operations Office, the Investigational Drug Branch (IDB) and/or the Therapeutic Products Programme of Health Canada by NCIC CTG as required.

14.5 Adverse Event Reporting for ECOG Investigators

This study will utilize the CTC version 2.0 for toxicity and adverse event reporting. A copy of the CTC version 2.0 should be available at your institution. It can also be downloaded form the CTEP home page. All appropriate treatment areas must be reported to ECOG, the NCI and your local IRB in the manner described below:

Arm: A

Commercial Agents: Leucovorin and 5-FU

	Grade 4/5 unexpected[1]	Death Due to Rx or within 30 days of Rx[2]
ECOG ADR Form to NCI within 10 days	X	X
ECOG ADR Form to ECOG Coordinating Office within 10 days	X	X
Notify local IRB within 10 days	X	X

1 Any unexpected toxicity not reported in the litterature or the package insert must be reported.

2 Any death from any cause while a patient is receiving treatment on this protocol or up to 30 days after the last dose of protocol treatment, or any death which occurs more than 30 days after protocol treatment has ended but which is felt to be treatment related, must be reported.

ECOG suggests ADRs be reported on the Adverse Reaction Form (ADR) for Investigational Drugs (#391RF). The form must be signed by the treating investigator. However, the MedWatch (FDA Form #3500) is also acceptable for reporting ADRs on the commercial arm.

The following adverse reactions must be reported to ECOG, the NCI and your local IRB in the manner described below. Toxicities occurring on this arm should be considered investigational.

Arm: B

Investigational Agents: Irinotecan

Commercial Agents: Leucovorin and 5-FU

	Grade 2/3 unexpected[1]	Grade 4/5 unexpected[1]	Grade 4 expected[2]	Death Due to Rx or within 30 days of Rx[3]
Call to NCI within 24 hours		X		X
Call to ECOG within 24 hours		X		X
ECOG ADR Form to NCI within 10 days	X	X	X	X
ECOG ADR Form to ECOG Coordinating Office within 10 days	X	X	X	X
Notify local IRB within 10 days	X	X	X	X

1 Any unexpected toxicity not reported in the literature or the package insert must be reported.

2 Any Grade 4 expected myleosuppression need not be reported but should be documented on flow sheets.

3 Any death from any cause while a patient is receiving treatment on this protocol or up to 30 days after the last dose of protocol treatment, or any death which occurs more than 30 days after protocol treatment has ended but which is felt to be treatment related, must be reported.

CALGB will notify Pharmacia and UpJohn of all reported ADRs

ECOG requires ADRs be reported on the Adverse Reaction Form (ADR) for Investigational Drugs (#391RF). The form must be signed by the treating investigator.

The ECOG Coordinating Center will call CALGB to report telephone ADR calls. The ADR form will be forwarded to CALGB by the ECOG Coordinating Center.

Reporting of all Second Primary Cancers

	NCI/CTEP Secondary AML/MDS report form[1]
AML/MDS	X

[1] To be completed within 30 days of diagnosis of AML/MDS that has occurred during or after protocol treatment. A copy is to be sent to ECOG and to the NCI, accompanied by copies of the pathology report (and, when available, a copy of the cytogenic report).

NCI Contact information
Tel: 301-230-2330
Fax: 301-230-0159
IDB
P.O. Box 30012
Bethesda, MD
20824

ECOG Contact information
Tel: 617-632-3610
FAX: 617-632-6900
ECOG Coordinating Center
FSTRF
ATTN: IRB
303 Boylston Street
Brookline, MA, 02445.

Non-Treatment Related Toxicities:

Toxicities which fall within the definitions listed above must be reported as an ADR/second primary whether they are felt to be treatment related or not. Toxicities unrelated to treatment that do not fall within the definitions above. must be clearly documented on the CALGB 89803 Toxicity Form. This form will then be submitted to the ECOG coordinating center (ATTN: Data) according to the Data Submission table located in Section 6.5.

14.6 SWOG Adverse Drug Reaction (ADR) Reporting Guidelines

14.6.1 Reporting for Investigational Drugs, SWOG Institutions:

WITHIN 24 HOURS OF THE ADVERSE EVENT CALL THE OPERATIONS OFFICE AT 210-677-8808

Within 10 days, send to the operations office:

- A copy of the SWOG ADR Form(or the NCI/CTEP Secondary AML/MDS Report Form for reporting cases of secondary AML or MDS).
- Copies of pre-study forms, and flow sheets from pre-study through the event.
- IRB notification documentation.
- Other data as requested during telephonic report.

In addition, follow the guidelines below:

For grading reactions, use the NCI Common Toxicity Criteria version 2.0. Known toxicities are listed in the Drug Information, Background or Informed Consent Form of the protocol.

	Unknown Event[2,3]	Known Event[2,3]
Grade 1		Not to be reported as an AE/ADR. These toxicities should be documented on the toxicity form, C-528
Grade 2	Written report to IDB within 10 working days[4].	Not to be reported as an AE/ADR. These toxicities should be documented on the toxicity form, C-528
Grade 3	Written report to IDB within 10 working days[4].	Not to be reported as an AE/ADR. These toxicities should be documented on the toxicity form, C-528
Grade 4	Report by phone to IDB within 24 hours[1]. Written report to follow within 10 working days[4].	Written report to IDB within 10 working days[4] Grade 4 myelosuppression not to be reported, but should be submitted on the toxicity form, C-528.
Grade 5	Report by phone to IDB within 24 hours[1]. Written report to follow within 10 working days[4].	Written report to IDB within 10 working days[4] For patients with leukemia that develop Grade 5 aplasia, a written report is required within 10 working days.

[1] IDB telephone number available 24 hours daily: 301-230-2330 (Recorder after hours).

[2] A report shall be submitted if there is only a reasonable suspicion of drug effect. Reactions judged definitely not to be treatment related should not be reported, except that all deaths while on treatment or within 30 days of treatment must be reported. Any death more than 30 days after treatment which is felt to be treatment related must also be reported.

[3] Cases of secondary AML or MDS should be reported on the NCI/CTEP Secondary AML/MDS Report Form in lieu of the AE/ADR Reporting Form. The Operations Office will forward this form to the Statistical Center within one working day of receipt.

[4] The event should be documented on the SWOG AE/ADR form and sent to both the Investigational Drug Branch of the NCI and SWOG at the following addresses:

ATTN: ADR Program	Investigational Drug Branch
Southwest Oncology Group	P.O. Box 30012
14980 Omicron Drive	Bethesda, MD 20824-0012
San Antonio, TX 78245-3217	

14.6.2 Reporting for Commercial Drugs, SWOG Institutions:

WITHIN 24 HOURS OF THE ADVERSE EVENT CALL THE OPERATIONS OFFICE AT 210-677-8808.

Within 10 days, send to the operations office:

- A copy of the FDA Form 3500 (or the NCI/CTEP Secondary AML/MDS Report Form for reporting cases of secondary AML or MDS).
- Copies of pre-study forms, and the toxicity form, C-528, from pre-study through the event.
- IRB notification documentation.
- Other data as requested during telephonic report.

The following guidelines for reporting an AE/ADR apply to any research protocol which uses commercial anticancer agents. The following AE/ADR experienced by patients accrued to these protocols and attributed to the commercial agent(s) should be reported:

- **Any AE/ADR which is life threatening (Grade 4) or fatal (Grade 5) and unknown.[1,2,3] Any occurrence of secondary AML or MDS must also be reported[4].**
- **Any increased incidence of a known AE/ADR reported in the protocol.**
- **Any AE/ADR which is fatal (Grade 5), even though known.[3]**

[1] For grading reactions, see NCI Common Toxicity Criteria version 2.X.

[2] Known toxicities are listed in the Drug Information, Background or Informed Consent Form of the protocol.

[3] **A report shall be submitted if there is only a reasonable suspicion of drug effect.** Reactions judged definitely not treatment related should not be reported, except that all deaths while on treatment or within 30 days after treatment must be reported. Any death more than 30 days after treatment which is felt to be treatment related must also be reported.

[4] Cases of secondary AML or MDS should be reported using the NCI/CTEP Secondary AML/MDS Report Form in lieu of Form FDA-3500. The Operations Office will forward this form to the Statistical Center within one working day of receipt.

The AE report, documented on Form FDA-3500 or NCI/CTEP Secondary AML/MDS Report Form, should be mailed to the address below within 10 working days:

Investigational Drug Branch
P. O. Box 30012
Bethesda, MD 20824-0012

Send a copy of the Form FDA-3500 or NCI/CTEP Secondary AML/MDS Report Form, plus prestudy form, the toxicity form, C-528, and a copy of IRB notification to the Operations Office within 10 working days:

Southwest Oncology Group Operations Office
ATTN: ADR Program
14980 Omicron Drive
San Antonio, TX 78245-3217

14.7 Adverse Event (AE) Reporting for CTSU Investigators

This study will utilize the CTC version 2.0 for toxicity and Adverse Event (AE) reporting. A hyperlink to the CTEP home page that contains CTC information is available on the CTSU website. CTSU investigators are responsible for reporting adverse events according to the CALGB guidelines, including notification of their local IRB. All reporting should be conducted within the timeframes specified in the protocol and completed forms and reports should be faxed to the CTSU Data Center. The CTSU will review the documents to ensure that all necessary information is included and will forward them to CALGB. CALGB will then distribute the documents internally and to the appropriate regulatory agencies.

For those AE's requiring **24-hour phone notification**, the CTSU investigator is responsible for reporting the event within 24 hours to the following persons/agencies:

g) **CTSU Data Center** at 1-888-462-3009

h) **NCI Investigational Drug Branch** at 301-230-2330

i) **CALGB Study Chair (Dr. Len Saltz)** at 212-639-2501

14.7.1 Secondary Malignancies Reporting for CTSU Investigators:

All CTSU investigators are required to report secondary malignancies occurring on or following treatment on NCI-sponsored protocols. Events should be reported according to the CALGB guidelines and conducted within the timeframes specified in the protocol. Completed forms should be submitted to the CTSU. Once received, the CTSU will send the forms to CALGB who will forward them to the appropriate agencies.

15.0 REFERENCES

1. Landis SH, Murray T, Bolden S, Wingo PA. Cancer Statistics, 1998. *Ca Cancer J Clin* 48:6-29, 1998.

2. August DA, Ottow RT, Sugarbaker PH. Clinical perspectives on human colorectal cancer metastases. *Cancer Metastases Rev* 3:303-324, 1984.

3. Willett CG, Tepper JE, Cohen AM. Failure patterns following curative resection of colonic carcinoma. *Ann Surg* 200:685-690, 1984.

4. Minsky BD, Mies C, Rich TA, et al. Potentially curative surgery of colon cancer: Patterns of failure and survival. *J Clin Oncol* 6:106-118, 1988.

5. Higgins GA, Humphrey EW, Juler GL, et al. Adjuvant chemotherapy in the surgical treatment of large bowel cancer. *Cancer* 38:1461, 1976

6. Gastrointestinal Tumor Study Group: Adjuvant therapy of colon cancer: results of a prospectively randomized trial. *N Engl J Med* 310:737-742, 1984.

7. Buyse M, Zeleniuch-Jacquotte A, Chalmers TC. Adjuvant therapy of colorectal cancer. Why we still don't know. *JAMA* 259:3571-3578, 1988.

8. Wolmark N, Fisher B, Rockette H, et al. Postoperativeadjuvant chemotherapy or BCG for colon cancer: results from NSABP Protocol C0-1. *J Natl Cancer Inst* 80:30-36, 1988.

9. Verhaegen H, DeCree J, DeCock W, et al. Levamisole therapy in patients with colorectal cancer, in Terry WD, Rosenberg SA (eds.): *Immunotherapy of human cancer*. New York, N.Y. Elsevier, pp. 225-229, 1982.

10. Laurie JA, Moertel CG, Fleming TR, et al. Surgical adjuvant therapy of large bowel carcinoma: an evaluation of levamisole and the combination of levamisole and fluorouracil. *J Clin Oncol* 7:1447-1456, 1989.

11. Moertel CG, Fleming TR, Macdonald JS, et al. Fluorouracil plus levamisole as effective adjuvant therapy after resection of stage III colon carcinoma: a final report. *Ann Intern Med* 122:321-326, 1995.

12. NIH Consensus Conference. Adjuvant therapy for patients with colon and rectal cancer. *JAMA* 264:1444-50, 1990.

13. Moertel CG, Fleming TR, Macdonald JS, et al. Intergroup study of fluorouracil plus levamisole as adjuvant therapy for stage II/Dukes' B2 Colon Cancer. *J Clin Oncol* 13:2936-2943, 1995.

14. Willet CG, Tepper JE, Cohen AM. Obstructive and perforative colonic carcinoma: patterns of failure. *J Clin Oncol* 3:379-384, 1985.

15. Takimoto CH. Enigman of fluorouracil and levamisole. J *Natl Cancer Inst* 87:471-473,1995.

16. Mayer RJ. Does adjuvant therapy work in colon cancer? *N Engl J Med* 322:399-401, 1990.

17. Moertel CG. Chemotherapy of colorectal cancer. *N Engl J Med* 1994.

18. Wolmark N, Rockette H, Fisher B, et al. The benefit of leucovorin-modulated fluorouracil as postoperative adjuvant therapy for primary colon cancer: Results from National Surgical Adjuvant Breast and Bowel Project Protocol CO-3. *J Clin Oncol* 11:1879-1887, 1993.

19. International Multicenter Pooled Analysis of Colon Cancer Trials Investigators. Efficacy of adjuvant fluorouracil and folinic acid in colon cancer. *Lancet* 345:939-44, 1995.

20. Wolmark N, Rockette H, Mamounas EP, et al. The relative efficacy of 5-FU + leucovorin, 5-FU + levamisole, and 5-FU + leucovorin + levamisole in patients with Dukes' B and C carcinoma of the colon: first report of NSABP C-04. *Proc Am Soc Clin Oncol* 15:205, 1996.

21. O'Connell MJ, Laurie JA, Shepard L, et al. A prospective evaluation of chemotherapy duration and regimen as surgical adjuvant treatment for high-risk colon cancer: a collaborative trial of the north central cancer treatment group and the national cancer institute of Canada clinical trials group. *Proc Am Soc Clin Oncol* 15:209, 1996.

22. O’Connell MJ, Laurie JA, Kahn MJ, et al. Prospectively Randomized Trial of postoperative adjuvant chemotherapy in patients with high-risk colon cancer. *J Clin Oncol* 16:295-300, 1998.

23. Haller DG, Catalano PJ, MacDonald JS, et al. Fluorouracil (FU), Leucovorin (LV), and Levamisole (LEV) adjuvant therapy for colon cancer: preliminary results of INT-0089. *Proc Am Soc Clin Oncol* 15:211, 1996.

24. Moertel CG, Schutt AJ, Reitemeier RJ, et al. Phase II study of camptothecin (NSC-100880). *Cancer Chemother Rep* 56:96-101, 1972.

25. Gottleib JA, Luce JK. Treatment of malignant melanoma with camptothecin (NSC-100880). *Cancer Chemother Rep* 56:103-105, 1972.

26. Muggia FM, Creaven PJ, Hansen JJ, et al. Phase I trial of weekly and daily treatment with camptothecin (NSC-100880): correlation with preclinical studies. *Cancer Chemother Rep* 56:515-521, 1972.

27. Yokokura T, Sawada S, Nokata K, et al. Antileukemic activity of new camptothecin derivatives. *Proceedings of the Japanese Cancer Association, 40th Annual Meeting,* Sapporo, Japan, 1981, p. 228.

28. Yokokura T, Furuta T, Sawada S, et al. Antitumor activity of newly synthesized, lactone ring-closed and water-soluble camptothecin derivative in mice. *Proceedings of the Japanese Cancer Association,* 43rd Annual Meeting, Fukuoka, Japan, 1984, p. 261.

29. Kunimoto T, Nitta K, Tanaka T, et al. Antitumor activity of 7-ethyl-10-[4-(1-piperidino)-1-piperidino]carbonyloxy-camptothecin, a novel water-soluble derivative of camptothecin, against murine tumors. *Cancer Res* 47:5944-5947, 1987.

30. Irinotecan (CPT-11) Investigator's Brochure.

31. Kawato Y, Furuta T, Aonuma M: Antitumor activity of a camptothecin derivative, CPT-11, against human tumor xenografts in nude mice. *Cancer Chemother Pharmacol* 28:192-198, 1991.

32. Rothenberg ML, Kuhn JG, Burris HA III, et al. Phase I and pharmacokinetic trial of weekly CPT-11. *J Clin Oncol* 11:2194-2204, 1993.

33. Conti JA, Kemeny NE, Saltz LB, Huang Y, Tong WP, Chou TC. Irinotecan (CPT-11) is an active agent in untreated patients with metastatic colorectal cancer. *J Clin Onc* 3:1995.

34. Von Hoff DD, Rothenberg ML, Pitot HC, et al. Irinotecan therapy for patients with previously treated metastatic colorectal cancer: Overall results of FDA-reviewed pivotal U.S. clinical trials. *Proc Am Soc Clin Oncol* 16:a803, 1997.

35. Pitot HC, Wender MJ, O'Connel, et al. A phase II trial of CPT-11 (irinotecan) in patients with metastatic colorectal carcinoma: A North Central Cancer Treatment Group (NCCTG) Study. *Proc Am Soc Clin Oncol* 13:a573, 1994.

36. Saltz LB, Kanowitz J, Kemeny NE, et al. Phase I clinical and pharmacokinetic study of irinotecan, fluorouracil, and leucovorin in patients with advanced solid tumors. *J Clin Oncol* 14:2959-67, 1996.

37. Friedlin, B. Korn, EL. George, SL. Data monitoring committees and interim monitoring guidelines. Controlled Clinical Trial 20:395-407 (1999)

16.0 MODEL CONSENT FORM

PHASE III INTERGROUP TRIAL OF IRINOTECAN (CPT-11) (NSC#616348) PLUS FLUOROURACIL/LEUCOVORIN (5-FU/LV) VERSUS FLUOROURACIL/LEUCOVORIN ALONE AFTER CURATIVE RESECTION FOR PATIENTS WITH STAGE III COLON CANCER

This is a clinical trial (a type of research study). Clinical trials include only patients who choose to take part. Please take your time to make your decision. Discuss it with your friends and family.

WHY IS THIS STUDY BEING DONE?

You are being asked to participate in this study because you have colon cancer and have had surgery to remove the cancer. The purpose of this study is to determine which of the following treatment plans is more effective in preventing the return of your colon cancer:

A) a weekly schedule of chemotherapy drugs called fluorouracil(5-FU) and leucovorin (LV) over 32 weeks;

B) a weekly schedule of 5-FU and LV plus irinotecan (CPT-11) over 30 weeks.

This research is being done because patients with colon cancer who have surgery to remove their cancer are at risk for the cancer coming back after surgery. In some patients, the risk of the cancer coming back can be reduced by giving chemotherapy drugs after the operation. The chemotherapy agent most commonly used in the treatment of colon cancer is a drug called 5-fluorouracil,often referred to as 5-FU. 5-FU is usually given by injection into a vein. Studies over the past twenty years have shown that 5-FU is perhaps better able to kill cancer cells when it is given with another drug called leucovorin (LV), and 5-FU plus leucovorin is routinely used at many centers to treat colon cancer. Unfortunately, despite the use of these drugs, the colon cancer still comes back in many patients.

The Cancer and Leukemia Group B (CALGB), in cooperation with other cancer researchers, is trying to develop better treatments for colon cancer which would result in fewer patients having their cancer come back. One approach being studied is the use of new, hopefully better, chemotherapy agents. Irinotecan (CPT-11) is a drug that has recently gained approval from the Food and Drug Administration for the treatment of colon cancers that have gotten worse despite treatment with 5-FU. Studies are now in progress to see if irinotecan is useful as a first treatment for colon cancer, either alone, or with 5-FU and leucovorin in patients with advanced, metastatic (widespread) colon cancers. This research trial will also study the safety and side effects of including irinotecan in the treatment of colon cancer.

HOW MANY PEOPLE WILL TAKE PART IN THE STUDY?

Approximately 1260 patients will participate in this study.

WHAT IS INVOLVED IN THE STUDY?

This is a randomized study, which means that two treatments are being compared with each other to find out whether one of these is better. If you agree to enter this trial, you will be randomly assigned to either one treatment or the other. You have an equal chance of assignment to either group - as in the flip of a coin. This helps to make the results of this research study more scientifically sound. Neither you nor your doctor will have a say in which arm you are assigned to, but both you and your doctor will be informed as to which treatment you are to receive. Following surgery, a rest period of at least three weeks (usually 4-6 weeks) is given, until you have recovered adequately from your operation.

You will then be treated according to whichever of the two treatment schedules below you are assigned to, either Schedule A or Schedule B:

Schedule A: You will receive standard weekly doses of 5-FU and leucovorin through a vein in your arm for 6 weeks in a row followed by two weeks with no chemotherapy treatment. This treatment cycle will be repeated three times, for a total of 32 weeks of treatment.

OR

Schedule B: You will receive doses of irinotecan, 5-FU, and leucovorin through a vein in your arm together one day per week for 4 weeks in a row, followed by two weeks with no chemotherapy treatment. This six-week cycle will be repeated four times, for a total of 30 weeks of treatment. For the first two cycles of irinotecan you will remain in the treatment area for a minimum of one hour following completion of the irinotecan infusion in case acute abdominal cramping develops.

If you take part in this study, you will have the following tests and procedures:

- Prior to beginning the study: History and physical, blood tests, and chest x-ray.
- While receiving chemotherapy:
  - Weekly blood tests
  - History and physical at the beginning of each cycle of treatment
- Six weeks after chemotherapy treatment is complete, then every 3 months for 2 years, every 4 months for 2 years, then annually for 3 years:
  History, physical, and blood tests
- Annually for 5 years after chemotherapy is complete: chest x-ray.

If at any time you have any symptoms or signs of progressive disease, your doctor will order whatever tests he/she feels is appropriate to evaluate your medical condition.

We will also ask you to fill out a questionnaire about your diet and daily activities half-way through your chemotherapy and 6 months after you complete your chemotherapy. This questionnaire should take about 30 minutes to complete.

HOW LONG WILL I BE IN THE STUDY?

You will be receiving treatment for either 30 or 32 weeks (about 7 months). In addition, you will be followed for seven years after the end of treatment unless the cancer returns.

If health conditions occur which would make your participation possibly dangerous, or if other conditions occur that would make participation harmful to you or your health, then your doctor may discontinue this treatment.

Participation is voluntary. If you choose not to participate or wish to withdraw your consent to participate in this treatment at any time, it will in no way affect your regular treatments or medical care.

WHAT ARE THE RISKS OF THE STUDY?

The treatment of colon cancer in this study makes use of drugs which are known to have side effects, some potentially very serious, but rarely fatal. While on the study, you are at risk for these side effects. You should discuss these with the researcher and/or your regular doctor. There also may be other side effects that we cannot predict. Other drugs will be given to make side effects less serious and uncomfortable. Many side effects go away shortly after the drugs are stopped, but in some cases side effects can be serious or long-lasting or permanent.

Schedule A (5-FU and LV)

Most likely side effects

- Diarrhea
- Mouth sores
- Nausea
- Vomiting
- A temporary decrease in numbers of white blood cells.
- Peeling skin on hands and feet
- Sensitivity to sunlight
- Loss of appetite
- Fatigue
- Hair loss
- Watery eyes

Less likely side effects

- Skin rash
- Blurred vision
- Loss of nails
- Headache
- Allergic reaction to LV
- Confusion
- Tingling of the fingers and toes
- Unsteadiness when walking
- Decrease in platelets (cells that help your blood clot)
- Changes in liver function tests

Very unlikely but serious side effects

- Spasms of the blood vessels that supply the heart, possibly leading to heart attacks
- Death

Schedule B (5-FU, LV, and CPT-11)

Most likely side effects

- Diarrhea
- Mouth sores
- Nausea
- Vomiting
- Sweats
- A temporary decrease in numbers of white blood cells.
- Peeling skin on hands and feet
- Sensitivity to sunlight
- Stomach cramps
- Loss of appetite
- Fatigue
- Hair loss
- Watery eyes

Less likely side effects

- Skin rash
- Blurred vision
- Loss of nails
- Anemia
- Unsteadiness when walking
- Allergic reaction
- Headache
- Confusion
- Tingling of the fingers and toes
- Decrease in platelets (cells that help your blood clot)
- Changes in kidney and liver function tests
- Inflammation of the colon

Very unlikely but serious side effects

- Spasms of the blood vessels that supply the heart, possibly leading to heart attacks
- Lung damage resulting in shortness of breath (may be permanent)

j) Blood clots

- Death

You will be monitored closely for these side effects. If symptoms develop, your physician will initiate appropriate treatment. Other unexpected side effects that have not yet been

previously observed may also occur. You must tell the study doctor about any new health problems that develop while you are participating in this study.

Because these chemotherapy drugs may affect an unborn baby, you should not become pregnant or father a baby while receiving treatment with 5-FU, LV or CPT-11. For this reason, you will be asked to practice an effective method of birth control while you are participating in this study.

ARE THERE BENEFITS TO TAKING PART IN THE STUDY?

If you agree to take part in this study, there may or may not be direct medical benefit to you. We hope the information learned from this study will benefit other patients with colon cancer in the future.

WHAT OTHER OPTIONS ARE THERE?

The standard recommended practice for your cancer is to perform the surgery you have already undergone, and then treat with one of several fluorouracil-based chemotherapy regimens for approximately 6-7 months. Arm A of this study (fluorouracil plus leucovorin, without the addition of CPT-11) is one of the most widely used standard care options, and would be one of several alternative treatments available to you if you choose not to participate in this study. Another option available to you is no therapy at this time with care to help you feel more comfortable.

There is no clear evidence that other treatments:

1) are significantly more effective than those used in this study.

2) are curative.

WHAT ARE THE COSTS?

Any procedure related solely to research which would not otherwise be necessary will be explained. Some of these procedures may result in added costs and some of these costs may not be covered by insurance. Your doctor will discuss these with you.

In addition, the use of medications to help control side-effects could result in added costs.

Investigational Agent Cost Assumption by Patient: The CPT-11 will be provided to your doctor by Pharmacia-Upjohn through the Division of Cancer Treatment and Diagnosis free of charge. If CPT-11 becomes commercially available during the course of the study, however, you or your insurance company may be asked to purchase subsequent doses of the medicine.

Fluorouracil (5-FU) and leucovorin (LV) will be obtained commercially and you will be charged as in the case of standard treatment.

In the event that complications occur as a result of this treatment, you will be provided with the necessary care. However, you will not automatically be reimbursed for medical care or receive other compensation as a result of any complications.

WHAT ARE MY RIGHTS AS A PARTICIPANT?

Taking part in the study is voluntary. You may choose not to take part or may leave the study at any time. Leaving the study will not result in any penalty or loss of benefits to which you are entitled.

We will tell you about new information that may affect your health, welfare, or willingness to stay in this study.

You may contact either the investigator in charge or a member of the human protection committee of _________ Hospital whose names and phone numbers are listed at the end of this form, if at any point during the duration of this treatment you feel that you have been:

a. inadequately informed of the risks, benefits, or alternative treatments, or

b. encouraged to continue in this study beyond your wish to do so.

WHAT ABOUT CONFIDENTIALITY?

Efforts will be made to keep your personal information confidential. We cannot guarantee absolute confidentiality. Your personal information may be disclosed if required by law.

Organizations that may inspect and/or copy your research records for quality assurance and data analysis include groups such as the CALGB (Cancer and Leukemia Research Group B), NCCTG (North Central Cancer Treatment Group), ECOG (Eastern Cooperative Oncology Group) SWOG (Southwest Oncology Group) the National Cancer Institute, CTSU (Clinical Trials Support Unit) the National Cancer Institute of Canada, Genetics Institute, Pharmacia and Upjohn, the Food and Drug Administration, or other Federal or state government agencies in the ordinary course of carrying out their governmental functions. If your record is used or disseminated for such purposes, it will be done under conditions that will protect your privacy to the fullest extent possible consistent with laws relating to public disclosure of information and the law-enforcement responsibilities of the agency.

The results of this study may be published, but individual patients will not be identified in these publications.

A record of your progress will be kept in a confidential form at ________ Hospital and also in a computer file at the statistical headquarters of CALGB (if you are participating through a CALGB institution),the NCCTG (if you are participating through a North Central Cancer Treatment Group institution), SWOG (if you are participating though a Southwest Oncology Group) CTSU (if you are participating through a Clinical Trials Support Unit institution), NCIC (if you are participating through a National Cancer Institute of Canada institution) or ECOG (if you are participating through an Eastern Cooperative Oncology Group institution).

Results of your tests, including blood samples and pathology slides, and confidential information contained in your medical record may not be furnished to anyone unaffiliated with the _________ Hospital, CALGB, ECOG, NCIC, SWOG, CTSU or NCCTG without your written consent, except as required by Federal regulation.

It may be necessary to contact you at a future date regarding new information about the treatment you have received. For this reason, we ask that you notify the institution where you received treatment on this study of any changes in address. If you move, please provide your new address to the following person:

(name)________________________________ (title)________________________

(address)_____________________________ (phone number)____________________.

WHOM DO I CALL IF I HAVE QUESTIONS OR PROBLEMS?

For questions about the study or a research-related injury, contact the researcher _______________________ at (telephone number).

For questions about your rights as a research participant, contact the ________________Institutional Review Board (which is a group of people who review the research to protect your rights) at (telephone number).

This research project and its treatment procedures have been fully explained to you. All experimental procedures have been identified and no guarantee has been given about possible results. You have had the opportunity to ask questions concerning any and all aspects of the project and any procedures involved.

Your signature indicates that you have read this form, have received acceptable answers to any questions, and willingly consent to participate. You will receive a copy of this form.

______________________________	______________________
(Patient's Signature)	(Date)
______________________________	______________________
(Physician's Signature)	(Date)
______________________________	______________________
(Name of Responsible Investigator)	(Phone #)
______________________________	______________________
(Name of IRB Representative)	(Phone #)

RELATED STUDIES

In addition to the treatment study, researchers are also interested in studying tissue, body fluids, or other specimens that were or may be obtained from you in the normal course of treatment and care. These research tests may be developed during the time you are on treatment or, in some cases, years later. We ask that you give approval for these tests to be performed using these specimens. Because it is not possible for you or the researchers conducting this study to know what will be discovered in the future and what additional tests may be appropriate at that time, we ask that you give permission for such studies without being contacted for permission for each test. These tests may provide additional information that will be helpful in understanding colon cancer or response to treatment, but it is unlikely that what we learn from these studies will have a direct benefit to you. These studies may benefit patients in the future.

In addition, tissue obtained from you may be used to establish products that could be patented and licensed. There are no plans to provide financial compensation to you should this occur. These tests will **not** involve the study of cancer genes that can be inherited (passed from parents to children). If studies of genes that can cause cancer are proposed, and you have given permission to be contacted (see below), we would contact you and ask for permission to conduct these tests at that time.

I agree that my tissue may be kept for use in research to learn about, prevent, treat or cure cancer.

________Yes ________No ________Initials

I agree that someone may contact me to ask me to take part in research that involves the study of cancer-causing genes.

________Yes ________No ________Initials

For ECOG Patients only:

My tissue may be kept for research about other health problems (for example: causes of diabetes, Alzheimers disease and heart disease).

________Yes ________No ________Initials

APPENDIX I

Molecular Markers Of Prognosis In Stage III Colon Carcinoma

A COMPANION STUDY TO CALGB 89803

Robert S. Warren, M.D.	*University of California, San Francisco*<br>Department of Surgery, Box 0790 U372<br>San Francisco, CA 94143<br>Phone: (415) 476-4089
Monica Bertagnolli, M.D.	*Cornell University/New York Hospital*<br>525 E. 68th Street, Suite F-1913<br>New York, NY 10021<br>Phone: (212) 746-2195
Max Loda, MD	*New England Deaconess Hospital*<br>110 Francis Street, Suite 3A<br>Boston, MA 02115<br>Phone: (617) 632-4001
Jeffrey F. Moley, M.D.	*Washington Univ. School Of Medicine*<br>Endocrine and Oncologic Surgery<br>660 South Euclid Avenue<br>St. Louis MO 63110<br>Phone:
Heinz-Josef Lenz, M.D.	*University of Southern California*<br>Norris Comprehensive Cancer Center<br>1441 East Lake Avenue, Room 3456<br>Los Angeles, CA 90033<br>Phone: (323) 865-3955
Richard Goldberg, M.D.	*Mayo Clinic*<br>200 1st Street SW<br>Rochester, MN 55905<br>Phone: (507) 284-2511

I. BACKGROUND AND SIGNIFICANCE

In 1998, an estimated 135,000 new cases of colorectal cancer are expected in the United States (1). Surgery is the primary modality of management for these tumors; a resection with 'curative intent' is possible in about 85% of patients. Unfortunately, more than 60,000 individuals are expected to die of the disease, usually from distant metastases (2). This high incidence of recurrent disease has led clinical investigators to recognize that the ultimate control of colorectal cancer will require both local and systemic therapy. Adjuvant therapies have, in fact, significantly improved the survival of patients with Stage III (node positive) carcinoma of the colon (3,4) Ideally, only patients who have occult metastases should receive adjuvant therapy because the drugs are toxic, expensive, and only moderately effective against advanced disease. Pathologic stage criteria do not precisely define which patients have such metastases when the primary is removed: 60-65% of stage III patients receiving current adjuvant chemotherapy will not develop metastases and will be cured. The remaining patients will recur and most will die of their disease. Thus, it would be important to more precisely identify those patients at greatest risk for recurrence who would be most likely to benefit from adjuvant therapy. We also

wish to define a group of patients who are likely to recur in spite of established adjuvant therapy and thus be candidates for novel adjuvant therapies as these develop.

The current criteria used to evaluate prognosis are stage-related and histologic parameters. Stage-related variables measure the extent of disease (depth of invasion of bowel wall, presence or absence of metastasis in regional nodes or distant sites, number of involved nodes). Histologic variables assess the state of differentiation of a neoplasm (grade), extramural vascular invasion and pre-operative serum carcinoembryonic antigen (CEA) level (5). The first widely used staging system was proposed by Dukes in 1932 (6) and assessed invasion of the bowel wall and the status of the regional lymph nodes by rectal carcinomas. Dukes did not consider grade-related criteria to affect outcome as strongly as stage-related parameters did. Thus, the TNM staging system (7) and both the Gunderson-Sosin (8) and Astler-Coller (9) modifications of the Dukes system only assess invasion of the bowel wall and status of regional lymph nodes.

These staging systems create categories too large to be useful prognosticators for individual patients and can be applied only after pathologic examination of the resected primary tumor and lymph nodes. This prompted investigators to include histologic variables in a staging system to improve prognostication. However, when histologic variables were included with the number of involved lymph nodes and degree of invasion through bowel wall in a Cox proportional hazards regression model of survival in 447 consecutive rectal carcinomas, the only independent histologic variable was lymphocytic infiltrate (5-10). Several other studies (11-14) confirmed that stage-related, but not histologic variables are independently associated with the survival of either colon or rectal carcinoma patients. In addition, within each stage, it has been difficult to identify clinically useful parameters that can stratify patients according to their risk of recurrence. This has led various investigators to examine additional genotypic or phenotypic characteristics in colon carcinoma that may be of prognostic value. These have focused on markers of cell proliferation or on features which may contribute to metastatic capacity in colon cancer such as ploidy and S phase fraction (15-19), expression of proteases or their receptors (e.g., metalloproteinases or urokinase and the uPAR) (20,21), and the expression of plasma membrane glycoproteins which may contribute to cell adhesion (CD44, sialyl Lewis a) (22,23). Most of these reports are single marker retrospective or small prospective investigations. Interpretation of these studies often has been hampered by poor quality clinical databases, lack of standardization or quality control of the laboratory methodologies, or variability of statistical methodologies (24). As pointed out recently by the Colorectal Cancer Working Group of the College of American Pathologists, the findings of these studies have not been incorporated into the design of prospective therapeutic trials (24). The strategy we have taken in this companion correlative science protocol to CALGB 89803 is to analyze the expression of a set of biological properties of the tumors or the host's response and to determine their usefulness either individually or in combination as markers of clinical outcome in patients with high-risk node-negative colon carcinoma (Stage II; MAC B2) or node-positive colon carcinoma (Stage III; MAC C). These potential prognostic markers have been selected based upon strong preliminary retrospective data and demonstrated laboratory expertise of CALGB-associated investigators. They fall into four broad categories: genomic instability (p53, microsatellite instability (MSI)), cell cycle control (p21, p27), tumor metastasis (18q LOH, VEGF and microvascular density), and responsiveness to chemotherapy (topoisomerase 1 and thymidylate synthase).

II. Preliminary Studies

1. Genomic Instability: p53 and MSI

p53: A frequently examined candidate determinant for recurrence and survival in colon cancer is the tumor suppressor gene p53. Mutations in p53 are the most common genetic abnormality found in solid tumors. p53 protein accumulation is found to predict early recurrence and clinical outcome in patients with non-small lung cancer (25,26), in node-negative breast cancer (27-29), and in carcinoma of the bladder (30). The prognostic value of p53 analysis in colorectal cancer is less clear. p53 has been analyzed indirectly by the demonstration of loss of heterozygosity of chromosome 17p (31), and directly by mutational analysis using direct sequencing or single strand conformation polymorphism determination (32). Since the half-life of mutant p53 protein is prolonged, cells harboring mutant p53 may stain positively for p53 protein by immunohistochemical techniques while cells bearing only wild-type p53 are usually negative by IHC. Consequently, some investigators have used p53 immunopositivity as a surrogate for p53 mutation and examined the relationship between p53 positivity by IHC and clinical outcome (33-42). These studies have been subject to certain criticisms due to: 1) the use of different monoclonal antibodies (DO7, 1801, p240) and polyclonal antibodies to detect the p53 protein; 2) the variable percentage of immunoreactive neoplastic cells used as a cutoff between positive and negative tumors; 3) different scoring of the immunocytochemical results according to the nuclear or cytoplasmatic compartmentalization of the p53 protein immunoreactivity;and 4) the potential instability of mutant p53 protein in formalin-fixed, paraffin-embedded tissue and employment of antigen retrieval methods or poor correlation between IHC positivity and p53 mutation (43,44). In patients with advanced colorectal cancer, response to systemic chemotherapy may be predicted by p53 status. Several studies have shown a good correlation between p53 abnormalities (mutation or protein over-expression)and diminished survival in patients undergoing curative colon resection (34,36-42), while other investigators have failed to identify a relationship between p53 status and survival in colorectal cancer (33,35). A recent study reported by Ahnen et al. analyzed the relationship between nuclear over-expression of p53 and clinical outcome using the antibody DO7 in 220 patients with either Duke's B2 (n=66) or Duke's C (n=163) cancers entered into Intergroup 0035, a study of observation vs. adjuvant 5-FU/levamisole for resected colon cancer (45). These authors found that p53 overexpression predicted for a better disease-free survival in the stage III but not in the stage II patients. p53 status might also predict treatment efficacy in patients receiving adjuvant chemotherapy similar to what has been found in patients with advanced colorectal cancer (46). A primary goal of this project is to identify patients with stage III colon cancer at significantly higher risk of recurrence and poor clinical outcome who might benefit from investigational therapies.

Anticipated results: This portion of the study will establish if the p53 nuclear overexpression will be predictive for tumor recurrence and survival. We expect that patients with p53 nuclear overexpression in their tumors have a higher risk of developing tumor recurrence and have a poor clinical outcome. Patients with no p53 nuclear expression are expected to have less recurrence and good clinical outcome. Furthermore, we expect that tumors with p53 nuclear overexpressionwill have higher TS expression; however, some tumors with no p53 nuclear staining will have high TS staining. Patients with wt p53 and low TS expression in their tumors will have the lowest risk of tumor recurrence. (See below)

Microsatellite Instability: This portion of the study addresses tumor replication error (RER) status as a potential tumor-specificpredictor of treatment outcome in colorectal cancer. Mutations altering the ability of a tissue to repair damaged DNA are important contributing factors in a number of cancers. In human colorectal cancer, altered DNA repair is characterized by mismatch repair, also known as replication

error (RER). RER can be identified by screening for alterations in inherited patterns of nucleotide repeats scattered throughout the genome. Previous studies (47-54) demonstrated that RER is present in a significant fraction of colon cancers, both in patients with and without a family history of cancer. Individuals with Hereditary Nonpolyposis Colon Cancer (HNPCC) whose tumors demonstrate this instability marker (RER+) are frequently carriers of a germline mutation in a mismatch repair gene (MMR+) (55-57). Through comparison of DNAs from tumor tissue and peripheral blood leukocytes, it is possible to identify RER and to distinguish between germline and somatic mutations in hMSH2, hMLH1, hPMS1, or hPMS2, the most frequently mutated mismatch repair genes.

The presence of intact DNA repair may also be an important prognostic indicator for colorectal cancer. In studies of colorectal cancer cell lines containing MMR gene mutations, lack of response to chemotherapeutic agents *in vitro* is found in RER+ cell lines, where RER- tumors remain sensitive. For example, HCT116 cells lack hMLH1 function, and are insensitive to 5-FU *in vitro*. When chromosome 3, which contains hMLH1, is transferred to HCT116 cells, mismatch repair function is restored, and cells are inhibited by culture in 5-FU (58). Colorectal cancer cell lines with MMR defects are resistant to methylating agents (59). Few studies have addressed the relationship between tumor RER status, tumor behavior, and cancer family history. Lothe et al. (60), using PCR-based analysis of 7 microsatellite loci, found that 17% of patients without and 31% of patients with a strong family history of cancer exhibited changes at one or more loci. Patients with RER+ tumors defined by this study exhibited increased survival when compared to their RER- counterparts. The relationship between RER status and prognosis was similar in a study of microsatellite instability in 90 colorectal tumors not selected by family history (59). In a detailed study of sporadic colorectal cancers, a strong association was observed between mucinous differentiation and RER+ phenotype (75% RER+ showed mucinous differentiation, versus 13% RER-) (61).

Preliminary studies: RER Analysis of Colorectal Cancer Hepatic Metastases. Samples liver secondaries and normal adjacent liver tissue were obtained from patients undergoing surgical exploration for isolated liver metastases of colorectal carcinoma. Following confirmation of pathology by H&E staining, DNA was extracted separately from tumor-containing and non-tumor-containing tissue. Primers for PCR amplification of 10 different variable length-repeat segments were tagged with a fluorescent marker to allow analysis of the PCR products using the Applied Biosystems 377 Automated Sequencer with GeneScan and Genotyper Software. The products of PCR were denatured and run on a non-denaturing high-resolution 10% polyacrylamide gel. RER positives were defined as cases in which particular differences in the band pattern between the DNAs from tumor and normal tissue from the same person are recognized by at least one of the four primers. The observed alterations generally involved additional CA-repeat bands, seen as band shifting on the gels. Other changes, such as the loss of one or more bands from tumor when compared with normal tissue from the same person, known as loss of CA repeats, were scored, as they may also suggest mutations in mismatch repair genes. All positives were confirmed at least twice.

Table A-1

Results of RER Analysis of Colorectal Cancer Hepatic Metastases:
Number of Tumors Showing Alterations between Tumor and Normal Tissue

Number of Loci Positive:

none	<1	<2	<3	<4
36(63%)	21(37%)	10(17.5%)	4(7%)	3(5%)

Total Number of Tumors Tested: 57

This pilot study shows that 17.5% of metastatic colorectal cancers demonstrated changes in 2 or more of the chosen microsatellite repeat segments. The patients whose tumors were tested received chemotherapy for treatment of their metastatic cancer, and the outcome results are pending. The results of this preliminary study are consistent with data obtained by other investigations (54,60,62,63) characterizing the RER status of patient populations not selected by family history.

In a study from the NCCTG, tumors from 508 patients with colon cancer undergoing adjuvant chemotherapy were characterized using 11 microsatellites on chromosomes 5,8,15,17, and 18 (17). Tumors were defined as MSS = microsatellite stable; MSI-L = <30% of markers unstable; and MSI-H = ≥30% of markers unstable. In comparing the three groups, MSI-H tumors were associated with Dukes' B stage (p=0.01) and diploid status (p=0.03), suggesting that patients with MSI-H tumors have a more favorable outcome. The clinical outcome data from this study have not yet been published.

***Anticipated Results*:** We expect that patients with tumors demonstrating microsatellite instability will have an improved disease-free and overall survival.

2. Cell Cycle Related Markers: p21 and p27

Cyclin dependent kinase inhibitors p21/WAF1/CIP1 and p27. While the presence of p53 mutations can be inferred to a large extent by immunohistochemical analysis, to date no easily measurable biochemical parameter has been identified that would be indicative of p53 function. However, recently, a gene has been identified, p21/WAF1/CIP1, which functions as a cyclin-dependent kinase (CDK) inhibitor and is thought to be a direct mediator of p53 cell-cycle regulatory activity and p53-mediated apoptosis (64,65). Transit of cells into S-phase of the cell cycle is regulated by protein kinases known as CDKs whose activity is promoted by cyclins and inhibited by a family of proteins called cyclin-dependent kinase inhibitors (CKI) (66). The CKI p21 has been shown to be induced directly by wt p53 *in vitro* but its expression is lost when active p53 is absent (65,67). These findings suggest that p21 levels maybe useful as an indicator of p53 function in cells and may be a link between p53 status and TS expression. If p21 expression is a reflection of the p53 function, low or absent expression of p21 may indicate a lack of p53 transcriptional activity, while partial expression of p21 should indicate some retention of p53 activity. p21 expression may not only help to identify p53 mutations which may have partial wild-type activity but also to identify tumors with false negative p53 staining due to deletion, non-sense and mispliced mutations. However, p21 may be regulated in a p53-independent manner through activation of the type II TGFb receptor (68). Consequently, mutations in the TGFb RII may lead to diminished p21 even in the presence of wild type p53. Similarly, preserved p21 expression may occur in the face of mutant p53 through this p53-independent pathway.

In sporadic carcinomas, a decrease of p21 expression accompanied adenoma development and progression to carcinoma. p21 was detected in 12/16 (75%) adenomas and in 10/32 (31%) of carcinomas. In contrast to the sporadic cases HNPCCs with known mutations in DNA, mismatch repair p21 was expressed in 12/15 (80%) of cancers. An inverse relationship between p21 and p53 was observed wherein mutant p53 was detected in 4/15 (27%) HNPCCs versus 22/32 (69%) sporadic cancers (69).

P27. Like p21, the CKI p27 binds to and inactivates cyclin/CDK complexes, thereby blocking progression through G1 into S phase (70). Expression of p27 protein is highest in G0 and G1 cells, and its degradation via the ubiquitin/proteosomepathway is necessary for cellular entry in the DNA replicative phase of the cell cycle. Mitogens permit this transition in part by down-regulating p27 expression (71). p27 protein expression has been examined in a semi-quantitative fashion using immunohistochemistry in several epithelial cancers. In carcinoma of the breast, esophagus and prostate and in non-small cell lung cancer, low p27 expression is associated with histologic and biochemical markers of aggressive tumor behavior and

predicts for unfavorable clinical outcome (72-75). The clinical relevance of p27 in colorectal cancer was suggested by Loda et al. (76). In a retrospective series of patients with Stage II and III colorectal carcinoma, weak (1 - 50% of tumor cells express nuclear p27) and strong (>50% of tumor cells express nuclear p27) expression are associated in a multivariate analysis with a six- and thirty-fold reduction in death from cancer compared to patients whose tumors lack p27 expression. (Fig. 1)

Anticipated Results: Patients with tumors expressing high levels of p21 or p27 will be less likely to recur and will experience better overall survival in comparison with those with low p21 or p27. We anticipate a significant correlation between low p21 and positive immuno-histochemistry for p53 (a presumed mutant genotype).

FIGURE 1

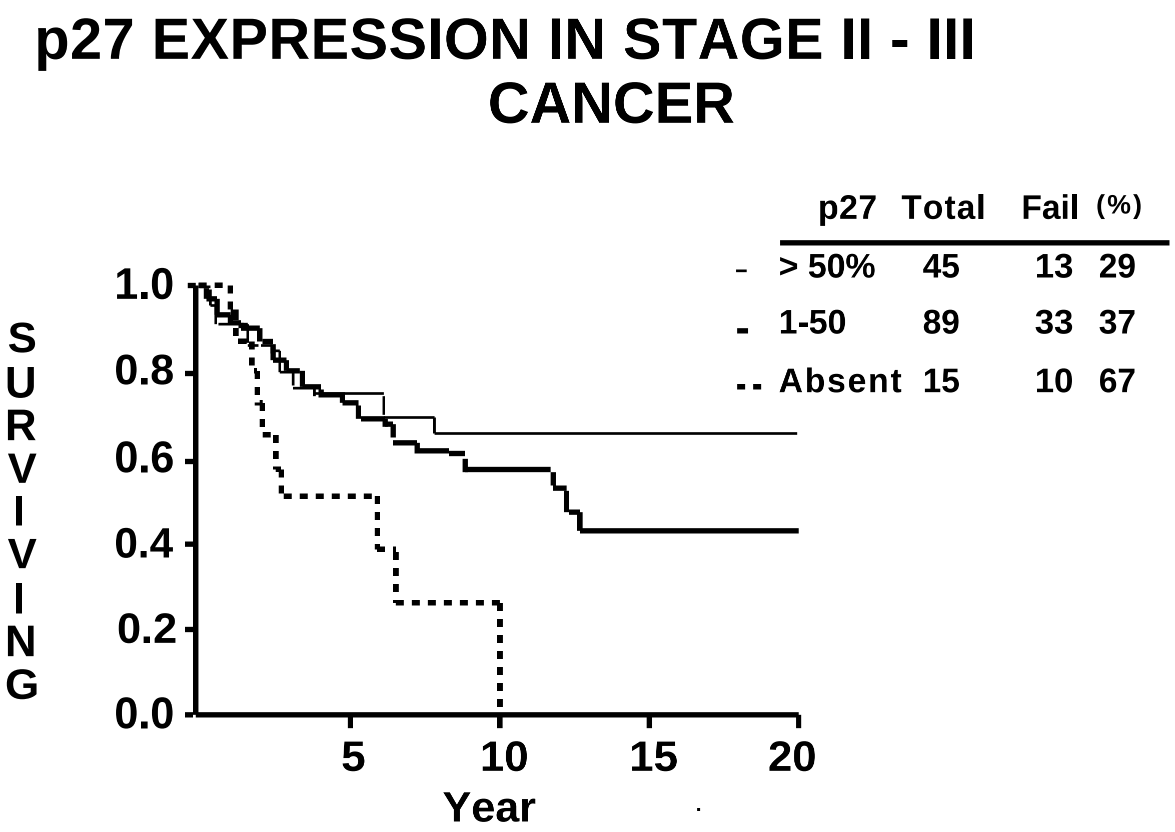

TABLE A-2

Clinicopathological Characteristics, p27 Expression, and Univariate Analysis of Outcome in 149 Patients with Colorectal Carcinoma

Variable	Category	Total (N=149)	Median Survival (Months)	P
Gender	Female	70	>272	0.232
	Male	79	88	
Age	< 74 years old	102	118	0.763
	(74 years old	47	140	
Tumor Site	Rectum	45	76	0.268
	Colon	104	151	
TNM Stage	I	32	>247	0.0001
	II	65	149	
	III	51	73	
	IV	1	0	
Tumor Differentiation	Well	9	>182	0.034
	Moderate	125	140	
	Poor	15	>95	
p27	(50%	45	>241	0.042
	1-50%	89	149	
	Absent	15	69	

Table A-2: Rectum is defined in tumor site as the large bowel distal to the peritoneal reflection. TNM staging is grouped as defined by the AJCC. p27 expression is scored by immunohistochemistry of paraffin sections as Absent (no tumor cells display any p27), 1-50% of tumor cells display p27 either in cytoplasm, nucleus or both, and more than 50% of tumor cells express p27. The actuarial analysis used a Wilcoxon test to determine p values from Kaplan-Meier curves that assessed median survivals in months (76).

3. Markers of Metastatic Potential: DCC and VEGF.

Chromosome 18q Allelic Loss. Chromosomal deletions are commonly observed in solid tumors, and regions with frequent loss are presumed to harbor potential tumor suppressor genes. Mutation generally occurs in one copy while the other allele is lost, and may be replaced by a duplicated copy of the mutant allele. As an example, allelic deletions (LOH) on 18q have been reported in up to 70% of colorectal cancers, and LOH at 18q has been detected in a variety of tumors including breast (35%-70%), gastric (61%), prostate (45%), esophageal (24%), and CNS tumors (77-82). However, the identity of the gene(s) residing on 18q which is the target of the LOH and may function as a tumor suppressor remains uncertain. Fearon and co-workers identified the gene deleted in colon cancer (dcc) as a putative tumor suppressor gene that maps to 18q21 (83). It has been suggested that DCC plays an important role in cellular differentiation. Colorectal tumors that have lost their capacity to differentiate into mucin-producing cells lack DCC expression, while well-differentiated adenocarcinomas retain DCC expression (84). Rat pheochromocytoma cells (PC12) transfected with antisense to DCC inhibit neurite outgrowth and NIH3T3 cells overexpressing DCC stimulate neurite outgrowth in neighboring PC12 cells (85). The

chicken homologue to human DCC is an epithelial adhesion molecule, which is expressed in cells involved in mesenchymal interactions. Antibodies raised against the extracellular portion of DCC inhibits cluster formation of DCC-expressing PA317 fibroblast cells. Further, in vivo administration of DCC antibodies causes disaggregation of layered embryonic chicken skin epithelial cells (86,87). More recently, DDC has been found to interact with the chemoattractant protein netrin-1 which is involved in axon outgrowth and neuronal guidance (reviewed in 88).

Initial support for the concept that DCC may function as a tumor suppressor gene derived from a variety of observations. Microcell-mediated whole chromosome 18 transfer into colon cancer cell lines reversed the malignant phenotype (89); however, it is unclear whether this suppression was due to DCC restoration or to other gene(s) on chromosome 18. DCC constructs transfected into HPV-transformed human epithelial cells suppress their malignant phenotype (90) and antisense RNA to DCC transforms Rat 1 fibroblasts indicating that loss of DCC expression may be important in the transformation of this cell type (91). A diminished level of DCC in colorectal cancers in comparison to matched normal mucosa has been reported by some, but not all, investigators(92,93), lending further support to the concept that DCC is important in colon cancer progression. However, the mechanism of loss of DCC expression is unclear. Mutations in the DCC gene are uncommon in colorectal cancers demonstrating 18q LOH (94). The loss of DCC gene expression has been secondary to abnormalities of a closely linked gene(s). In fact, two other candidate tumor suppressors genes, smad4 and smad2, map also to 18q21. These genes code for proteins involved in TGFb signaling and their role in colon cancer tumorigenesis remains to be clarified (95,96), although mutations in these genes are not common in colorectal cancer either (97). Perhaps the most compelling argument against DCC as a tumor suppressor in colon carcinogenesis comes from the study of Fazeli and co-workers. Targeted disruption of both DCC alleles in the mouse led to defects in neuronal development similar to that seen in netrin-1 knockout mice; however, no intestine-related phenotype was observed. Specifically, dcc null animals have normal intestinal epithelial growth and development and do not demonstrate an increased rate of small or large bowel tumor formation even when crossed into and APC null background (88).

Clinical studies regarding the prognostic value of DCC under-expression in colon cancer have been limited, in part due to lack of reliable techniques developed to detect DCC protein in formalin-fixed,paraffin-embedded tissue. Most studies have examined 18q LOH by PCR amplification of polymorphic microsatellite at or near 18q21, and the results remain controversial. Iino et al., in 98 colorectal samples, demonstrated that 18q LOH was significantly correlated with lymphatic invasion and hepatic metastases (98). Jen et al. retrospectively examined 69 stage II and 76 stage III colon cancer patients for 18q LOH and concluded that 18q allelic loss was strongly predictive (hazard ratio for death, 2.46; 95% CI 1.06-5.71; P=0.036) after adjustment for all other evaluated factors including tumor differentiation, vascular invasion, and TNM stage (99). More recently, both Ogunbiyi et al. and Martinez-Lopez and co-workers found that allelic loss at 18q predicted shortened disease-free and overall survival in stage II colon cancer patients (100,101) while Carethers reported that 18q21 LOH had no predictive value in seventy patients with stage II colon carcinoma (102).

Shibata and co-investigators (103) developed an immunohistochemical method for directly assessing the expression of the DCC gene product in fixed tissues. In a series of 132 colorectal cancer patients of whom 70 were stage II, it was found that lack of expression is associated with a 30% increase in recurrence compared with patients whose tumors express DCC (see Table A-3 and Fig. 2 below). Unfortunately, this affinity-purified polyclonal rabbit antibody is no longer available and other investigators have had difficulty in performing reliable IHC using paraffin-embedded archival tissue. Goi et al., however, recently reported the development of a mouse monoclonal antibody against a human dcc-GST fusion protein that appears useful in immunohistochemistry (92). Until this antibody is obtained and tested, 18q abnormalities in this protocol will be evaluated by analysis of allelic loss only.

Anticipated Results: Loss of heterozygosity for chromosome 18q by PCR techniques using microsatellite markers spanning the DCC locus on 18q will be assessed. For informative cases, we expect that patients with cancers that demonstrate LOH at 18q will have a higher recurrence rate and poorer overall survival. We also will investigate a correlation between LOH and DCC immunohistochemistry, permitting the application of the technically less complex IHC method to be adopted for routine clinical practice, if preliminary studies of new DCC antibody reagents are encouraging.

TABLE A-3

Cox Analysis of 132 Patients with Stage II-III Colorectal Cancer

Variable	Category	Risk Ratio	C.I.	p
Gender	M = 1, F = 0	1.28	0.72 - 2.28	0.40
Age	≥65 = 0, <65 = 1	0.91	0.50 - 1.63	0.74
Site	Rectum = 0, Colon = 1	0.87	0.48 - 1.58	0.64
Grade	P = 1, W/M = 0	1.77	0.39 - 8.12	0.46
Stage	III = 1, II = 0	3.11	1.70 - 5.71	0.0002
DCC	Absent=1, + = 0	3.16	1.70 - 5.85	0.0003

DCC EXPRESSION IN STAGE II - III BOWEL CANCER

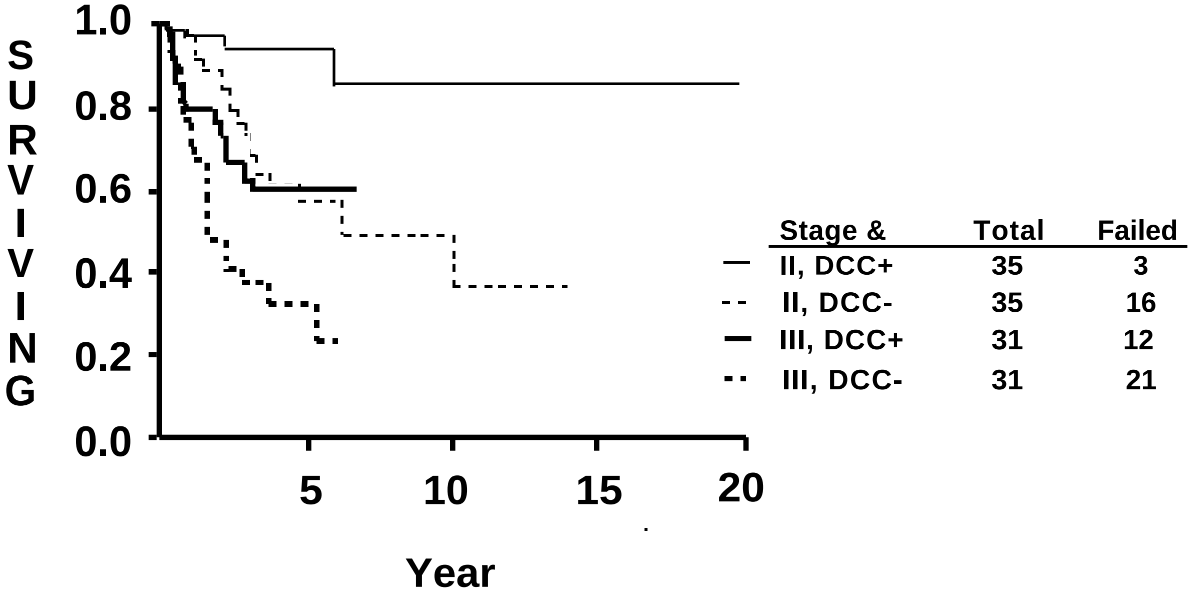

4. **Tumor Angiogenesis, Microvascular Density and Vascular Endothelial Growth Factor Expression.**

New blood vessel formation is considered a fundamental element in the capacity of tumors to grow beyond a few millimeters in greatest dimension (104). The ability of a neoplasm to induce angiogenesis appears to represent a balance between positive and negative regulatory factors (105). Inhibition of tumor endothelial cell proliferation or the inactivation of endothelial mitogens has shown promise as novel anti-neoplastic strategies, which are beginning to undergo clinical therapeutic trials in humans. The analysis of tumor angiogenic activity in patients undergoing potentially curative resection was first shown by Weidner and co-workers to be an independent measure of clinical outcome in breast cancer (106). Subsequent studies have generally supported the prognostic value of tumor angiogenesis in both node-negative and node-positive breast cancer, as well as in cancers of the cervix, kidney, bladder, ovary and stomach (107-113). A variety of growth factors have been found to be capable of inducing endothelial cell proliferation, migration or tube formation in vitro, and to act as direct angiogenesis factors in corneal neovascularization assays (105). Recent interest has focused on the possible contribution of vascular endothelial growth factor (VEGF) to tumor angiogenesis.On a molar basis VEGF is the most potent known endothelial mitogen and is widely expressed in solid tumors (reviewed in 114). In colorectal cancer, expression of VEGF and its receptors are up-regulated in comparison to normal bowel and inhibition of VEGF activity has a potent antitumor effect in animal models of liver metastasis (115).

In colorectal cancer, only a limited number of studies have examined the relationship between measures of tumor angiogenesis and prognosis, and no consensus has yet emerged. Two studies of colon cancer and one in rectal cancer, employing anti-von Willebrand factor staining of tumor vessels, have reported a statistically significant association between microvascular density (MVD) and either risk of metastasis or survival (116,117). However, Bossi et al. could demonstrate no independent prognostic value to microvessel quantitation using anti-CD31 antibodies to stain tumor vessels (118). Tomisaki et al. recently found that anti-vWF quantitation that MVD was associated with hepatic metastasis but not with overall survival in a heterogeneous group of patients with Dukes A, B or C cancers (119). Takahashi et al. have further identified a relationship between vessel counts, VEGF expression and p53 abnormalities in a group of 93 patients with node-negative colon cancer and have found that VEGF expression correlates with clinical outcome in these patients (120). Monson and co-workers reported that pre-operative serum VEGF levels strongly predicted disease at presentation (121). However, the predictive value of microvascular density or VEGF expression and clinical behavior in colorectal cancer has not been consistently observed (118,122). We propose to undertake the first prospective analysis of tumor angiogenesis in stage II and stage III colon cancer by measuring both MVD and VEGF expression by semiquantitative immunohistochemistry.

Anticipated Results: We expect that patients with tumors with a high microvessel density or high VEGF protein expression will have a higher risk of tumor recurrence and lower overall survival. The essay for VEGF by immunohistochemistry is semi-quantitative at best. A preferable approach would be to use a quantitative ELISA, as has been applied to breast cancer specimens recently (123, 124), revealing a strong independent association between tumor VEGF levels and survival in node-negative patients. However, only paraffin-embedded formalin fixed tissue will be available for the present study, mandating an immunohistochemistry assay. Based upon the results of VEGF neutralization studies in animals (115), we expect that in colon cancer VEGF acts as the predominant tumor angiogenesis factor. Consequently, we expect a strong correlation between MVD and VEGF protein immunohistochemistry. Because wild-type p53 has been shown to up-regulate the expression of angiogenic inhibitors and down-regulate the expression of angiogenic stimulators (125-127), we will test for an association between p53 over-expression by immunohistochemistry and both microvascular density and VEGF expression in these cancers.

5. Markers of Chemosensitivity: Thymidylate Synthase and Topoisomerase 1

Thymidylate synthase (TS) is an essential enzyme needed for DNA synthesis in S-phase of the cell cycle and is the target enzyme of 5-fluorouracil (5-FU), the most important chemotherapeutic agent in the treatment of colorectal cancer. The clinical importance of TS protein has been suggested by studies demonstrating that intrinsic levels of TS correlate with resistance to 5-FU in experimental tumors and in the clinical setting (128,129). Studies of patients with colon and gastric cancer have indicated that intratumoral TS gene expression is a major determinant of resistance to 5-FU (130,132). Monoclonal antibodies to human TS have been recently developed that have the sensitivity and specificity to detect and quantitate TS enzyme in the cytosol from human cells and tumor tissues (133). In collaboration with Dr. Patrick Johnston of the NCI-Navy Medical Oncology Branch, we were able to show that both TS protein and TS gene expression are significantly associated with response to 5-FU based chemotherapy (134). A recent retrospective study by Johnston et al. in 249 patients with rectal carcinoma has indicated that TS protein expression determined by immunohistochemistry may be a powerful prognosticator of disease free survival (DFS) and overall survival (135). Moreover, TS expression was independent of Dukes' stage and other standard clinico-pathological criteria. It has been suggested that quantitation of a chemo-therapeutic target such as TS may not only allow prediction of risk of recurrence but may help identify patients who would benefit from adjuvant chemotherapy within each Dukes' stage (136).

Understanding the role of TS as a predictor of survival and fluoropyrimidine responsiveness might lead to improved therapeutic strategies in patients with colon cancer (136). Preliminary data of intratumoral TS protein levels in patients with stage II colon cancer suggest that high TS protein expression level is strongly associated with tumor recurrence, indicating that TS expression may be an independent prognostic factor for recurrence and clinical outcome in patients with colon cancer (see below).

p53 and its relation to TS: The role of p53 in cell cycle regulation suggests that it might be a prime candidate in controlling TS expression. Our in vitro data indicate a possible association between TS expression and p53 status. Transfection of wt p53 into p53 null HL 60 cells lead to decreased TS gene expression levels indicating an association between p53 and TS (137). In vivo data demonstrated that tumors with mutant p53 had significantly higher TS gene expression than tumors with wt p53 in patients with advanced colorectal cancer. Furthermore, in patients with stage II colon cancer a significant correlation was found between high TS protein expression and p53 nuclear overexpression (see Tables A-4a and A-4b).

TABLE A-4a

Association of TS Expression And p53 Overexpression with Recurrence

Variable	Category	# Pts.	# of Recurrences	Prob. of Recurring at 4 Yrs.1	Relative Risk2	p-value3
TS Staining	High (≥2)	16	8	0.59±0.14	3.10	0.027
	Low (≤1)	29	6	0.21±0.08		
p53 Over-Expression	Yes	18	10	0.63±0.12	5.05	0.002
	No	27	4	0.15±0.07		
p53 and TS Together	High TS & p53 Over.	13	8	0.75±0.14	4.71	0.001
	Low TS or no p53 Over-Exp.	32	6	0.19±0.07		

TABLE A-4b

Association of TS Expression and p53 Overexpression with Survival

Variable	Category	# Pts.	# of Deaths	Prob. of Surviving at 4 Yrs.[1]	Relative Risk2	p-value[3]
TS Staining	High (≥2)	16	9	0.67±0.12	5.36	0.004
	Low (≤1)	29	4	0.86±0.07		
p53 Over-Expression	Yes	18	10	0.63±0.12	7.03	<0.001
	No	27	3	0.89±0.06		
p53 and TS Together	High TS & p53 Over	13	9	0.59±0.14	7.35	<0.001
	Low TS or no p53 Over-Exp.	32	4	0.87±0.06		

1 Estimated probabilities of recurring or dying are based on the Kaplan-Meier estimator; standard errors are based on Greenwood's formula.

2 Relative risk of failing (recurring or dying) with high TS or p53 overexpression, based on Pike method.

3 Two-sided p-values are based on the logrank test.

Of the 16 tumors which demonstrated high TS protein expression, thirteen also had p53 overexpression in the same tumor sections. We found a strong association between the p53 nuclear overexpression and the cytoplasmatic TS staining (p<0.0001) (see Table 4). These data confirm earlier findings in 37 patients with advanced colorectal cancer that mutant p53 was associated with higher TS gene expression (p=0.035)(138). In these tumors, p53 status was determined by cDNA cycle sequencing and TS gene expression levels by quantitative RT-PCR. Of the 13 tumors with high TS expression and p53 nuclear overexpression, 11 tumor had recurrence (85%) with a relative risk of 5.96. These data indicate that p53 and TS expression might be associated and not independent risk factors.

Topo 1: Topoisomerase I inhibitors have cytotoxic mechanisms which depend on DNA damage detection, DNA repair, cell cycle arrest, and cell death by apoptosis. Cancer cells often have defects within these control systems, and these defects may confer selective sensitivity of drugs for therapy based on molecular determinants of individual tumors. Based on preclinical and clinical data, the potential molecular determinants of response to CPT-11 are: 1) topoisomerase 1; 2) genes involved in apoptosis; and 3) DNA repair.

Topoisomerase 1 expression: A decrease of Topoisomerase 1 content in CPT-resistant cells has been demonstrated in various cell lines indicating that Topoisomerase 1 mRNA levels may contribute to their resistance. In CPT-11 resistant cells a marked decrease of Topoisomerase 1 protein was observed, indicating that the decreased protein content of Topoisomerase 1 may cause the decreased activity of Topoisomerase 1 and the decreased sensitivity to Topoisomerase 1 inhibitors. Jansen et al. observed a positive relationship between the DNA topoisomerase-1 activity and the cellular sensitivity to carboxylesterase-activated CPT-11 (r =0.75, p <0.1) as well as to SN-38 (r =0.89, p <0.05). The higher topoisomerase-1 activity in COLO 320 cells and tumors when compared with that in WiDr cells and tumors reflected the

differences in sensitivity to the drug(s). In conclusion, the DNA topoisomerase-1 activity was the best determinant for CPT-11/SN-38 sensitivity in this panel of unselected human colon-cancer cell lines (139).

Levels of topoisomerase 1 expression have been shown to vary widely between and within tumor types but the basis for this is poorly understood. Preliminary clinical data from patients with colorectal cancer suggest that topoisomerase 1 gene expression may be a predictor of clinical outcome (140). High topoisomerase 1 gene expression levels were associated with response to CPT-11; thus high expression levels of topoisomerase 1 may be predictive of responsiveness to CPT-11. Interestingly, the topoisomerase 1 gene expression levels were independent from the TS expression level indicating that response to CPT-11 is possible despite increased TS expression levels which is associated with resistance to 5-FU. There have been no prospective studies to date that have attempted to correlate the level of Topoisomerase 1 expression and response to treatment with irinotecan or survival in colorectal cancer.

We do not as yet have a reliable assay for Topoisomerase 1 suitable for use in archival, formalin fixed specimens of colon cancer. When an assay for Topo 1 is developed and tested for sensitivity, specificity and reliability in fixed tissue, an analysis of the relationship between levels of Topoisomerase 1 expression and outcome in the present study will be undertaken.

III. STUDY DESIGN AND METHODS

1. Specific Aim

To determine whether tissue-based and molecular markers (thymidylate synthase, p53, p21, p27, VEGF, MVD, 18q LOH, MSI) are independent covariates in a multivariate analysis with histologic and standard clinicopathologic variables in patients prospectively accrued in a stage III colon carcinoma adjuvant therapy clinical trial. Analyses will be stratified by treatment randomization. These markers and their prognostic value will be assessed in patients treated in each therapeutic arm of the clinical study.

2. Experimental Design

We propose to test whether the expression of markers in primary carcinomas is associated with survival and response to therapy in an adjuvant chemotherapy trial in stage III primary colon carcinoma. Approximately 1260 patients will be randomized to receive 5-FU plus leucovorin alone or in combination with CPT-11 following potentially curative surgery. Most of the markers to be studied have been shown in individual retrospective trials to be significantly associated with overall or disease-free survival. We will analyze intratumoral protein markers by immunohistochemistry and 18q LOH and MSI by PCR-based analysis of formalin-fixed, paraffin-embedded tissues, and compare marker expression to the presence of standard clinical and pathological factors. Samples will be coded and analyzed independently by two investigators whose scores will be averaged for both the intracellular distribution and the frequency of cells that express markers. The same coding system will be used to assure that pathologic staging is correct. Inter-rater agreement will be assessed as described in Section V. The potential interaction of markers on clinical outcome will also be assessed.

3. Pathologic Assessment of Prospectively Accrued Tissues

Tissues for clinical diagnosis will be fixed in formalin, embedded in paraffin, and processed for routine staining with hematoxylin and eosin by the Pathology Coordinating Laboratory of CALGB. We will request 1-2 blocks for each study subject from the primary tumor. We will also request a block containing non-neoplastic tissue (normal colon or uninvolved lymph node). Extra sections of each block will be cut and will be archived by CALGB in anticipation of additional marker studies that may require such tissue in the future. Pathologic staging is confirmed according to TNM staging (7). Histological evaluation of tissues will be performed independently of the marker analysis. After ensuring that sections contain predominantly tumor (>70% neoplastic) cells, the number of slides indicated below will be sent to the individual co-investigators for marker analysis. We anticipate sufficient material to perform all the analyses described. In the event that tissue availability is limited, the analyses will be undertaken under the following priority scheme: TS, p53, 18q LOH, MSI, p27, p21, VEGF, MVD, Topo 1.

IV. METHODS

Each marker analysis will be performed in a coded manner independently of the standard histopathologic assessment by two investigators at each participating site. Marker expression will be evaluated for intensity of staining (weak, moderate, or strong) and percent of cells positive, and will be recorded as continuous variables. The data will be converted to high or low, positive or negative, defined as follows (based upon preliminary studies sited in sections II and III, above): TS, high=mod. or strong staining in >25%; p53, p21, positive=mod. or strong nuclear staining in >10%; p27, high=mod. or strong nuclear staining in >50%; VEGF, high=mod. or strong in >50%; MVD, high=greater than 28 vessels per 100X field. All IHC markers will thus have a binomial outcome.

A. Immunohistochemistry will be performed on deparaffinized, rehydrated 4-6 micron sections of formalin-fixed tissue. Sections from positive and negative control tumors (which have been analyzed previously for either, p53, p21, p27, TS, or VEGF and found to be strongly positive or completely negative) and controls in the absence of primary antibody will be utilized routinely. In certain cases (VEGF, p53,), microwave antigen retrieval methods will be used. Details of the methods for TS, CD31, VEGF and p27 staining have been published (133,141,116,76). Methods for analysis of p21 and p53 are given below.

B. MVD and p53 and p21, VEGF Immunohistochemistry: (3 slides for each parameter) The tumor block will be cut in consecutive sections 5um thick. The sections will be deparaffinized in xylene and rehydrated in a series of graded alcohols. The sections will be treated with 0.3% hydrogen peroxide solution to exhaust endogenous peroxidase activity. After microwave treatment, the sections will be preincubated in 1% bovine serum albumin in phosphate-buffered saline, and monoclonal antibodies will be applied to the sections: D-07 (Dako; code M7001), which reacts with wt type and mutant forms of human p53 protein, dilution 1:50, a mouse monoclonal antibody (Pharmingen 15441A) which reacts with the p21 protein at a dilution of 1:50 or a polyclonal antisera directed against VEGF 165 (Santa Cruz Antibodies). Biotinylated rabbit anti-mouse antibody (DAKO; code E354) will be used as secondary antibody, and the immunoreaction will be visualized by avidin-biotin complex. After a final rinse in PBS, the tissues will be incubated with diaminobenzidine DAB substrate (0.02% DAB, 0.005% hydrogen peroxidase) for 15 minutes and then will be counterstained in hematoxylin 100% (GILL 11 formula) and mounted with glass coverslips using permount. A positive and negative control (tumors with known p53 status) will be included in each batch of staining. For p53, the extent of the nuclear reactivity will be classified: -: no reactivity; +/-: few focally positive cells; +: heterogeneous nuclear reactivity. Only tumors that show >10% nuclear reactivity

will be considered as demonstrating p53 alterations and tumors with >10% nuclear expression of p21/WAF1/CIP1 will be considered as positive expression. MVD will be determined in the leading edge of the tumor in an area of apparent highest vessel density following anti-CD31 staining (141). The fraction of cells demonstrating cytoplasmic staining for VEGF will be measured and recorded. Tumors with >10% VEGF staining cells will be considered positive.

C. p27 Immunohistochemistry: (3 slides) Immunoperoxidase staining using rabbit polyclonal antisera will be done by a standard 3-step technique using swine anti-rabbit immunoglobulin as the bridging antisera and peroxidase-anti-peroxidase (PAP) followed by antigen localization with diaminobenzidene (DAB) as previously described (76). Slides will be counterstained with hematoxylin or methyl green.

D. Thymidylate Synthase (TS): (3 slides) Methods for thymidylate synthase quantitation have been published previously (133,138).

E. DCC: LOH analysis for 18q will be measured as follows: Six (6) 10-micron thick sections per patient will be cut from formalin-fixed paraffin-embedded tissues (also 5-micron thick sections on polylysine coated slides, for future IH studies). One slide will be stained with hematoxylin/eosinfor histopathologicalexamination.The slides will be stained with hematoxylin and eosin, dehydrated in graded ethanol, and then dried without a cover glass. Regions containing at least 70 percent neoplastic cells will be inked with a black marker (Sharpie, Sanford Corp., Bellwood, IL) under a dissection microscope. The black marking ink will increase the density of the tissue and keep it at the bottom of the tube after centrifugation. Tissues from 2 to 6 slides, each containing a blackened region of tumor 0.2 to 1 cm^2 in area will be scraped off with a razor blade and transferred to a 1.5 ml Microfuge tube. Non-neoplastic tissue from the same slide or from sections of adjacent normal colon will then be marked and placed in another Microfuge tube. The collected tissue samples will be deparaffinized in 400 ml of xylene for 15 minutes and pelleted by centrifugation at 10,000 x g for 2 minutes. After the xylene is removed by pipette, the tissues will be heated at 58°C for 15 minutes to remove the remaining xylene and incubated overnight at 58°C in a buffer containing 0.5M TRIS (pH 8.9), 210 mM EDTA, 10 mM sodium chloride, 0.5 mg of proteinase K per milliliter, and 1 percent sodium dodecyl sulfate. The samples will be boiled in a water bath for 10 minutes at 100°C, cooled to room temperature, and then extracted twice with an equal volume of phenol and chloroform, as previously described. DNA will be precipitated with ethanol and dissolved in 30 ml of buffer containing 3 mM TRIS (pH 7.5) and 0.3 mM EDTA.

Oligonucleotide primers for microsatellite markers from the long arm of chromosome 18 will be designed on the basis of published sequences. The following dinucleotide-repeat markers and primers will be used in the prospective study:

D18S55, 5'GGGAAGTCAAATGCAAAATC3' and 5'AGCTTCTGAGTAATCTTATGCTGTG3; S18S58, 5'GCTCCCGGCTGGTTTT3' and 5'GCAGGAAATCGCAGGAACTT3' and 5'GCAGGAAATCGCAGGAACTT3'; D18S61, 5'ATATTTTGAAACTCAGGAGCAT3'; D18S64, 5'AACTAGAGACAGGCAGAA3' and 5'ATCAGGAAATCGGCACTG3'; and D18S69, 5'CTCTTTCTCTGACTCTGACC3' and 5'GACTTTCTAAGTTCTTGCCAG3'

PCR-based dinucleotide-repeat assays will be carried out in 96-well plates for 30 cycles; each cycle will be carried out at 95°C for 30 seconds, 50°C for 1 minute, and 70°C for 1 minute, with primers end-labeled with P-labeled ATP to a specific activity of more than 10^8 cpm per microgram of DNA and under the PCR conditions previously described. Two volumes of stop buffer (95 percent formamide, 20 mM sodium hydroxide, and 0.05 percent bromophenol blue and xylene cyanate) will be added at the end of the amplification, and the samples will be loaded onto 7 percent polyacrylamide gels containing 32 percent formamide and 5.6 M urea.

Determination of Chromosome 18q Status: We will define chromosome 18q loss as the complete or partial loss of the long arm of chromosome 18. Loss of a chromosome 18q allele in a tumor will be considered to be present when the PCR assay of adjacent non-neoplastic tissue showed heterozygosity of the microsatellite markers on the long arm of chromosome 18, and the relative intensity of the two alleles in the tumor DNA differed from the relative intensity in the non-neoplastic tissue DNA by a factor of at least 1.5. When the loss of the allele was not obvious on visual inspection, the intensities of the bands were quantitated with a PhosphorImager (Molecular Dynamics, Sunnyvale, Calif.).

F. Microsatellite Instability: DNA will be extracted separately from slides of tumor-containing and non-tumor-containing tissue. Primers for PCR amplification of the variable length repeat segments recognized by probes D2S123, D2S119, D3S1029, D10S197, AP3, BAT 25, BAT 26, D5S346, D17S250, and D1S158 will be obtained from Perkin Elmer, Inc. These primers will be tagged with a fluorescent marker to allow analysis of the PCR products using the Applied Biosystems 377 Automated Sequencer with GeneScan and Genotyper Software. Standard PCR reaction of 35 cycles will be performed for each primer pair. The products of PCR will be prescreened on an agarose gel to insure amplification of target DNA and the absence of any non-specific PCR products. The products will then be denatured, and run on a non-denaturing high-resolution 10% polyacrylamide gel. RER positives are cases in which there are differences in the band pattern between the DNAs from tumor and normal tissue from the same person. The alterations generally involve additional CA-repeat bands, seen as band shifting on the gels. Other changes, such as the loss of one or more bands from tumor when compared with normal tissue from the same person, known as loss of CA repeats, may also suggest mutations in mismatch repair genes. All positives will be confirmed at least twice.

For analysis of results in this study, we will define RER+ according the NCI Workshop Recommendations, i.e., for the following 5 loci: BAT 25, BAT 26, D5S346, D2S123, and D17S250, >2 loci exhibiting instability will indicate MSI-H or RER+ tumors. Instability will be defined as the presence of a band shift. Markers showing apparent LOH only (without the presence of novel fragments) will be scored as negative. Although they are not included in the international guidelines for evaluation of MSI/RER in colorectal cancer, the remaining 5 loci described in the study are of interest to our investigators because they were used in previous analyses of colorectal tumors. These markers will be assessed as laboratory resources permit, and will not be used to determine RER+ versus RER- for purposes of this study.

V. STATISTICAL CONSIDERATIONS

1. Tracking Specimens and Data Collection

Labeled samples will be sent from CALGB's PCO to each laboratory. Laboratories will be responsible for reporting the receipt of each sample and the corresponding results on a monthly basis. Data will be accepted electronically at the CALGB Data Management Center according to each laboratory's predetermined format. Software will be written to allow uploading of this data to the CALGB Database.

2 Assessment of Inter-Rater Agreement

Raters in each laboratory will independently qualify marker expression according to a well-defined scale containing K levels describing the results for each marker. There will be two raters for each marker. A 25% random sample will be taken from the first 200 tissue samples in order to estimate inter-rater agreement. A kappa measure of agreement proposed by Kraemer will be used to estimate the agreement between raters for each marker. The jack-knife method will be used to estimate the agreement measure, k, and its variance. An approximate lower confidence bound for k without the assumption of no agreement can be obtained using this method (142). Thus, the existence of moderate to strong agreement can be determined. With 50 measurements, an agreement of 0.5 between raters can be detected with approximately 80% power. If agreement is deemed unacceptablewe will institute further training and continue to monitor agreement in the next 200 samples.

Similarly, immunohistochemistry at each laboratory will be monitored by the CALGB PCO. If agreement is found to be unacceptable classification criteria will be reviewed by both investigators. Agreement will continue to be monitored in additional patients as necessary until agreement is determined to be acceptable. This process can be repeated as required throughout the study.

3. Primary Objectives

Eighteen hypotheses will be tested using the proportional hazards model. The relationship between each of nine markers, TS, p53, p21, p27, VEGF, MVD, 18q LOH, MSI, Topo1 and time to recurrence and survival will be assessed controlling for other predictive baseline characteristics. The familywise error rate will be controlled for independently for each outcome.

4. Survival Analysis Methodology

The Cox proportional hazards model (143) will be used to determine the association between each marker (TS, p53, p21, p27, VEGF, MVD, 18qLOH, MSI, Topo1) and time to recurrence and survival controlling for other baseline factors such as age, gender, treatment, primary tumor site, degree of differentiation. Time to recurrence will be measured from time of resection until documented disease progression. Preliminary data suggest that very large differences exist in time to recurrence with respect to the presence or absence of certain markers (Specifically, TS, p53, p27, 18q LOH, MSI) (Preliminary Studies.).

Table A-5 (below) provides detectable differences and associated power by varying numbers of samples studied for three markers under investigation. In all examples the assumed prevalence and median survival and time to recurrence for the higher risk group are based on preliminary data in stage II/II-III colon cancer patients as described in Section II. Power estimates are based on a one-sided logrank test at significance level 0.006. Exponential distributions are assumed for survival and time to recurrence with 450 patients accrued over 2.8 years and followed an additional 3 years. The examples cover a range of possible outcomes. For markers with prevalence

from 0 to 30% or 70% to 100% larger samples are required to detect the same hazard ratio than for markers with prevalence in the range of 30% to 70%. (144,145)

TABLE A-5

Table of Detectable Differences and Associated Power by Numbers of Samples Studied for Low TS, p27 Overexpression, p53 Overexpression

Marker (prevalence)	Detectable Hazard Ratio	Median* (years)	No. Samples (% total)	Power
low TS (64%)	1.39	1.78	945 (75)	0.93
	1.50		945 (75)	0.99
	1.50		630 (50)	0.92
	1.70		315 (25)	0.80
p27 overexpression (10%)	1.70	5.75	945 (75)	0.62
	2.00		945 (75)	0.88
	2.00		630 (50)	0.68
	2.20		630 (50)	0.80
p53 overexpression (40%)	1.50	6.00	945 (75)	0.79
	1.80		630 (50)	0.91
	2.15		315 (25)	0.80

* Median is median time to recurrence in years for low TS and survival in years for p27 and p53 overexpression.

Graphical techniques and formal tests such as those proposed by Cox, Schoenfeld and Anderson will be used to assess validity of assumptions and goodness of fit of the proportional hazards model (146-148). At the time of analysis, we will investigate potential differences in disease free interval and survival outcomes between patients with missing specimens and those who provided specimens to determine if a bias is present. The role of other factors potentially associated with outcome such as TNM stage, primary tumor site, degree of differentiation and presence of nodal metastases will be considered. (Associations between prognostic markers and stage-related and grade-related clinical variables will be investigated in an exploratory analysis. See below.) If there is an apparent bias, we will analyze patients with and without missing specimens separately. Once the "best" model with respect to baseline factors is determined, each of the nine markers will be tested individually for association with time to recurrence and survival. In each case, the p-value associated with the change in the log likelihood ratio will be used to test if the addition of the new marker adds significantly to the overall goodness of fit according to the procedure of Hochberg (149). This procedure will be used to control the familywise error rate for testing the nine hypotheses for each outcome, time to recurrence and survival. An overall significance level of alpha=0.05 will be used. The p-values associated with each of the nine tests will be ordered and compared sequentially with the following values beginning with the largest p-value, 0.05, 0.025, 0.016, 0.0125, 0.010, 0.0083, 0.0071, 0.0062, 0.0056. If a given p-value is found to be less than its corresponding level, the associated hypothesis and all remaining hypotheses will be rejected.

5. Multiple Comparisons

Controlling the familywise error rate will be a concern. An attempt to control this error rate will be made for the primary objectives, i.e., assessing the relationships

between each marker and time to recurrence and survival. In the exploratory data analysis, p-values will not be emphasized.

6. Exploratory Data Analysis

An exploratory analysis will investigate: 1) the validity of the normality assumption for each marker; 2) the potential inter-relationships among markers; 3) whether interactions among the expression of tumor markers identify subsets of patients with significantly altered outcome.

Graphical methods such as box plots, stem and leaf plots, histograms and q-q plots will be used to describe the probability distribution of each marker. When appropriate these will be based on continuous outcome measures. Transformations may be used to achieve normality. Joint distributions will also be considered. For example, preliminary data has suggested that p53 and TS expression are highly associated. With a sample size of 300 specimens a correlation of 0.2 could be detected with an approximate power of 0.80 testing at significance level, alpha=0.006.

In general, methods of determining the relationships among markers will be guided by the underlying distributions. Categorical and nonparametric methods will be used as indicated. Graphical methods will also be used to illustrate the relationships between markers. If appropriate, i.e., the assumption of multivariate normality appears to be justified, methods such as principal components and regression will be used to describe relationships among markers. Principal components will be used to identify specific markers or groups of markers that may describe underlying factors and regression analysis will be used to determine the linear relationships between markers.

Non-parametric exploratory methods will include the use of classification and regression trees (CART). The primary goal in using CART would be to test whether markers can be sequenced into a prognostic tree, with an aim at reducing the overall number of marker evaluations necessary on individual patients to obtain a good predictive model. At the same time, CART has the potential to reveal complex marker interactions and, using data available on patients without sample submissions, may allow for the evaluation of the prognostic ability of demographic and/or other clinical data as surrogate measures for tissue markers.

Associations between prognostic markers and stage-related and grade-related clinical variables will also be investigated. Once these associations and the inter-relationships among markers have been identified, we will investigate their prognostic significance with respect to outcome.

VI. TISSUE MICROARRAY ANALYSIS (TMA) PRODUCTION

1. Background

The first concept of a multicore tissue block was described in 1986 by Battifora to test new antibodies by IHC. This concept has been further refined through Tissue Microarray Analysis (TMA) described by Kononen et al in 1998 which is a high-throughput technique that enables the analysis of hundreds of specimens, by arranging up to 1,000 samples of 0.6 mm tissue core biopsy specimens taken from donor blocks into a single paraffin block. Sections are then cut from TMA blocks using standard microtomes. This technique is optimally suited for large scale molecular profiling projects and can be applied to all kinds of in situ analyses.

Several different TMAs may be manufactured and employed in a broad range such as determining the prevalence of genetic alterations in tumors, progression studies to find associations between gene or protein alterations and different tumor stages and prognostic studies in tumor samples with available clinical follow-up data. Thus, it is ideal in a study setting where there is a large collection of well-characterized

tissues with attached clinical data, as it is in our study, to analyze promising molecular markers for their prognostic significance.

Validation studies have been done to evaluate the representativeness of small disks (0.6 mm in diameter) taken from the original tissue by comparing immunhistochemistry findings on TMAs versus the corresponding standard whole tissue sections. The vast majority have shown a concordance rate of 98-99% of the results. The number of cores required to adequately represent the expression of a marker has also been evaluated. In general, these studies found that two or three core samples provided more representative information than a single sample and that adding more than four or five samples would not lead to a massive improvement in the concordance level [137].

2. Rationale

Large scale molecular profiling of cancer specimens is slow and tedious when traditional methods of molecular pathology are used. Also, cutting traditional tissue sections for a multitude of molecular tests for genes of interest would easily exhaust valuable tissue resources. The use of tissue microarray technology offers to overcome these impediments by taking up to 1000 different minute tissue samples to be put into one glass slide and simultaneously analyzed by in situ methods such as immunhistochemistry.As this method enables the analysis of hundreds of cases with the amount of reagents usually used for one case, this technique will be both cost-efficient and time-saving. Published studies claim that this also offers an unprecedented degree of standardization as the tissue samples are subjected to the same experimental conditions and batches of reagents [137]. Other advantages would be that the interpretation of small areas of 0.6 mm is easier since the heterogeneity and staining artifacts at borders of whole sections are eliminated. Punches of 0.6 mm also minimize tissue damage on these donor blocks so that they can still be used for other studies [138].

TMAs will optimize the use of the collected histopathologically well-defined tissue samples in this study with attached clinical data, for current and future applications. Currently, there is a limited availability of TMA sections from various sources both commercial and academic especially with attached clinical follow-up, which is insufficient to cover demands for collaborations.However, the number of institutions manufacturing TMAs is increasing and it is expected that virtually all institutions dealing with tissue analyses will be using TMAs in the future.

3. Methods and Procedures

One block representative for a tumor specimen per case will be used. Under the microscope, areas of interest from the H&E whole section of the donor block, non-necrotic areas rich in tumoral glands will be marked accurately. The selection of large tumoral areas in the donor block is important to avoid nonevaluable disks. A map of the receiver block will be prepared with coordinates for each sample to correctly identify the tumors. A tissue microarrayer will be used to punch out 3 cores of 0.6 mm diameter from each donor block and positioned in the recipient paraffin array block, in smaller holes of 0.4 mm for best adhesion of the samples to the array block. Additional cores greater than 3 will be determined by pathology review. Approximately 432 cores make up one block, so therefore 144 patients or cases can be placed per TMA block. The array blocks will then be incubated 30 minutes at 37 degrees centigrade to improve adhesion between cores and paraffin of the recipient block. These blocks will be cut at room temperature with a standard microtome and processed according to the study protocol.

VII. REFERENCES

1. Wingo PA, Ries LA, Rosenberg HM, et al. Cancer incidence and mortality, 1973-1995: a report card for the U.S. Cancer 82:1197-207, 1998.

2. Muto T, Bussey HJR, and Morson BC. The evolution of cancer of the colon and rectum. Cancer 36:2251-2276, 1975

3. O'Connell MJ, Laurie JA, Kahn M, et al. Prospectively randomized trial of postoperative adjuvant chemotherapy in patients with high-risk colon cancer. J. Clin. Oncol. 16:295-300, 1998.

4. Kemeny N, Saltz L, and Cohen A. Adjuvant therapy of colorectal cancer. Surg. Onc. Clin. North Am. 6:599-722, 1997.

5. Jass JR, Atkin WS, Cuzick J, et al. The grading of rectal cancer: historical perspectives and a multivariate analysis of 447 cases. Histopathology 10:437-59, 1986.

6. Dukes CE. The classification of cancer of the rectum. J. Pathol. Bact. 35:323-332, 1932.

7. TNM: Classification of Malignant Tumours. Eds. Hermanek P. and Sobin LH. Fourth Edition. Springer-Verlag. Berlin. pp. 47-50, 1987.

8. Gunderson LL, Sosin H. Areas of failure found at reoperation(second or symptomatic look.) following "curative surgery" for adenocarcinoma of the rectum. Cancer 34:1278-92, 1974.

9. Astler, VB. and Coller, FA. Prognostic significance of direct extension of carcinoma of colon and rectum. Ann. Surg. 139:846-852, 1954.

10. Jass JR. The pathological grading and staging of rectal cancer. Scand J Gastroenterol Suppl. 149:21-38, 1988.

11. Bjerkeset T, Morild I, Mork S, Soreide O. Tumor characteristics in colorectal cancer and their relationship to treatment and prognosis. Diseases of the Colon & Rectum. 30(12): 934-8, 1987.

12. Halvorsen TB, Seim E. Influence of mucinous components on survival in colorectal adenocarcinomas: a multivariate analysis. J Clin Pathol 41:1068-1072, 1988

13. Stahle E, Glimelius B, Bergstrom R, Pahlman L. Preoperative clinical and pathological variables in prognostic evaluation of patients with rectal cancer. A prospective study of 327 consecutive patients. Acta Chirurgica Scandinavica. 154:231-9, 1988.

14. Fisher ER, Robinsky B, Sass R, Fisher B. Relative prognostic value of the Dukes and the Jass systems in rectal cancer. Findings from the National Surgical Adjuvant Breast and Bowel Projects (Protocol R-01). Dis. Colon Rect. 32:944-9, 1989.

15. Tomoda H, Kakeji Y, Furusawa M. Prognostic significance of flow cytometric analysis of DNA content in colorectal cancer: a prospective study. J Surg Oncol. 53:144-8, 1993.

16. Schillaci A, Tirindelli DD, Ferri M, et al. Flow cytometric analysis in colorectal carcinoma: prognostic significance of cellular DNA content. Int J Colorectal Dis. 5:223-7, 1990.

17. Zoras O, Curti G, Cooke TG, Vlachonikolis IG, Forster G, McArdie CS and Stanton PD. Prognostic value of ploidy of primary tunour and nodal secondaries in colorectal cancers. Surg. Oncol 3(6): 345-9, 1994.

18. Yamazoe Y, Maetani S, Nishikawa T, Onodera H, Tobe T and Imamura M. The prognostic role of the DNA ploidy pattern in colorectal cancer analysis using paraffin-embedded tissue by an improved method. Surg. Today 24(1): 30-6, 1994.

19. Silvestrini R, D'Agnano I, Faranda A, Costa A, Zupi G, Cosimelli M, Quaglliuolo V, Giannarelli D, Gennari L and Cavaliere R. Flow cytometric analysis of ploidy in colorectal cancer: a multicentric experience. Br. J. Cancer 67(5): 1042-6, 1993.

20. Verspaget HW, Sier CFM, Ganesh S, Griffioen G and Lamers CBHW. Prognostic value of plasminogen activators and their inhibitors in colorectal cancer. Eur J Cancer, 31A(7/8): 1105-9, 1995.

21. Murray GI, Duncan ME, O'Neil P, Melvin WT, and Fothergill JE. Matrix metalloproteinase-1 is associated with poor prognosis in colorectal cancer. Nature Med 2(4): 461-2, 1996.

22. Herrlich P, Pals S and Ponta H. CD44 in colon cancer. Eur J Cancer 31A: 1110-2, 1995.

23. Nakayama T, Watanabe M, Katsumata T, Teramoto T and Kitajima M. Expression of sialyl Lewis(a) as a new prognostic factor for patients with advanced colorectal carcinoma. Cancer 75(8): 2051-6, 1995.

24. Fielding LP and Pettigrew N: College of American Pathologists Conference XXVI on clinical relevance of prognostic markers in solid tumors. Report of the Colorectal Cancer Working Group. Arch Path and Lab Med 119(12): 1115-21, 1995.

25. Harpole DH, Herndon JE, Wolfe WG, Iglehart JD and Marks JR. A prognostic model of recurrence and death in stage I non-small lung cancer utilizing presentation, histopathology and oncoprotein expression. Cancer Res 55: 51-56, 1995.

26. Isobe T, Hiyama K, Yoshida Y, Fujiwara Y, Yamajidao M. Prognostic significance of p53 and ras gene abnormalitiesin lung adenocarcinomapatients with stage I disease after curative resection. Jpn J Cancer Res 85:1240-1246, 1994.

27. Allred DG, Clark GM, Elledge R, Fuqua SAW, Brown RW, Chamness GC, Osborne CK, McGuire WL. Association of p53 protein expression with tumor cell proliferationrate and clinical outcome in node-negative breast cancer. J Natl Cancer Inst 85:200-206, 1993.

28. Silvestrini R, Benini E, Daidone MG, Veneroni S, Borachi P, Cappelletti V, Di Fronzo G, Veronesi U. p53 as an independent prognostic marker in lymph node-negative breast cancer patients. J Natl Cancer Inst 85:965-970, 1993.

29. Bergh J, Norberg T, Sjogren S, Lindgren A and Holmberg L. Complete sequencing of the p53 gene provides prognostic information in breast cancer patients, particularly in relation to adjuvant systemic therapy and radiography. Nature Med 1(10): 1029-34, 1995.

30. Esrig D, Elmajian D, Groshen S, Freeman JA, Stein JP, Chen S-C, Nichols PW, Skinner DG, Jones PA and Cote RJ. Accumulation of nuclear p53 and tumor progression in bladder cancer. N Engl J Med 331: 1259-1264, 1994.

31. Offerhaus GJA, De Feyter EP, Cornelisse CJ, Tersmette KWF, Floyd J, Kern SE, Volgelstein B and Hamilton SR. The relationship of DNA aneuploidy to molecular genetic alterations in colorectal carcinoma. Gastroenterology 102:1612-1619, 1992.

32. Hsiao MH, Yu AL, Yeargin J, Haas M, et al. Nonhereditary p53 mutations in T-cell acute lymphoblastic leukemia are associated with the relapse phase. Blood 83:2922-2930, 1994.

33. Scott N, Sagar P, Stewart J, et al. p53 in colorectal cancer: Clinicopatholgical correlation and prognostic significance. Br J Cancer 63:317-319, 1991.

34. Remvikos Y, Tominaga O, Hammel, P, et al. Increased p53 protein content of colorectal tumours correlates with poor survival. Br J Cancer 66: 758-764, 1992.

35. Bell SM, Scott N, Cross D, et al. Prognostic value of p53 overexpression and c-ki-ras mutations in colorectal cancer. Gastroenterology 104:57-64, 1993.

36. Sun X-F, Carstensens JM, Stal O, Zhang H, Nilsson E, Sjödahl R, Nordenskjöd B. Prognostic significance of p53 expression in relation to DNA ploidy in colorectal adenocarcinoma. Virchows Archiv Pathol Anat 423:443-448, 1993.

37. Zeng Z-S, Sarkis AS, Zhang Z-F, Klimstra DS, Charytonowicz E, Guillem JG, Cordon-Cardo C, Cohen AM. p53 nuclear overexpression: An independent predictor of survival in lymph-node positive colorectal cancer patients. J Clin Oncol 10:2043-2050, 1994.

38. Bosari S, Viale G, Roncalli M, Graziani D, Borsani G, Lee AKC, Coggi G. p53 gene mutations, p53 protein accumulation and compartmentalization in colorectal adenocarcinoma. Am J Pathol 147:790-798, 1995.

39. Nathosanson SD, Linden MD, Tender P, Zarbo RJ, Jacobsen MS, Nelson LT. Relationship among p53, stage and prognosis of large bowel cancer. Dis Colon Rectum 37:527-534, 1994.

40. Auvinen A, Isola J, Visakorpi T, Koivula T, Virtanen S, Halama M. Overexpression of p53 and long-term survival in colon carcinoma. Br J Cancer 70:293-296, 1994.

41. Yamaguchi A, Nakagawara G, Kursaka Y, Nishimura G, Yonemura Y and Miyazaki I. p53 immunoreaction in endoscopic biopsy specimens of colorectal cancer and its prognostic significance. Br J Cancer 68:399-402, 1993.

42. Yamaguchi A, Kurosaka Y, Fushida S, et al. Expression of p53 protein in colorectal cancer and its relationship to short term prognosis. Cancer 70:2778-2784, 1992.

43. Cripps KJ, Purdie CA, Carder PJ, White S, Komine K, Bird CC and Wyllie AH. A study of stabilisation of p53 protein versus point mutation in colorectal carcinoma. Oncogene 9:2739-2743, 1994.

44. Mulder JW, Wielenga VJ, Pals ST, et al. p53 and CD44 as clinical markers of tumour progression in colorectal carcinogenesis. Histochemical Journal 29:439-52, 1997.

45. Ahnen DJ, Feigl P, Quan G, et al. Ki-ras mutation and p53 overexpression predict the clinical behavior of colorectal cancer: a Southwest Oncology Group study. Cancer Res 58:1149-58, 1998.

46. Lenz HJ, Hayashi K, Salonga D, et al. p53 point mutations and thymidylate synthase messenger RNA levels in disseminated colorectal cancer: an analysis of response and survival. Clin. Cancer Res. 4:1243-50, 1998.

47. Aaltonen LA, Peltomaki P, Leach FS, et al. Clues to the pathogenesis of familial colorectal cancer. Science 260:812-816, 1993.

48. Aaltonen LA, Peltomaki P, Mecklin JP, et al. Replication errors in benign and malignant tumors from hereditary nonpolyposis colorectal cancer patients. Cancer Res 54:1645-1648, 1994.

49. Thibodeau SN, Bren G, Schaid D. Microsatellite instability in cancer of the proximal colon. Science 260:816-819, 1993.

50. Ionov YM, Peinado A, Malkhosyan S, et al. Ubiquitous somatic mutations in simple repeated sequences reveal a new mechanism for colonic carcinogenesis. Nature 363:558-561, 1993

51. Lothe RE, Peltomaki P, Meling GI, et al. Genomic instability in colorectal cancer: relationship to clinicopathological variables and family history. Cancer Res 53:5849-5852, 1993

52. Samowitz WS, Slattery ML, Kerber RA. Microsatellite instability in human colonic cancer is not a useful clinical indicator of familial colorectal cancer. Gastroenterology 109:1765-1771, 1995.

53. Kim H, Jen J, Vogelstein B, Hamilton SR. Clinical and pathological characteristics of sporadic colorectal carcinomas with DNA replication errors in microsatellite sequences. Am J Path 145:148-156, 1994.

54. Aaltonen, LA, Salovaara, R, Kristo P, Canzian, F, et al. Incidence of hereditary nonpolyposis colorectal cancer and the feasibility of molecular screening for the disease. New Engl J Med 338:1481-1487, 1998.

55. Papadopoulos N, Nicolaides NC, Wei YF, et al. Mutation of a mutL homolog in hereditary colon cancer. Science 263:1625-1629, 1994.

56. Nicolaides NC, Papadopoulos N, Liu B, et al. Mutations of two PMS homologues in hereditary nonpolyposis colon cancer. Nature 371:75-80, 1994.

57. Liu B, Parsons RE, Hamilton SR, et al. hMSH2 mutations in hereditary nonpolyposis colorectal cancer kindreds. Cancer Res 54:4590-4594, 1994.

58. C. Richard Boland, personal communication

59. Liu L, Markowitz S, Gerson SL. Mismatch repair mutations override alkyltransferase in conferring resistance to temozolomide but not to 1,3-Bis(2-chloroethyl)nitrosourea. Cancer Res 56:5375-5379, 1996.

60. Lothe RA, Peltomaki P, Meling GI, Aaltonen LA, et al. Genomic instability in colorectal cancer: relationship to clinicopathological variables and family history. Cancer Res 53:5849-5852, 1993.

61. Risio M, Reato G, di Celle PF, Fizzotti M, Rossini FP, Foa R. Cancer Res 56:5470-5474, 1996.

62. Dietmaier W, Wallinger S, Bocker T, Kullmann F, Fishel R, Ruschoff J. Diagnostic microsatellite instability: definition and correlation with mismatch repair protein expression. Cancer Res 57:4749-4756, 1997.

63. Thibodeau SN, French AJ, Cunningham JM, Tester D, Burgart LJ, Roche PC, McDonnell SK, Schaid DJ, Vockley CW, Michels VV, Farr GH, O'Connell MJ. Microsatellite instability in colorectal cancer: different mutator phenotypes and the principal involvement of *hMLH1*. Cancer Res 58:1713-1718, 1998.

64. Harper JW, Adami GR, Wei N, Keyomarsi K and Elledge SJ. The p21 Cdk-interacting protein CIP1 is a potent inhibitor of G1 Cyclin-dependent-kinases.Cell 75: 805-816, 1993.

65. el-Deiry WS, Harper JW, O'Connor PM, Velculescu VE, Canman CE, Jackman J, Pietenpol JA, Burell M, Hill DE, Wang Y et al. WAF1/CIP1 is induced in p53-mediated G1 arrest and apoptosis. Cancer Res 54:1169-1174, 1994.

66. Peter M and Herskowitz I. Joining the complex: cyclin-dependent kinase inhibitory proteins and the cell cycle. Cell 79:181-184, 1994.

67. el-Deiry WS, Tokino T, Velculescu VE, Levy DB, Parsons R, Trent JM, Mercer E, Lin D, Kinzler KW and Vogelstein B. WAF1, a potential mediator of p53 tumor suppression. Cell 75: 817-825, 1993.

68. Li CY, Suardet L and Little JB. Prognostic role of WAF1/CIP1/p21 as a mediator of TGF-beta cytoinhibitory effect. J Biol Chem 270:4971-4974, 1995.

69. Sinicrope FA, Roddey G, Lemoine M, et al. Loss of p21WAF1/Cipl protein expression accompanies progression of sporadic colorectal neoplasms but not hereditary nonpolyposis colorectal cancers. Clin Cancer Res 4:1251-61, 1998.

70. Wang QM, Jones JB and Studzinski GP. Cyclin-dependent kinase inhibitor p27 as a mediator of the G1-S phase block induced by 1,25-dihydroxyvitamin D3 in HL60 cells. Cancer Res 56(2): 264-7, 1996.

71. Coats S, Flanagan WM, Nourse J and Roberts JM. Requirement of $p27^{Kip1}$ for restriction point control of the fibroblast cell cycle. Science 272:887-880, 1996.

72. Tan P, Cady B, Wanner M, et al. The cell cycle inhibitor p27 is an independent prognostic marker in small (T1a,b) invasive breast carcinomas. Cancer Res 57:1259-63, 1997.

73. Singh SP, Lipman J, Goldman H, et al. Loss or altered subcellular localizationof p27 in Barrett's associated adenocarcinoma. Cancer Res 58:1730-5, 1998.

74. Yang, RM, Naitoh J, Murphy M, et al. Low p27 expression predicts poor disease-free survival in patients with prostate cancer. J of Urol 159:941-5, 1998.

75. Esposito V, Baldi A, De Luca A, et al. Prognostic role of the cyclin-dependent kinase inhibitor p27 in non-small cell lung cancer. Cancer Res 57:3381-5, 1997.

76. Loda, M, Cukor B, Tam SW, et al. Increased proteasome-dependentdegradation of the cyclin-dependent kinase inhibitor p27 in aggressive colorectal carcinomas. Nat Med 3:231-4, 1997.

77. Fearon ER, Vogelstein B. A genetic model for colorectal tumorigenesis. Cell 61:759-767, 1990.

78. Uchino S, Tsuda H, Noguchi M, Yokota J, Terada M, Saito T, Kobayashi M, Sugimura T, Hifohashi S. Frequent loss of heterozygosity at the DCC locus in gastric cancer. Cancer Res 52:3099-3102, 1992.

79. Hohne MW, Halatsch ME, Kahl GF, Weinel RJ. Frequent loss of expression of the potential tumor suppressor gene DCC in ductal pancreatic cancer. Cancer Res 52:2616-2619, 1992.

80. Gao X, Honn KV, Grignon D, Sakr W, Chen YQ. Frequent loss of expression and loss of heterozygosityof the putative tumor suppressor gene DCC in prostatic carcinomas. Cancer Res 53:2723-2727, 1993.

81. Scheck AC, Coons SW. Expression of the tumor suppressor gene DCC in human Gliomas. Cancer Res 53:5605-9, 1993.

82. Devilee P, Vliet MV, Kuipers-Dijkshorn N, Pearson PL, Cornelisse CJ. Somatic genetic changes on chromosome 18 in breast carcinomas: is the DCC gene involved? Oncogene 6:311-15, 1991.

83. Fearon ER, Cho KR, Nigro JM, Kern SE, Simons JW, Ruppert JM, Hamilton SR, Preisinger AC, Thomas G, Kinzler KW, Vogelstein B. Identification of a chromosome 18q gene which is altered in colorectal cancers. Science 247:49-56, 1990.

84. Hedrick L, Cho KR, Fearon ER, Wu TC, Kinzler KW, Vogelstein B. The DCC gene product in cellular differentiation and colorectal tumorigenesis. Genes and Development 8:1174-1183, 1994.

85. Pierceall WE, Cho KR, Getzenberg RH, Reale MA, Hedrick L, Vogelstein B, Fearon ER. The deleted in colorectal cancer (DCC) tumor suppressor gene product stimulates neurite outgrowth in rat PC12 pheochromocytoma cells. J Cell Bio 124:1017-1027, 1994.

86. Chuong CM, Jiang TX, Yin E, Widelitz RB. cDCC (chicken homologue to a gene deleted in colorectal carcinoma) is an epithelial adhesion molecule expressed in the basal cells and involved in epithelial-mesenchymal interaction. Dev Biol 164:383-97, 1994.

87. Vielmetter J, Kayyem JF, Roman JM, Dreyer WJ. Neogenin, an avian cell surface protein expressed during terminal neuronal differentiation, is closely related to the human tumor suppressor molecule deleted in colorectal cancer. J Cell Biol 127:2009-20, 1994.

88. Fazeli A, Dickinson SL, Hermiston ML, et al. Phenotype of mice lacking functional Deleted in colorectal cancer (Dcc) gene. Nature 386:796-804, 1997.

89. Tanaka K, Oshimura M, Kikuchi R, Seki M, Hayashi T, Miyaki M. Suppression of tumorigenicity in human colon carcinoma cells by introduction of normal chromosome 5 or 18. Nature 349:340-42, 1991.

90. Klingelhutz AJ, Hedrick L, Cho KR, McDougall JK. The DCC gene suppresses the malignant phenotype of transformed human epithelial cells. Oncogene 10:1581-6, 1995.

91. Narayan R, Lawlor KG, Schaapveld RQ, Cho KR, Vogelstein B, Bui-Vinh Tran P, Osborne MP, Telang NT. Antisense RNA to the putative tumor-suppressor gene DCC transforms Rat-1 fibroblasts. Oncogene 7:553-61, 1992.

92. Goi T, Yamaguchi A, Nakagawara, et al. Reduced expression of deleted colorectal carcinoma (DCC) protein in established colon cancers. Br. J. Cancer 77:466-71, 1998.

93. Gotley DC, Reeder JA, Fawcett J, et al. The deleted in colon cancer (DCC) gene is consistently expressed in colorectal cancers and metastases. Oncogene 13:787-95, 1996.

94. Cho KR, Oliner JD, Simons JW, et al. The DCC gene: structural analysis and mutations in colorectal carcinomas. Genomics 19:525-31, 1994.

95. Hahn SA, Schutte M, Hogue AT, et al. DPC4, a candidate tumor suppressor gene at human chromosome 18q21.1. Science 271:350-3, 1996.

96. Eppert K, Scherer SW, Ozcelik H, et al. MADR2 maps to 18q21 and encodes a TGFbeta-regulated MAD-related protein that is functionally mutated in colorectal carcinoma. Cell 86:543-52, 1996.

97. Thiagalingam S, Lengauer C, Leach FS, et al. Evaluation of candidate tumour suppressor genes in chromosome 18 in colorectal cancers. Nat Genetics 13:343-6, 1996.

98. Iino H, Fukayama M, Maeda Y, Koike M, Mori T, Takahashi T, Kikuchi-Yanoshita R, Miyaki M, Mizuno S, Watanabe S. Molecular genetics for clinical management of colorectal carcinoma. Cancer 73:1324-1331, 1994.

99. Jen J, Kim H, Piantadosi S, Lis ZF, Levitt RC, Sistonen P, Kinzler KW, Vogelstein B, Hamilton SR. Allelic loss of chromosome 18q and prognosis in colorectal cancer. N Engl J Med 331:213-21, 1994.

100. Ogunbiyi OA, Goodfellow PJ, Herfarth K, et al. Confirmation that chromosome 18q allelic loss in colon cancer is a prognostic indicator. J Clin Oncol 16:427-33, 1998.

101. Martinez-Lopez E, Abad a, Font A, et al. Allelic loss on chromosome 18q as a prognostic marker in stage II colorectal cancer. Gastroenterology 114:1180-7, 1998.

102. Carethers JM, Hawn MT, Greenson JK, et al. Prognostic significance of allelic lost at chromosome 18q21 for stage II colorectal cancer. Gastroenterology 114:1188-95, 1998.

103. Shibata D, Reale MA, Lavin P, et al. The DCC protein and prognosis in colorectal cancer. NE J Med 335:1727-32, 1996.

104. Folkman J. The role of angiogenesis in tumor growth. Sem Cancer Biol 3:65-71, 1992.

105. Folkman J and Shing Y. Angiogenesis. J Biol Chem 267: 10931-10934, 1992.

106. Weidner N, Folkman J, Pozza F, Bevilacqua P, Allred EN, Moore DH, Meli S and Gasparini G. Tumor angiogenesis: a new significant and independent prognostic indicator in early-stage breast carcinoma. J Natl Cancer Inst 84(24): 1875-1887, 1992.

107. Gasparini G, Harris AL. Clinical importance of the determination of tumor angiogenesis in breast carcinoma: Much more than a new prognostic tool. J Clin Oncol 13:7652-7782, 1995.

108. Bevilacqua P, Barbareschi M, Verderio P, Boracchi P, Caffo O, Dalla Palma P, Meli S, Weidner N and Gasparini G. Prognostic value of intratumoral microvessel density, a measure of tumor angiogenesis, in node-negative breast carcinoma--results of a multiparametric study. Br Cancer Res and Treat 36(2): 205-17, 1995.

109. Bremer GL, Tiebosch AT, van der Putten HW, Schouten HJ, de Haan J and Arends JW. Tumor angiogenesis: an independent prognostic parameter in cervical cancer. Am J Ob and Gyn 174(1Pt 1): 126-31, 1996.

110. Bochner BH, Cote RJ, Weidner N, Groshen S, Chen SC, Skinner DG and Nichols PW. Angiogenesis in bladder cancer: relationship between microvessel density and tumor prognosis. J Natl Cancer Inst 87(21): 1603-12, 1995.

111. Weidner N. Tumor angiogenesis: review of current applications in tumor prognostication. Sem Diag Path 10(4): 302-13, 1993.

112. Hollingsworth HC, Kohn EC, Steinberg SM, Rothenberg ML and Merino MJ. Tumor angiogenesis in advanced stage ovarian carcinoma. Am J Path 147(1): 33-41, 1995.

113. Maeda K, Chung YS, Takatsuka S, Ogawa Y, Sawada T, Yamashita Y, Onoda N, Kato Y, Nitta A, Arimoto Y, et al. Tumor angiogenesis as a predictor of recurrence in gastric carcinoma. J Clin Oncol 13(2): 477-81, 1995.

114. Ferrara N and Davis-Smyth T. The biology of vascular endothelial growth factor. Endocrine Reviews 18: 4-25, 1997.

115. Warren RS, Yuan H, Matli MR, Gillett NA and Ferrara N. Regulation by vascular endothelial growth factor of human colon cancer tumorigenesis in a mouse model of experimental liver metastasis. J Clin Invest 95:1789-1797, 1995.

116. Takahashi Y, Kitadai Y, Bucana CD, Cleary KR and Ellis LM. Expression of vascular endothelial growth factor and its receptor, KDR, correlates with vascularity, metastasis, and proliferation of human colon cancer. Cancer Res 55:3964-3968, 1995.

117. Frank RE, Saclarides TJ, Leurgans S, Speziale NJ, Drab EA and Rubin DB. Tumor angiogenesis as a predictor of recurrence and survival in patients with node-negative colon cancer. Annals of Surg 222(6): 695-699, 1995.

118. Bossi P, Viale G, Lee AKC, Alfano R, Coggi G and Bosari S. Angiogenesis in colorectal tumors: microvessel quantitation in adenomas and carcinomas with clinicopathological correlations. Cancer Res 55:5049-5053, 1995.

119. Tomisaki S, Ohno S, Ichiyoshi Y, et al. Microvessel quantification and its possible relation with liver metastasis in colorectal cancer. Cancer 77(8 Suppl): 1722-8, 1996.

120. Takahashi Y, Bucana CD, Cleary KR, et al. p53, vessel count, and vascular endothelial growth factor expression in human colon cancer. Intl. J Cancer 79:34-8, 1998.

121. Kumar H, Heer K, Lee PW, et al. Preoperative serum vascular endothelial growth factor can predict stage in colorectal cancer. Clin Cancer Res 4:1279-85, 1998.

122. Berney CR, Yang JL, Fisher RJ, et al. Vascular endothelial growth factor expression is reduced in liver metastasis from colorectal cancer and correlates with urokinase-type plasminogen activator. Anticancer Res 18:973-7, 1998.

123. Linderholm B, Tavelin B, Grankvist K, et al. Vascular endothelial growth factor is of high prognostic value in node-negativebreast carcinoma. J Clin Oncol 16:3121-28, 1998.

124. Eppenberger U, Kueng W, Schlaeppi J-M, et al. Markers of tumor angiogenesis and proteolysis independently define high- and low-risk subsets of node-negative breast cancer patients. J Clin Oncol 16:3129-36, 1998.

125. Mukhopadhyay D, Tsoikas L and Sukhatme VP. Wild-type p53 and v-Src exert opposing influences on human vascular endothelial growth factor gene expression. Cancer Res 55:6161-5, 1995.

126. Kieser A, Weich HA, Brander G, et al. Mutant p53 potentiates protein kinase C induction of vascular endothelial growth factor expression. Oncogene 9:963-9, 1994.

127. Dameron KM, Volpert OV, Tainsky MA, et al. Control of angiogenesis in fibroblasts by p53 regulation of thrombospondin-1. Science 265:1582-4, 1994.

128. Berger SH, Jenh CH, Johnson LF, and Berger FG. Thymidylate synthase overproduction and gene amplificationin fluorodeoxyuridine-resistanthuman cells. Mol Pharmacol 28:461-467, 1985.

129. Swain SM, Lippman ME, Egan EF, Drake JC, Steinberg SM and Allegra CJ. Fluorouracil and high doses leucovorin in previously treated patients with metastatic breast cancer. J Clin Oncol 7:890-899, 1989.

130. Leichman L, Lenz H-J, Leichman CG, Groshen S, Danenberg KD, Baranda J, Spears CP, Boswell W, Silberman H, Ortega A, Stain S, Beart R and Danenberg PV. Quantitation of intratumoral thymidylate synthase expression predicts for resistance to protracted infusion of 5-fluorouracil and weekly leucovorin in disseminated colorectal cancers: Preliminary report from an ongoing trial. Eur J Cancer 31: 1306-1310, 1995.

131. Lenz H-J, Leichman CG, Danenberg KD, Danenberg PV, Groshen S, Cohen H, Laine L, Crookes P, Silberman H, Baranda J, Garcia Y, Li J, Leichman L. Thymidylate synthase expression in adenocarcinoma of the stomach: A predictor for primary tumor response and overall survival. J Clin Oncol 14:176-182, 1996.

132. Horikoshi T, Danenberg KD, Stadlbaur THW, et al. Quantitation of thymidylate synthase, dihydrofolate reductase, and DT-diaphorase gene expression in human tumors using the polymerase chain reaction. Cancer Res 52:108-116, 1992.

133. Johnston PG, Liang CM, Henry S, et al. The production and characterization of monoclonal antibodies that localize human thymidylate synthase in the cytoplasm of human cells and tissues. Cancer Res 51: 6668-6676, 1991.

134. Johnston PG, Lenz HJ, Danenberg KD, et al. Thymidylate synthase protein and gene expression predicts for response to 5-fluorouracil/leucovorin chemotherapy in patients with colorectal and gastric cancer. Cancer Res 55:1407-1412, 1995.

135. Johnston PG, Fisher ER, Rockette, HE, et al. The role of thymidylate synthase expression in prognosis and outcome to adjuvant chemotherapy in patients with rectal cancer. J Clin Oncol 12:2640-2647, 1994.

136. Johnston PG, Allegra CJ: ColorectalCancer Biology. Clinical Implications.Seminars in Oncology 5:418-432, 1995.

137. Banerjee D, Lenz H-J, Schnieders B, Kheradpour A, Manno DJ, Danenberg KD, Danenberg PV and Bertino JR. Transfection of wild type but not mutant p53 induces early monocytic differentiation in HL-60 cells. Cell Growth & Differ 6:1405-1413, 1995.

138. Lenz HJ, Danenberg KD, Leichman CG, et al. p53 and thymidylate synthase expression in untreated stage II colon cancer: associations with recurrence, survival, and site. Clin Cancer Res 4:1227-34, 1998.

139. Jansen WJ, Zwart B, Hulscher ST, et al. CPT-11 in human colon-cancercell lines and xenografts: characterization of cellular sensitivity determinants. Intl J Cancer 70:335-40, 1997.

140. McLeod HL and Keith WN. Variation in topoisomerase 1 gene copy number as a mechanism for intrinsic drug sensitivity. Br J Cancer 74:508-12, 1996.

141. Weidner N. Current pathologic methods for measuring intratumoral microvessel density within breast carcinoma and other solid tumors. Br Cancer Res and Treat 36(2): 169-80, 1995.

142. Kraemer HC. Extension of the Kuppa Coefficient. Biometrics 36:207-16, 1980.

143. Cox DR. Regression models and life tables (with discussion). J Roy Statist Soc B 34:187-220, 1972.

144. Rubinstein LV, Gail MH and Santner TJ. Planning the duration of a comparative clinical trial with loss to follow-up and a period of continued observation. J Chron Dis. 34: 469-79, 1981.

145. Fleiss JL. Statistical Methods for Rates and Proportions, New York, Wiley, 1981.

146. Cox DR. A note on the graphical analysis of survival data. Biometrika 66:188-190, 1979.

147. Schoenfeld D. Goodness of fit tests for the proportional hazards regression model. Biometrika 67:145-154, 1980.

148. Andersen PK. Testing goodness of fit of Cox's regression and life model. Biometrics 38:67-77, 1982.

149. Hochberg Y. A sharper Bonferroni procedure for multiple tests of significance. Biometrika 75: 800-2, 1988.

APPENDIX II

Data Collection Forms

C-584	GI Adjuvant Intergroup On-Study Form
C-447	CALGB Sample Tracking Form--Blocks
C-586	CALGB GI Adjuvant Treatment Form
C-528	CALGB 89803 Toxicity Form
C-585	CALGB GI Adjuvant Follow-up and Recurrence Form
C-300	CALGB: Off Treatment Notice
C-215	CALGB Secondary Malignancy Form
C-113	CALGB Notification of Death Form
C-260	CALGB Remarks Addenda

APPENDIX III

Clinical Trials Agreement (CTA) Language

The agent (hereinafter referred to as "Agent"), CPT-11 (irinotecan), used in this protocol is provided to the NCI under a Clinical Trials Agreement (CTA) between Pharmacia-UpJohn (hereinafter referred to as "Collaborator") and the NCI Division of Cancer Treatment, Diagnosis and Center. Therefore, the following obligations/guidelines apply to the use of the Agent in this study:

1. Agent may not be used outside the scope of this protocol, nor can Agent be transferred or licensed to any party not participating in the clinical study. Collaborator data for Agent are confidential and proprietary to Collaborator and should be maintained as such by the investigators.

2. For a clinical protocol where there is an investigational Agent used in combination with (an)other investigational Agent(s), each the subject of different CTAs or CRADAs, the access to and use of data by each Collaborator shall be as follows (data pertaining to such combination use shall hereinafter be referred to as "Multi-Party Date"):

   a. NCI must provide all Collaborators with written notice regarding the existence and nature of any agreements governing their collaboration with NIH, the design of the proposed combination protocol, and the existence of any obligation which would tend to restrict NCI's participation in the proposed combination protocol.

   b. Each Collaborator shall agree to permit the use of the Multi-Party Data from the clinical trial by any other Collaborator to the extent necessary to allow said other Collaborator to develop, obtain regulatory approval or commercialize its own investigational Agent.

   c. Any Collaborator having the right to use the Multi-Party Data from these trials must agree in writing prior to the commencement of the trials that it will use the Multi-Party Data solely for development, regulatory approval, and commercialization of its own investigational Agent.

3. The NCI encourages investigators to make data from clinical trials fully available to Collaborator for review at the appropriate time (see #5). The NCI expects that clinical trial data developed under a CTA or CRADA will be made available exclusively to Collaborator, and not to other parties.

4. When a collaborator wishes to initiate a data request, the request should first be sent to the NCI, who will then notify the appropriate investigators (Group Chair for cooperative group studies, or PI for other studies) of Collaborator's wish to contact them.

5. Any data provided to Collaborator must be in accordance with the guidelines and policies of the responsible Data Monitoring Committee (DMC), if there is a DMC for this clinical trial.

6. Any manuscripts reporting the results of this clinical trial should be provided to CTEP for immediate delivery to Collaborator for advisory review and comment prior to submission for publication. Collaborator will have 30 days from the date of receipt for review. An additional 30 days may be requested in order to ensure that confidential and propriety data, in addition to Collaborator's intellectual property rights, are protected. Copies of abstracts should be provided to Collaborator for courtesy review following submission, but prior to presentation at the meeting or publication in the proceedings. Copies of any manuscript and/or abstract should be sent to:

Regulatory Affairs Branch, CTEP, DCTDC, NCI
Executive Plaza North, Room 718
Bethesda, Maryland 20892
Fax 301-402-1584

The Regulatory Affairs Branch will then distribute them to Collaborator.

APPENDIX IV

A PROSPECTIVE STUDY OF DIET AND OTHER LIFESTYLE FACTORS AMONG PATIENTS WITH STAGE III COLON CANCER

A COMPANION STUDY TO CALGB 89803

Charles Fuchs, M.D.
Dana Farber Cancer Institute
44 Binney St.
Boston, MA 02115
Phone: (617) 632-2225

I. BACKGROUND

In 1999, approximately 132,000 Americans are expected to develop colorectal cancer, and 55,000 individuals will die from the disease (1). Epidemiologic and scientific research indicate that diet and other lifestyle factors have a significant influence on the risk of developing colon cancer. Consumption of red meat (2,3), alcohol (4,5), calcium (6), fiber (7), and folic acid (4,5,8,9), obesity, physical activity (10-12), and cigarette smoking (13,14) are among factors that have been suggested to influence the risk of developing colorectal cancer.

Little is known about the influence of diet and other factors on the outcome for individuals with established colon cancer. Randomized clinical trials demonstrate a significant survival advantage among individuals with stage III colon cancer who receive adjuvant fluorouracil-based chemotherapy. Nonetheless, 40-45% of stage III patients receiving current adjuvant chemotherapy will develop metastases. Patients often seek to understand what, if any, diet and lifestyle changes will reduce their chances of recurrence of colon cancer as well as the potential toxicities associated with adjuvant therapy. In conjunction with this treatment trial of patients with stage III colon cancer, we propose the following specific aims:

II. SPECIFIC AIMS

1. To prospectively assess the influence of diet, body mass index, and physical activity on the risk of cancer-recurrence and survival among patients with stage III colon cancer.

2. To assess the influence of diet, obesity, and physical activity on the risk of toxicity associated with adjuvant therapy.

3. In exploratory analyses, we also propose to assess whether the influence of adjuvant therapy on the risk of cancer-recurrence is modified by dietary habits. In addition, we will explore the interaction of diet and molecular markers within tumors on the prognosis of patients with stage III colon cancer.

III. HYPOTHESES

As part of our analysis to prospectively assess the influence of diet, body mass index, and physical activity on the risk of cancer-recurrence and survival among patients with stage III colon cancer, we will address the following hypotheses:

1. **Regular physical activity and avoidance of obesity reduce the risk of cancer-recurrence and mortality.**

   Sedentary life-style and obesity are each associated with an increased risk of developing colon cancer. Among several studies, the most physical active participants experienced a 50 percent reduction in risk (relative risk = 0.50) (10-12). Both factors appear to act late in the pathway of colon carcinogenesis. Moreover, recent observations indicate that each factor may be mediated by enhancing levels of Insulin-like Growth Factors, known trophic hormones for colon carcinogenesis (15,16). No study has assessed the influence of sedentary life-style and obesity among patients with established colon cancer.

2. **Increased red meat and animal fat consumptionincrease the risk of cancer-recurrence and mortality.**

   Increased red meat intake is associated with an increased risk of developing colon cancer. Among participants in the Nurses' Health and Health ProfessionalsFollow-up Study, the highest consumers of red meat experienced a 2.75 to 3.5 fold increase in risk (1-3). No study has assessed the influence of red meat among patients with established colon cancer.

3. **Regular aspirin use (greater than two 325 mg tablets per week on average) reduces the risk of cancer-recurrence and mortality.**

   Regular aspirin use (greater than two 325 mg tablets per week on average; coded as yes or no) has been associated with 40% reduction in the risk of developing colon cancer and adenoma (17-20). No study has assessed the influence of aspirin among patients with established colon cancer.

4. **Increased vitamin E intake (coded in quintiles; with and without supplements) reduces the risk of cancer-recurrence and mortality.**

   Recent studies suggest that vitamin E may enhance the cytotoxicity of 5-fluorouracil on colon cancer cells. Chinery et al. observed that the antioxidants pyrrolidinedithiocarbamate and vitamin E induced apoptosis in colorectal cancer cells (21). This effect was mediated by induction of p21WAF1/CIP1, a powerful inhibitor of the cell cycle, through a mechanism involving C/EBPbeta (a member of the CCAAT/enhancer binding protein family of transcription factors), independent of p53. Antioxidants significantly enhanced colorectal cancer tumor growth inhibition by 5-fluorouracil in vitro and in vivo. To date, no prospective studies have assessed this relation in the adjuvant setting.

5. **Increased folate intake reduces the risk of cancer-recurrence and mortality.**

   Studies suggest that increased intake of fruits and vegetables reduces the risk of developing colon cancer, possibly mediated through the folic acid content in fruits and vegetables(1) Recent studies show that increased folic acid intake reduces the risk of developing colon cancer by more than 40% (4,5,8,9). No study has assessed the influence of folate intake among patients with established colon cancer.

In further exploratory analyses, we will also consider looking at the relationship between calcium, vitamin D, fiber and alcohol intake, as well as smoking, on the risk of cancer-recurrence and mortality.

IV. METHODS

Patients will be given a 131-item validated, food-frequency questionnairemidway through their adjuvant therapy (four months following surgical resection) and then at six months after completion of adjuvant therapy (14 months following surgical resection). The questionnaire, designed by Willett and colleagues for the Nurses' Health Study, has been extensively validated among both health professional and lay populations and provides comprehensive data on over 100 micro-nutrients, with and without supplement use. This questionnaire will be self-administered. Patients are to complete the questionnaire at their follow-up appointments and return the questionnaire before leaving the office.

Within the questionnaire, a series of questions about leisure-time physical activity that have also been validated in large populations will also be included. Height and weight will also be obtained as part of the adjuvant therapy.

Validation of the Semi-quantitative Food Frequency Questionnaire

The current version of the questionnaire consists of 131 food items plus vitamin and mineral supplement use that collectively account for over 90% of the intake of the nutrients assessed (22-24). For each food, a commonly used unit or portion size (e.g., one egg or slice of bread) is specified, and participants are asked how often, on average over the past year, they consumed that amount of each food. There are nine possible responses, which range from never to six or more times per day. Nutrient intakes will be computed by multiplying the frequency of consumption of each food by the nutrient content of the specified portions, using composition values from Department of Agriculture sources supplemented with other data, including the components of specific vitamins and breakfast cereals. All nutrients will be adjusted for total energy intake by the residuals method (25).

In 1980, the food frequency questionnaire was administered twice to 173 individuals at an interval of approximately one year, and four one-week diet records for each subject were collected during that period. Diet records probably are the best measures of current, short-term food intake. Since the seven-day record provides information for a relatively short period of time, four one-week diet records in different seasons were collected. The mean calorie adjusted intakes from the four one-week diet records and those from the questionnaire were well-correlated (22-24). In the 1986 diet validation study, the correlation between folate calculated from the semi-quantitative food frequency questionnaire (SFFQ) and red cell folate level was 0.55 (4). Nutrients calculated from the expanded SFFQ were correlated with other corresponding biochemical indicators: plasma beta-carotene (r = 0.30-0.42) (26,27), plasma vitamin E (r = 0.41-0.53) (26,27), adipose linoleic acid (r = 0.35-0.37) (28,29), adipose *trans* fatty acid (r = 0.51) (28,29), and adipose N-3 fatty acids (r = 0.48-0.49) (28,29).

To evaluate further the capability of the revised 131-item questionnaire to discriminate among subjects, we asked 127 individuals to complete two weeks of diet records and the semi-quantitative food frequency questionnaire in 1986. The mean calorie adjusted intakes from the diet records and those from the questionnaire were well-correlated (24). The correlation between folate intake on the questionnaire and the measured erythrocyte folate level was statistically significant.

These data indicate that the self-administered dietary questionnaires provides highly informative and biologically relevant measurement of a wide variety of nutrients, thus allowing one to address the dietary hypotheses outlined in the specific aims.

A detailed validation study was conducted of the physical activity questionnaire among a sample of 325 participants in the parallel Nurses' Health Study II (NHS II) (241 random

cohort sample and 84 random sample of African American participants) (30). Participants completed four 1-week activity recalls and four 7-day activity diaries over one year and then repeated the NHS II activity questionnaire. For the total activity score, the correlations of the last activity questionnaire with the diaries was 0.64 for the total cohort sample and 0.59 for the African American sample. Within the Health Professionals Follow-up Study, a parallel study of men, validity of the physical activity questionnaire was assessed among 238 randomly selected participants by comparisons with four 1-week activity diaries, four 1-week activity recalls, and resting and post exercise pulse rates. Correlations with the activity diaries were 0.41 for inactivity (sitting) and 0.58 for vigorous physical activity. Vigorous activity assessed by the questionnaire was correlated with resting pulse (r = -0.45) and post-exercise pulse (r = -0.41). Further, the same measure of recent activity was shown to be predictive of colon cancer in women (12), and men (10,11), and diverticular disease (31), and gallstones (32) in men.

V. ANALYSIS

The influence of various dietary constituentson the rate of recurrence and survival among these patients will be assessed. Among the factors we propose to assess include: intake of red meat, calcium, vitamin D, alcohol, fiber, vitamin E, other anti-oxidants, methionine, and folic acid. We will also look at the influence of body mass index and physical activity on the outcome of these patients. We will principally use the questionnaire data provided at 14 months after surgical resection (6 months after completion of adjuvant therapy) for our analysis. We will secondarily use the data obtained during adjuvant therapy.

We will also assess the influence of diet and other factors on toxicities associated with adjuvant therapy. We will principally use questionnaire data obtained during adjuvant therapy for this analysis.

Lastly, we will explore the interaction of diet and other factors with various molecular markers (see Methods) on the outcome of patients with stage III colon cancer.

VI. STATISTICAL CONSIDERATIONS

The impact of the following dietary variables on colon cancer progression and toxicity will be assessed at two timepoints: 1) regular physical activity and avoidance of obesity; 2) increased red meat and animal fat consumption; 3) regular aspirin use; 4) increased vitamin E intake; 5) increased folate intake.

Progression will be measured from trial entry until documented progression of disease or death from any cause. Patients will be followed for recurrence and survival for 7 years after the end of treatment (Section 13.3). Toxicity will be measured using two endpoints: 1) the proportion of patients experiencing Grade 3 or greater diarrhea; 2) the proportion of patients experiencing Grade 3 or greater neutropenia. Patients will be surveyed at 4 and 14 months post surgical resection. Results at each of the two timepoints will be compared descriptively.

The median survival in this patient population treated with adjuvant 5FU/LV is assumed to be 8 years. We, therefore, expect that approximately 97% and 90% of patients will survive 4 months and 14 months post surgery, respectively. One thousand two hundred and sixty (1,260) patients will be randomized on this trial in 2.8 years and followed an additional 3.0 years. Analysis of the diet and lifestyle questionnaires will occur approximately 5.8 years after study activation. The approximate 3-year recurrence rate in this patient population is assumed to be 35%.

Patients will be asked to complete the self-administered questionnaire at the given times post surgery. Responses to the questionnaire will be dichotomous or assumed to have an underlying continuous distribution summarized using quintiles.

Power Estimation

Power estimation is based on proportions of patients progression-free at 3 years, univariate tests of hypothesis testing at significance levels 0.01 and 0.05 and hypothetical results according to three of the five primary hypotheses. Table B1 provides power estimates for testing the impact of regular aspirin use, expressed as a dichotomized variable, on progression at 3 years (Hypothesis 3.).

TABLE B1

Power to detect a 3-year difference in progression of 0.125 in magnitude (0.375 for non-regular aspirin users versus 0.25 for regular aspirin users) with 20% of patients responding 'yes' to regular aspirin use (1-sided chi-square test at significance levels 0.01 and 0.05) by numbers of patients responding to survey.

N	Significance level 0.01	0.05
1134	0.83	0.98
1000	0.77	0.97
800	0.65	0.93
600	0.50	0.85

For variables expressed as quintiles power was estimated for testing the null hypothesis of independence versus the alternative of a linear trend in the 3-year progression rate over quintiles. An alternative hypothesis for red meat consumption is illustrated in Table B2. These differences correspond to a maximum odds ratio (5th quintile versus 1st) of 2.47. Power estimates to detect this alternative for several sample sizes are given in Table B3 (Hypothesis 2.).

TABLE B2

Quintile	1st	2nd	3rd	4th	5th	
Recurred	0.05	0.06	0.07	0.08	0.09	0.35
Not Recurred	0.15	0.14	0.13	0.12	0.11	0.65

TABLE B3
Power to detect a linear trend in 3-year progression over red meat consumption expressed in quintiles (significance levels 0.01 and 0.05) by numbers of patients responding to survey.

N	Significance level 0.01	0.05
1134	0.85	0.95
1000	0.78	0.92
800	0.65	0.84
600	0.48	0.71

Power for the toxicity endpoint, Grade 3 or greater neutropenia, was computed within treatment group. Based on previously reported data we expect approximately 35% and 7% of patients receiving CPT-11/5FU/LV and 5FU/LV alone, respectively, to experience Grade 3 or greater neutropenia. Table 4 provides power estimates to detect a difference of 0.17 in magnitude in Grade 3+ neutropenia between patients CPT-11/5FU/LV who are obese and those who are not (CPT-11/5FU/LV arm). Forty percent (40%) of patients are assumed to be obese (33). Comparable power is achieved to detect a difference of 0.095 in magnitude (0.03 for non-obese patients versus 0.125 for obese patients) for patients receiving 5FU/LV alone (Hypothesis 1.).

TABLE B4
Power to detect a toxicity difference (Grade 3+ neutropenia) of 0.17 in magnitude (0.28 for non-obese patients versus 0.45 for obese patients) with 40% of patients meeting obesity criteria (1-sided chi-square test at significance levels 0.01 and 0.05) by numbers of patients responding to survey

N	Significance level 0.01	0.05
570	0.69	0.87
500	0.62	0.82
400	0.50	0.73

The Cox model will be used to explore the simultaneous effect of these variables on recurrence and survival. Logistic regression will be used to explore the relationship between the study variables and toxicity outcomes. Exploratory analyses will also consider the relationships between other dietary factors such as calcium intake, vitamin D, fiber, alcohol intake and smoking habits on the risk of recurrence, toxicity and mortality. Models incorporating both dietary and molecular markers will be studied.

References

1. Potter J. Colorectal cancer: molecules and populations. *J Natl Cancer Inst* 91:916-32, 1999.

2. Willett WC, Stampfer MJ, Colditz GA, Rosner BA, Speizer FE. Relation of meat, fat, and fiber intake to the risk of colon cancer in a prospective study among women [see comments]. *N Engl J Med* 323:1664-72, 1990.

3. Giovannucci E, Rimm E, Stampfer M, Colditz G, Ascherio A, Willett W. Intake of fat, meat, and fiber in relation to risk of colon cancer in men. *Cancer Res* 54:2390-2397,1994.

4. Giovannucci E, Stampfer MJ, Colditz GA, et al. Folate, methionine, and alcohol intake and risk of colorectal adenoma [see comments]. *J Natl Cancer Inst* 85:875-84, 1993.

5. Giovannucci E, Rimm EB, Ascherio A, Stampfer MJ, Colditz GA, Willett WC. Alcohol, low-methionine--low-folate diets, and risk of colon cancer in men [see comments]. *J Natl Cancer Inst* 87:265-73, 1995.

6. Baron J, M B, JS M, et al. Calcium supplements for the prevention of colorectal adenomas. Calcium Polyp Prevention Study Group. *N Eng J Med* 340:101-7, 1999.

7. Howe G, Benito E, Castelleto R, et al. Dietary intake of fiber and decreased risk of cancers of the colon and rectum: evidence from the combined analysis of 13 case-control studies. *J Natl Cancer Inst* 84:1887-1896, 1992.

8. Giovannucci E, Stampfer MJ, Colditz GA, et al. Multivitamin use, folate, and colon cancer in women in the Nurses' Health Study [see comments]. *Ann Intern Med* 129:517-24, 1998.

9. Glynn S, Albanes D. Folate and cancer: a review of the literature. *Nutr Cancer* 22:101-19, 1994.

10. Giovannucci E, Colditz GA, Stampfer MJ, Willett WC. Physical activity, obesity, and risk of colorectal adenoma in women (United States). *Cancer Causes Control* 7:253-63, 1996.

11. Giovannucci E, Ascherio A, Rimm EB, Colditz GA, Stampfer MJ, Willett WC. Physical activity, obesity, and risk for colon cancer and adenoma in men. *Ann Intern Med* 122:327-34, 1995.

12. Martinez ME, Giovannucci E, Spiegelman D, Hunter DJ, Willett WC, Colditz GA. Leisure-time physical activity, body size, and colon cancer in women. Nurses' Health Study Research Group. *J Natl Cancer Inst* 89:948-55, 1997.

13. Giovannucci E, Rimm E, Stampfer M, et al. A prospective study of cigarette smoking and risk of colorectal adenoma and colorectal cancer in U.S. men. *J Natl Cancer Inst* 86:183-191, 1994.

14. Giovannucci E, Colditz G, Stampfer M, et al. A prospective study of cigarette smoking and risk of colorectal adenoma and colorectal cancer in U.S. women. *J Natl Cancer Inst* 86:192-199, 1994.

15. Ma J, Pollak M, Giovannucci E, et al. Prospective study of colorectal cancer risk in men and plasma levels of insulin-like growth factor and IGF-binding protein-3. *J Natl Cancer Inst* 91:620-5,1999.

16. Giovannucci E, Pollak M, Platz E, et al. Plasma insulin-like growth factor-I and binding protein-3 and risk of colorectal cancer and adenoma in women. submitted, 1999.

17. Paganini-Hill A, Hsu G, Ross R. Aspirin use and reduced risk of fatal colon cancer. *N Engl J Med* 326:1290-1291, 1992.

18. Thun M, Calle E, Namboodiri M, Heath C. Aspirin use and reduced risk of fatla colon cancer. *N Engl J Med* 325:1593-1596, 1991.

19. Giovannucci E, Egan K, Hunter D, et al. Aspirin and the risk of colorectal cancer in women. *New Engl J Med* 333:609-614, 1995.

20. Giovannucci E, Rimm E, Stampfer M, Colditz G, Ascherio A, Willett W. Aspirin use and the risk for colorectal cancer and adenoma in male health professionals. *Ann Intern Med* 121:241-246, 1994.

21. Chinery R, Brockman JA, Peeler MO, Shyr Y, Beauchamp RD, Coffey RJ. Antioxidants enhance the cytotoxicity of chemotherapeutic agents in colorectal cancer: a p53-independent induction of p21WAF1/CIP1 via C/EBPbeta [see comments]. *Nat Med* 3:1233-41, 1997.

22. Willett WC, Sampson L, Stampfer MJ, et al. Reproducibility and validity of a semiquantitative food frequency questionnaire. *Am J Epidemiol* 122:51-65, 1985.

23. Willett W, Stampfer M, Underwood B, et al. Validation of dietary questionnaire with plasma carotenoid and alpha-tocopherol level. *Am J Clin Nutr* 38:631-639, 1988.

24. Willett W, Sampson L, Browne M, et al. The use of a self-administered questionnaire to assess diet four years in the past. *Am J Epidemiol* 127:188-199, 1988.

25. Willett W, Stampfer M. Total energy intake: implications for epidemiologic analysis. *Am J Epidemiol* 124:17-27, 1986.

26. Stryker WS, Salvini S, Stampfer MJ, Sampson L, Colditz GA, Willett WC. Contributions of specific foods to absolute intake and between-person variation of nutrient consumption. *J Am Diet Assoc* 91:172-8, 1991.

27. Ascherio A, Stampfer M, Colditz G, Rimm E, Litin L, Willett W. Correlations of vitamin A and E intakes with the plasma concentrations of carotenoids and tocopherols among American men and women. *J Nutr* 122:1792-1801, 1992.

28. Hunter DJ, Rimm EB, Sacks FM, et al. Comparison of measures of fatty acid intake by subcutaneous fat aspirate, food frequency questionnaire, and diet records in a free- living population of US men [see comments]. *Am J Epidemiol* 135:418-27, 1992.

29. London SJ, Sacks FM, Caesar J, Stampfer MJ, Siguel E, Willett WC. Fatty acid composition of subcutaneous adipose tissue and diet in postmenopausal US women. *Am J Clin Nutr* 54:340-5, 1991.

30. Wolf AM, Hunter DJ, Colditz GA, et al. Reproducibility and validity of a self-administered physical activity questionnaire. *Int J Epidemiol* 23:991-9, 1994.

31. Aldoori WH, Giovannucci EL, Rimm EB, et al. Prospective study of physical activity and the risk of symptomatic diverticular disease in men [see comments]. *Gut* 36:276-82, 1995.

32. Leitzmann MF, Giovannucci EL, Rimm EB, et al. The relation of physical activity to risk for symptomatic gallstone disease in men. *Ann Intern Med* 128:417-25, 1998.

33. Felgal K, Carroll M, Kuczmarski R, Johnson C. Overweight and obesity in the United States: prevalence and trends, 1960-1994. *Int J Obes Relat Metab Disord* 22:39-47, 1998.

APPENDIX V

Expanded Participation Project (EPP) Instructions

Expanded Participation Project (EPP) Instructions

1.0 EPP RANDOMIZATION AND REGISTRATION PROCEDURES

I. EPP institutions will register a patient on-line through the Clinical Trials Management Unit (CTMU). Questions pertaining to eligibility criteria should be directed to the CTMU, medical questions should be directed to the Study Chair.

II. A signed HHS 310 form documenting the Institutional Review Board (IRB) approval for this study must be on file at the CTMU before the EPP institution can enter a patient. IRB approval date must be less than one year prior to the date of registration.

III. Once eligibility is confirmed, the CTMU will contact CALGB to randomize the patient. The CTMU will notify the institution by an email upon successful enrollment with CALGB. In addition CALGB will forward confirmation of randomization and treatment assignment to the CTMU for routing to the participating institutions.Please check for errors, and submit any corrections on-line to the CTMU.

2.0 EPP DATA SUBMISSION

Data must be submitted electronically directly to the CTMU according to the following schedule:

FORM	TIME OF SUBMISSION
1. CALGB 89803 Eligibility Checklist	At registration
2. CALGB 89803 GI Adjuvant Intergroup On-Study Form (C-584)<br>*(Prestudy Radiologic, Operative and Pathology Reports*)***	Within 1 week of registration
3. EPP Pathology Submission Form**	Within 1 month of registration
4. EPP Toxicity Form	Months 1, 2, 3, and every three months while on protocol therapy
5. EPP Follow-up Form**	Every 3 months while on protocol treatment and every 6 months after completion of protocol treatment until death
6. EPP Recurrence Form	At the time of recurrence
7. EPP Chemotherapy/ Immunotherapy/Hormonal Therapy Form	At the completion of protocol therapy
8. EPP Off-Treatment Form**	At the completion of all protocol therapy
9. EPP Notice of Secondary Malignancy Form	Within 10 days of diagnosis
10. EPP Death Form	Within 7 days of knowledge of event

* A copy of the documents must be faxed to the CTMU Attn: EPP Protocol Monitor 301-299-3991

** These forms are to be submitted according to the above schedule for all patients who never started treatment.

3.0 EPP PATHOLOGY SUBMISSION

The following materials will be required for each patient entered on the study (refer to section 6.7 of the protocol for details):

- At least one (three if possible) paraffin embedded block (s) with REPRESENTATIVE TUMOR
- One paraffin embedded block with NORMAL COLONIC MUCOSA OR UNINVOLVED LYMPH NODE

  The above samples must be properly identified with patient's name and institution, CALGB patient number and CALGB protocol number (this information will be listed on the EPP Pathology Submission Form)

- EPP Pathology Submission Form can be printed upon on-line submission using CTL-P buttons.
- A copy of the responsible pathologist's pathology report from the TREATING institution, and, if applicable, the REFERRING institution.
- A copy of the operative report

The required materials must be submitted within 1 month of patient registration to:

CALGB Pathology Coordinating Office
The Ohio State University
B054 Graves Hall
333 West 10th Avenue
Columbus, OH 43210-1239
Phone: (614) 688-3495
Fax: (614) 292-5618

4.0 EPP ADVERSE DRUG REACTIONS

For EPP Institutions, all ADRs are to be faxed to the CTMU (Attn: EPP Protocol Monitor 301-299-3991) using the Adverse Reaction (ADR) Form for Investigational Drugs. These reports will be reviewed and directed to CALGB and appropriate regulatory offices. ADR reporting is based on the revised NCI Common Toxicity Criteria (version 2.0). ADRs will be faxed to the CTMU based on the following adverse event reporting requirements table:

AGENTS	GRADE				
	1	2	3	4	5
Investigational Agents Expected AE	-----	------	-----	ADR*	ADR
Investigational Agents Unexpected AE	-----	ADR	ADR	24hr/ADR	24hr/ADR
Commercial Agents Expected AE	-----	------	-----	------	------
Commercial Agents Unexpected AE	-----	------	-----	ADR	ADR

ADR = Adverse Drug Reaction Report (within 7 days)
24 hr = Reported to the CTMU within 24 hours
* Grade 4 hematosuppression does not have to be reported for agents known and expected to cause hematosuppression at the dose used.

ADR reports should be submitted via fax within 7 days of the event. These will be forwarded to the NCI and the Coordinating Group within 3 working days. All ADRs should be reported to the local IRB.

For commercially available drugs, written reporting of any increased incidence of a known ADR is also required in addition to grade 4 and 5 toxicities.

All toxicities, including those with separate reporting requirements described above, must be reported on the Toxicity Form. Deaths are required to be reported via the Death Form within 7 days of knowledge of the event.

5.0 EPP SECONDARY MALIGNANCY REPORTING

Investigators are required to report secondary malignancies occurring on or following treatment on NCI-sponsored protocols using commercial drugs. Reporting of cases of secondary AML/MDS is to be performed using the NCI/CTEP Secondary AML/MDS Report Form. This form should be used in place of DCT Adverse Reaction (ADR) Form for reporting this toxicity. All other secondary malignancies should be reported using the form; DCT Adverse Reaction Form. The EPP Notice of Secondary Malignancy must also be completed for all cases of secondary malignancy.

APPENDIX VI

PATHOLOGY PRACTICE GUIDELINES FOR CALGB INVESTIGATORS

PATHOLOGY PRACTICE GUIDELINES* FOR CALGB INVESTIGATORS

CARCINOMA OF THE COLON AND RECTUM

Edited by
Carolyn C. Compton, M.D., PhD.
Chairman, Surgical Pathology Committee

* These guidelines have been developed by the Cancer Committee of the College of American Pathologists (CAP) and have been published in the Archives of Pathology and Laboratory Medicine. They represent CAP policy and have been adapted for CALGB use by the Surgical Pathology Committee of the Pathology Core of the CALGB with the permission of the CAP.

ã 1999 College of American Pathologists. All rights reserved. None of the content of this publication may be reproduced, stored in a retrieval system, or transmitted in any form or by any means (electronic, mechanical, photocopying, recording, or otherwise) without prior written permission of the publisher.

COLON
AND
RECTUM

Pathology Practice Guidelines for CALGB Investigations

Protocol applies to all carcinomas of the colon, and rectum.
Carcinoid tumors, lymphomas, sarcomas and tumors of the vermiform appendix are excluded.

Procedures:

Incisional Biopsy
Excisional Biopsy, Polypectomy
Local Excision (Transanal Disk Excision)
Resection

Author
Carolyn C. Compton, M.D., Ph.D.

Task Force Members and Contributors
Harold E. Bowman, M.D., L. Peter Fielding, M.D., Rodger C. Haggitt, M.D.,
Gerald E. Hanks, M.D., Donald Earl Henson, M.D., Robert V. P. Hutter, M.D., Kenneth D. McClatchey, M.D.,D.D.S., Mary L. Nielsen, M.D., Ali Qizilbash, M.D., Robert R. Rickert, M.D., Leslie H. Sobin, M.D.,
Charles R. Smart, M.D., Steven Sternberg, M.D.,
Sidney Winawer, M.D., David Winchester, M.D.,

ã 1999 College of American Pathologists. All rights reserved. None of the content of this publication may be reproduced, stored in a retrieval system, or transmitted in any form or by any means (electronic, mechanical, photocopying, recording, or otherwise) without prior written permission of the publisher.

COLON AND RECTUM

Incisional (Endoscopic) Biopsy

CLINICAL INFORMATION

Patient identification
Name
Identification number
Age (birth date)
Gender

Responsible physician(s)

Date of procedure

Other clinical information
Relevant history
- previous colon adenoma(s)/carcinoma(s)
- familial adenomatous polyposis syndrome
- hereditary non-polyposis colon cancer syndrome
- familial hamartomatous polyposis syndrome
- inflammatory bowel disease

Relevant findings (e.g., colonoscopic and/or imaging studies)
Clinical diagnosis (e.g., Crohn's disease)
Procedure (e.g., colonoscopic biopsy)
Operative findings
Anatomic site(s) of specimen(s)

MACROSCOPIC EXAMINATION

Specimen
Unfixed/fixed *(specify fixative)*
Number of pieces
Largest dimension of each piece
Description of other tissues *(as appropriate)*

Submit entire specimen for microscopic evaluation

Special studies *(specify)* (e.g., histochemistry, immunohistochemistry, morphometry, DNA analysis [specify type], cytogenetic analysis)

MICROSCOPIC EVALUATION

Tumor (Note A)
Histologic type (Note B)
Histologic grade (Note C)
Extent of invasion, as appropriate

Additional pathologic findings, if present
Colitis
Adenoma
Other(s)

Results/status of special studies *(specify)*

Comments
Correlation with other specimens, as appropriate
Correlation with clinical information, as appropriate

COLON AND RECTUM

Excisional Biopsy, Polypectomy

CLINICAL INFORMATION

Patient identification
Name
Identification number
Age (birth date)
Gender

Responsible physician(s)

Date of procedure

Other clinical information
Relevant history
- previous colon adenoma(s)/carcinoma(s)
- familial adenomatous polyposis syndrome
- hereditary non-polyposis colon cancer syndrome
- familial hamartomatous polyposis syndrome
- inflammatory bowel disease

Clinical diagnosis
Procedure (e.g., polypectomy)
Operative findings
Anatomic site(s) of specimen(s)

MACROSCOPIC EXAMINATION

Specimen
Tissue(s) included
Unfixed/fixed *(specify fixative*)
Number of pieces
Dimensions
Orientation *(if indicated by surgeon)*
Descriptive features (e.g., color, consistency)

Polyp
Configuration (e.g., pedunculated, sessile)
Size (three dimensions)
If pedunculated, length of stalk (margin of stalk may be inked)
Dimension of carcinoma (diameter), if possible

Tissue(s) submitted for microscopic evaluation
Transverse (coronal) section(s) through polyp, include:
- polyp apex and stalk or base in same section, if possible
- carcinoma, point of deepest invasion
- longitudinal section of polyp stalk *(as appropriate)*

Special studies *(specify)* (e.g., histochemistry, immunohistochemistry, morphometry, DNA analysis [specify type], cytogenetic analysis)

MICROSCOPIC EVALUATION

Polyp
Histologic type

Tumor (Carcinoma within polyp)
Histologic type (Note B)
Histologic grade (Notes C and D)
Extent of invasion (Note D):
Blood/lymphatic vessel invasion (Note D)
Distance of carcinoma from margin in mm (Note D)

Results/status of special studies *(specify)*

Comments
Correlation with other specimens, as appropriate
Correlation with clinical information, as appropriate

COLON AND RECTUM

Local Excision (Transanal Disc Excision)

CLINICAL INFORMATION

Patient identification
Name
Identification number
Age (birth date)
Gender

Responsible physician(s)

Date of procedure

Other clinical information
Relevant history
- previous colon adenoma(s)/carcinoma(s)
- familial adenomatous polyposis syndrome
- hereditary non-polyposis colon cancer syndrome
- familial hamartomatous polyposis syndrome
- inflammatory bowel disease

Relevant findings (e.g., colonoscopic and/or imaging studies)
Clinical diagnosis
Procedure (e.g., transanal resection)
Operative findings
Anatomic site(s) of specimen(s)

MACROSCOPIC EXAMINATION

Specimen
Unfixed/fixed *(specify fixative)*
Number of pieces
Dimensions
Orientation of specimen *(if indicated by surgeon)*
Descriptive characteristics (e.g., color, consistency)
Layers of colon/rectum present *(if grossly discernible)*
Results of intraoperative consultation

Tumor (Note A)
Configuration (Note E)
Dimensions (three dimensions)
Distance of tumor edge from closest margin
Estimated depth of invasion
Lesions in noncancerous colon/rectum (e.g., colitis, polyps)

Additional pathologic findings, if present

Tissue(s) submitted for microscopic evaluation
Carcinoma, including:
- points of deepest penetration (at least 3 sections; optimally 5 sections)
- interface with adjacent colon
- margin(s) closest to tumor edge

Frozen section tissue fragment(s)

Special studies *(specify)* (e.g., histochemistry, immunohistochemistry, morphometry, DNA analysis *[specify type]*, cytogenetic analysis)

MICROSCOPIC EVALUATION

Tumor
Histologic type (Note B)
Histologic grade (Note C)
Depth of invasion (Note F)
Blood/lymphatic vessel invasion (Note G)
Perineural invasion (Note G)
Extramural venous invasion (Note H)
Intratumoral or peritumoral lymphocytic response (Note I)
Pattern of growth at tumor periphery (Note J)
- infiltrating border
- pushing border

Margins
Distance of carcinoma from closest mucosal margin and/or deep margin

Additional pathologic findings, if present
Colitis
Dysplasia
Adenomas
Hyperplastic polyps
Other(s)

Results/status of special studies*(specify)*

Comments
Correlation with intraoperative consultation
Correlation with other specimens, as appropriate
Correlation with clinical information, as appropriate

COLON AND RECTUM

Segmental Resection

CLINICAL INFORMATION

Patient identification
Name
Identification number
Age (birth date)
Gender

Responsible physician(s)

Date of procedure

Other clinical information
Relevant history
- previous colon adenoma(s)/carcinoma(s)
- familial adenomatous polyposis syndrome
- hereditary non-polyposis colon cancer syndrome
- familial hamartomatous polyposis syndrome
- inflammatory bowel disease

Relevant findings (e.g., colonoscopic and/or imaging studies)
Clinical diagnosis
Procedure (e.g., right colectomy, transverse colectomy, left colectomy, sigmoidectomy, abdomino-peroneal resection)
Operative findings
Anatomic site(s) of specimen(s) (e.g., cecum, right, transverse, descending, sigmoid colon, or rectum)

MACROSCOPIC EXAMINATION

Specimen
Organ(s)/tissue(s) included
Unfixed/fixed*(specify fixative)*
Number of pieces
Dimensions
Orientation of specimen *(if indicated by surgeon)*
Results of intraoperative consultation

Tumor
Location (Note A)
Configuration (Note E)
Dimensions (three dimensions)
Descriptive characteristics (e.g., color, consistency)
Ulceration/perforation
Distance from margins (Note K)
- proximal
- distal
- radial (soft tissue margin or serosa closest to deepest tumor penetration)

Estimated depth of invasion (Note F):

Lesions in noncancerous colon/rectum (e.g., colitis, other polyps)

Regional lymph nodes (Note F)

Non-regional lymph nodes (Note F)

Metastasis to other organ(s) or structure(s) (Note F)

Colon/rectum uninvolved by tumor

Other tissue(s)/organ(s)

Tissues submitted for microscopic evaluation
Carcinoma, including:
- points of deepest penetration (at least 3 sections; optimally 5 sections)
- interface with adjacent colon/rectum
- visceral serosa overlying tumor

Margins (Note K)
- proximal
- distal
- radial [circumferential] (soft tissue margin closest to deepest tumor penetration)

All lymph nodes (Note F)
Other lesions (e.g., polyps/colitis)
Frozen section tissue fragment(s)

Special studies *(specify)* (e.g., histochemistry, immunohistochemistry, morphometry, DNA analysis [specify type], cytogenetic analysis)

MICROSCOPIC EVALUATION

Tumor
Histologic type (Note B)
Histologic grade (Note C)
Extent of invasion (Note F)
Blood/lymphatic vessel invasion (Note G)
Perineural invasion (Note G)
Extramural venous invasion (Note H)
Intratumoral or peritumoral lymphocytic response (Note I)
Pattern of growth at tumor periphery (Note J)
- infiltrating border
- pushing border

Associated pericolorectal abscess formation *(if present)*
Associated pneumatosis intestinalis *(if present)*

Margins (Note K)
Proximal
Distal
Radial (specify distance of carcinoma from closest radial margin in mm)

Regional lymph nodes (Note F):
Number
Number with metastases

Additional pathologic findings, if present
Inflammatory bowel disease
Dysplasia
Adenomas
Other types of polyps

Distant metastasis*, specify site* (Note F)

Other tissue(s)/organ(s)

Results/status of special studies *(specify)*

Comments
Correlation with intraoperative consultation
Correlation with other specimens, as appropriate
Correlation with clinical information, as appropriate

EXPLANATORY NOTES

A. ANATOMIC SITES
The protocol applies to all carcinomas arising in the colon and rectum.(1)

The colon is divided into four parts: the right (ascending colon), the middle (transverse) colon, the left (descending) colon, and the sigmoid colon. The right colon is subdivided into the cecum (peritoneally located and measuring about 6 x 9 cm) and the ascending colon **(located retroperitoneally** and measuring 15 to 20 cm long). The descending colon, also located retroperitoneally, is 10 to 15 cm in length. The descending colon becomes the sigmoid colon at the origin of the mesosigmoid, and the sigmoid colon becomes the rectum at the termination of the mesosigmoid. The upper third of the rectosigmoid segment is covered by peritoneum on the front and both sides. The middle third is covered by peritoneum only on the anterior surface. The lower third (also known as the rectum or rectal ampulla) has no peritoneal covering.(1)

Tumors located at the border between two subsites of the colon (e.g., cecum and ascending colon) are registered as tumors of the subsite that is more involved. If two subsites are involved to the same extent, the tumor is classified as an "overlapping" lesion. The rectum is defined clinically as the distal large intestine commencing opposite the sacral promontory and ending at the upper border of the anal canal. When measuring below with a rigid sigmoidoscope, it extends 16 cm from the anal verge. A tumor is classified as rectal if its inferior margin lies less than 16 cm from the anal verge or if any part of the tumor is located at least partly within the supply of the superior rectal artery.(2) A tumor is classified as rectosigmoid when differentiation between rectum and sigmoid according to the above guidelines is not possible.(3)

B. HISTOLOGIC TYPES
For consistency in reporting, the histologic classification proposed by the World Health Organization is recommended and is shown below.(4) However, this protocol does not preclude the use of other systems of classification or histologic types.

World Health Organization Classification of Colorectal Carcinoma
Adenocarcinoma *in situ*/severe dysplasia*
Adenocarcinoma
[Medullary carcinoma]**
Mucinous (colloid) adenocarcinoma (>50% mucinous)***
Signet-ring cell carcinoma (>50% signet-ring cells) †
Squamous cell (epidermoid) carcinoma
Adenosquamous carcinoma
Small-cell (oat cell) carcinoma †
Undifferentiated carcinoma †
Other (specify) ‡

* In order to avoid confusion with the term "carcinoma in situ" as it applies to pTis in the TNM staging (see Note F below), it has been recommended that the term "intraepithelial carcinoma" be used to refer to histologically malignant epithelium that does not penetrate the basement membrane (i.e., shows no evidence of stromal [lamina propria] invasion).(5,6)

** Medullary carcinoma has been added to the revised WHO histological classification that will be published in 2000. Medullary carcinoma is a histologic type that is strongly associated with a high degree of microsatellite instability (MSI-H) indicative of loss of normal DNA repair gene function.(6-8) With loss of function of any of the genes involved in the repair of mitosis-associated mistakes in DNA synthesis (i.e., replication errors), mutations in daughter cells are rapidly accumulated. Ultimately, tumors with defective DNA repair acquire inactivating mutations of genes

necessary for complex biologic activities such as metastasis. For this reason, tumors with MSI-H, such as most (if not all) medullary carcinomas, have a favorable prognosis compared to microsatellite stable (MSS) tumors or tumors with low levels of microsatellite instability (MSI-L). Medullary carcinoma may occur either sporadically (7) or in association with the hereditary nonpolyposis colon cancer syndrome (HNPCC).(8) This tumor type is characterized by uniform polygonal tumor cells that exhibit solid growth in nested, organoid, or trabecular patterns and that only focally produce small amounts of mucin. In addition, medullary carcinomas are typically infiltrated by lymphocytes (tumor infiltrating lymphocytes) and have no immunohistochemical evidence of neuroendocrine differentiation.

*** In most previous studies on prognostic factors in colorectal cancer, the impact of genetic status (i.e., loss of DNA repair gene function with microsatellite instability) on the relationship between histologic type and outcome has not been considered. This shortfall is particularly relevant to mucinous carcinoma, a histologic type that is common among tumors with MSI-H. Overall, most mucinous carcinomas are MSS. Thus, it is not surprising that, the prognostic significance of mucinous carcinoma has proven controversial.(6,9) A few studies, largely limited to univariate analyses, have indicated that mucinous adenocarcinoma may be an adverse prognostic factor. Alternatively, mucinous carcinoma has been linked with adverse outcome only when occurring in specific anatomic regions of the bowel (e.g., the rectosigmoid) or in a specific subsets of patients (i.e., those less than 45 years of age). In yet other studies, mucinous carcinoma has been linked to adverse outcome only when mucinous and signet-ring cell carcinomas have been grouped together and compared to typical adenocarcinoma. Data of this type may merely reflect the aggressive biologic behavior of most signet-ring cell tumors. Only one multivariate analysis has shown mucinous carcinoma to be a stage-independent predictor of adverse outcome, but the study was limited to tumors presenting with large bowel obstruction, which itself is an adverse prognostic factor.(for review see ref. 9)

† By convention, signet-ring cell carcinomas, small cell carcinomas and undifferentiated (histologic type) carcinomas are high grade (see below). The only histologic types of colorectal carcinoma that have been shown to have adverse prognostic significance independent of stage are signet-ring cell carcinoma and small-cell carcinoma.(9) Nevertheless, signet ring cell carcinoma may occur in HNPCC in association with MSI-H.(10) Thus, in cases of MSI-H signet ring cell carcinoma, it may be expected that the prognostic significance would be determined by the molecular pathogenesis and would be favorable.

‡ The term “carcinoma, NOS” (not otherwise specified) is not part of the WHO classification.

1.1.1.1.1.1 C. HISTOLOGIC GRADE

A number of grading systems have been suggested in the literature, but a single widely accepted and uniformly employed standard for grading is lacking. Among the suggested grading schemes, the number of grades as well as the criteria for distinguishing among different grades vary markedly. In some systems, grades are defined on the basis of a single microscopic feature, such as the degree of gland formation, and in other systems, a large number of features are included in the evaluation. Irrespective of the complexity of the criteria, however, most systems stratify tumors into three or four grades as follows:

Grade 1 - Well differentiated
Grade 2 - Moderately differentiated
Grade 3- Poorly differentiated
[Grade 4- Undifferentiated]

Variation in the appearance of individual histologic features may vary widely enough to make implementation of the even the simplest grading systems problematic and, ultimately, subjective. Thus, a significant degree of interobserver variability in the grading of colorectal cancer has been shown to exist.(9,11) Nevertheless, despite this variability, histologic grade has repeatedly been

shown by multivariate analysis to be a stage-independent prognostic factor.(9,12,13) In specific, it has been demonstrated that high tumor grade is an adverse prognostic factor. It is noteworthy that in the vast majority of studies documenting the prognostic power of tumor grade (9), the number of grades has been collapsed to produce a two-tiered stratification for data analysis as follows:

Low Grade: Well differentiated and moderately differentiated
High Grade: Poorly differentiated and undifferentiated

In general practice, a two-tiered grading systems would also be expected to greatly reduce interobserver variability, since the widest variations in grading concern the stratification of low grade tumors into well- or moderately-differentiated categories. Pathologic identification of poorly differentiated or undifferentiated tumors is more consistent, and interobserver variability in diagnosing high-grade carcinoma is relatively small. Therefore, in light of its proven prognostic value, relative simplicity, and reproducibility, a two-tiered grading system for colorectal carcinoma (i.e., low grade and high grade) is recommended.(6) The grading should be based on gland formation alone as follows(6):

Low grade = ≥50% gland formation
High grade = <50% gland formation

D. CARCINOMA IN AN ADENOMATOUS POLYP

Colorectal adenomas containing invasive adenocarcinoma that extends through the muscularis mucosae into the submucosa have been defined "malignant polyps." These polyps constitute a form of early (i.e., curable) colorectal carcinoma. The definition of malignant polyps excludes adenomas containing *in situ* carcinoma (also known as intraepithelial carcinoma) and carcinoma either limited to the lamina propria of the polyp mucosa or invading no deeper than the muscularis mucosae (intramucosal carcinoma) because these polyps possess no biological potential for metastasis. The term "malignant polyp" encompasses both polypoid carcinomas in which the entire polyp head is replaced by carcinoma and adenomas with focal malignancy.

Malignant polyps removed by endoscopic polypectomy require evaluation of histologic parameters that have been determined to be significant prognostic factors related to the risk of adverse outcome (i.e., lymph node metastasis or local recurrence from residual malignancy) following polypectomy.(9,14-30) Pathologic features that have been shown to have independent prognostic significance and are crucial for evaluating risk and determining the possible need for further surgical treatment (i.e., segmental colectomy) include:

- histologic grade of the carcinoma
- extent (level) of invasion of the carcinoma within the polyp
- status of the resection margin
- lymphatic/venous vessel involvement

Specifically, an increased risk of adverse outcome has been shown to be associated with:

- high grade (poorly differentiated) carcinoma
- tumor at or less than 1 mm from the resection margin
- presence of lymphatic/venous vessel involvement

E. TUMOR CONFIGURATION

Configurations include exophytic (fungating), endophytic (ulcerative), and diffusely infiltrative (linitis plastica), or annular, but overlap among these types is common. Exophytic is divided into pedunculated and sessile. Overall, gross tumor configuration has no independent influence on prognosis.(6,9) The uncommon linitis plastica type represents a possible exception. It has an

unfavorable prognosis, but its association with adverse outcome is probably related to the underlying histologic type of tumor (signet ring cell carcinoma) rather than the macroscopic configuration itself.

F. TNM AND STAGE GROUPINGS

Surgical resection remains the most effective therapy for colorectal carcinoma, and the best estimation of prognosis is related to the pathologic findings on the resection specimen. The anatomic extent of disease is by far the most important prognostic factor.(12)

The protocol recommends the TNM Staging System of the AJCC /UICC (1,31) but does not preclude the use of other staging systems.

Tumor (T)*

TX	Primary tumor cannot be assessed
TO	No evidence of primary tumor
Tis	Carcinoma *in situ* - intraepithelial or invasion of the lamina propria or muscularis mucosae**
T1	Tumor invades the submucosa
T2	Tumor invades the muscularis propria
T3	Tumor invades through the muscularis propria into the subserosa or into the nonperitonealized pericolic or perirectal tissues***<br>pT3a - minimal invasion: <1 mm beyond the border of the muscularis propria<br>pT3b - slight invasion: 1-5 mm beyond the border of the muscularis propria<br>pT3c - moderate invasion :>5-15 mm beyond the border of the muscularis propria<br>pT3d - extensive invasion: >15 mm beyond the border of the muscularis propria
T4	Tumor directly invades other organs or structures[†] (T4a) or perforates the visceral peritoneum[‡] (T4b)

* The designation "T" refers to the first resection of a primary tumor. The symbol "pT" refers to the pathologic classification of the TNM, as opposed to the clinical classification. Pathologic classification is based on gross and microscopic examination. pT entails a resection of the primary tumor or biopsy adequate to evaluate the highest pT category; pN entails removal of nodes adequate to validate lymph node metastasis; and pM implies microscopic examination of distant lesions. Clinical classification (cTNM) is usually carried out by the referring physician before treatment during initial evaluation of the patient or when pathologic classification is not possible and is typically based on information obtained by physical examination, serologic analyses, imaging studies, etc.(1)

Tumor <u>remaining in a patient after primary therapy</u> (e.g., surgical resection) is categorized by a system known as R classification, shown below.(1,6,12) For example, this classification may be used by the surgeon to indicate the known or assumed status of the completeness of the surgical resection. For the pathologist, the R classification is relevant only to the margins of surgical resection specimens. That is, tumor involving the proximal, distal or radial (circumferential) resection margin [see below Note K below] on pathologic examination may be assumed to correspond to residual tumor in patient and classified as to whether the involvement is macroscopic or microscopic.(6)

RX	Presence of residual tumor cannot be assessed
R0	No residual tumor
R1	Microscopic residual tumor
R2	Macroscopic residual tumor.

Tumor <u>remaining in a resection specimen following previous (neoadjuvant) treatment</u> of any type (radiation therapy alone, chemotherapy therapy alone, or any combined modality treatment) is codified by the TNM using a prescript "y" to indicate the post-treatment status of the tumor (e.g., ypT1).(1,6) For many therapies, the classification of residual disease has been shown to be a strong predictor of postoperative outcome. In addition, the ypTNM classification provides a standardized framework for the collection of data needed to accurately evaluate new neoadjuvant therapies.

Tumor that is locally recurrent after a documented disease-free interval following surgical resection is classified according to the TNM categories but modified with the prefix "r" (e.g., rpT1). By convention, the recurrent tumor is topographically assigned to the proximal segment of the anastomosis unless that segment is small intestine.(1,3)

**For colorectal carcinomas, "carcinoma *in situ*" (Tis) as a staging term includes cancer cells confined within the glandular basement membrane (intraepithelial carcinoma) or invasive into the mucosal lamina propria, up to but not through the muscularis mucosae (intramucosal carcinoma). This may be confusing because, in all other organ systems, the term "carcinoma in situ" is used to refer exclusively to malignancy that does not invade the underlying stroma. Therefore, for colorectal cancer, the terms "intraepithelial carcinoma" and "intramucosal carcinoma" are recommended as descriptive terms to subclassify pTis and to clarify the status of the tumor.(5,6) Tumor extension through the muscularis mucosae into the submucosa is classified as T1. Some pathologists classify intraepithelial carcinoma as *severe or high-grade dysplasia*, especially in cases of inflammatory bowel disease.

***The extent of perimuscular invasion has been reported to influence prognosis, regardless of whether regional lymph node metastasis is present. Thus, an optional expansion pT3 has been proposed.(3) Extramural extension >5mm has been shown to be the critical subdivision associated with adverse outcome in most studies. Thus, a simpler subdivision, based on extension of ≤5mm vs. >5mm (i.e., pT3a,b vs. pT3c,d), may be justified (3). Extension of the tumor within lymphatics or veins does not count as local spread of tumor as defined by the T classification.(3)

† Direct invasion of other organs or structures includes invasion of other segments of colorectum by way of the serosa or mesocolon for example, invasion of the sigmoid colon by carcinoma of the cecum. Intramural extension of tumor from one subsite (segment) of the large intestine into an adjacent subsite or into the ileum (e.g.. for a cecal carcinoma) or anal canal (e.g., for a rectal carcinoma) does not affect the pT classification.(3)

‡ Perforation of carcinoma through a peritonealized surface of the colon is classified as T4b.(3) Subdivision of T4 into T4a and b is justified because a number of large studies that have evaluated serosal penetration as an independent prognostic variable have demonstrated by multivariate analysis that it has a strong negative impact on prognosis.(32-35) In specific, it has been shown that the frequency of distant metastasis is higher in cases with perforation of the visceral peritoneum compared to cases with direct invasion of adjacent organs or structures without perforation of the visceral peritoneum (occurring in about 50% and 30% of cases, respectively).(3) Furthermore, the median survival time following surgical resection for cure has been shown to be shorter for patients with pT4b tumors compared to those with pT4a tumors (with or without distant metastasis) as follows:(3)

	5-Year Survival Rate	Median Survival Time (mo.)
pT4a,M0	49%	58.2
pT4b,M0	43%	46.2
pT4a,M1	12%	22.7
pT4b,M1	0%	15.5

A study by Shepherd *et al*. (34) has suggested that the prognostic power of local peritoneal involvement in curative resections may supersede that of either local extent of tumor (T category) or regional lymph node status (N category). However, serosal penetration is often difficult to assess histopathologically and may be underdiagnosed. Documentation of peritoneal involvement by tumor demands meticulous pathologic analysis and may require extensive sampling and/or serial sectioning and can be missed on routine histopathologic examination. It has been shown that cytologic examination of serosal scrapings reveals malignant cells in as many as 26% of tumor specimens categorized as pT3 by histologic examination alone.(34,36) In addition, the histopathologic findings associated with peritoneal penetration are heterogeneous and standard guidelines for their diagnostic interpretation are lacking. Therefore, interobserver variability in the diagnosis of peritoneal penetration may be substantial, and since most pathologists tend to err on the side of conservative interpretation, underdiagnosis is likely for this reason as well.

Shepherd *et al*. (34) analyzed the spectrum of microscopic features that may be seen with local peritoneal involvement by tumor, and defined three types of local peritoneal involvement as follows:

1) a mesothelial inflammatory and/or hyperplastic reaction with tumor close to, but not at, the serosal surface;
2) tumor present at the serosal surface with inflammatory reaction, mesothelial hyperplasia, and/or erosion/ulceration;
3) free tumor cells on the serosal surface (in the peritoneum) with underlying ulceration of the visceral peritoneum.

All three types of local peritoneal involvement were associated with decreased survival, whereas tumor well clear of the serosal had no independent adverse effect on prognosis. Therefore, it is recommended that in that diagnosis of T4b encompass the three types of serosal involvement detailed above.(6)

Regional Lymph Nodes (N)*

NX	Regional lymph nodes cannot be assessed
NO	No regional lymph node metastasis
N1	Metastasis in 1 to 3 lymph nodes
N2	Metastasis in 4 or more lymph nodes**,***

*The regional lymph nodes for the anatomical subsites of the large intestine are as follows:
Cecum - anterior cecal, posterior cecal, ileocolic, right colic
Ascending colon - ileocolic, right colic, middle colic
Hepatic flexure - middle colic, right colic
Transverse colon - middle colic
Splenic flexure -middle colic, left colic, inferior mesenteric
Descending colon - left colic, inferior mesenteric, sigmoid
Sigmoid colon - inferior mesenteric, superior rectal sigmoidal, sigmoid mesenteric
Rectosigmoid - perirectal, left colic, sigmoid mesenteric, sigmoidal, inferior mesenteric, superior rectal, middle rectal
Rectum - perirectal, sigmoid mesenteric, inferior mesenteric, lateral sacral, presacral, internal iliac, sacral promontory, superior rectal, middle rectal, inferior rectal

**Nodes along the sigmoid arteries are considered pericolic nodes, and their involvement is classified as N1 or N2 according to the number involved.

***Perirectal lymph nodes include the mesorectal (paraproctal), lateral sacral, presacral, sacral promontory (Gerota), middle rectal (hemorrhoidal), and inferior rectal (hemorrhoidal) nodes. Metastasis in the external iliac or common iliac nodes is classified as distant metastasis.(3)

Important notes on Lymph Nodes:
Submission of lymph nodes for microscopic examination: All grossly negative or equivocal lymph nodes are to be submitted entirely.(6) Grossly positive lymph nodes may be partially submitted for microscopic confirmation of metastasis.

It has been shown that 12 to 15 negative lymph nodes predict for regional node negativity. Therefore, if fewer than 12 nodes are found, additional techniques (i.e., visual enhancement techniques) should be considered.(6) If fewer than 12 nodes are found after the use of visual enhancement techniques, this should be communicated in the pathology report. The pathology report should clearly state the total number of lymph nodes examined and the total number involved by metastases. Data are insufficient to recommend routine use of tissue levels or special/ancillary techniques.(6)

Non-regional lymph nodes: For microscopic examination of lymph nodes in large resection specimens, lymph nodes must be designated as regional vs. non-regional, according to the anatomic location of the tumor. Metastasis to non-regional lymph nodes is classified as distant metastasis and designated as M1 (see below).

Lymph nodes replaced by tumor: A tumor nodule >3 mm in diameter in the perirectal or pericolonic adipose tissue without histologic evidence of residual lymph node in the nodule is classified as regional perirectal/pericolonic node metastasis. However, a tumor nodule ≤3 mm in diameter is classified in the T category as discontinuous extension

(i.e., T3) (1,3). Multiple metastatic foci seen microscopically only in the pericolonic fat should be considered as metastasis in a single lymph node for classification (1).

Micrometastasis: Routine assessment of regional lymph node metastasis is limited to the use of conventional pathologic techniques (gross assessment and histologic examination). A solitary focus of tumor in a single lymph node that is visualized by routine histologic examination and measures ≤2.0 mm may be defined as a “micrometastasis”.(6) The biologic significance of micrometastatic disease (either a single focus in a single node, multiple foci within a single node, or micrometastatic involvement of multiple nodes) as yet is unproven. Pending definitive studies, it is recommended that micrometastases be classified as pN1 but reported with a note explaining that the biologic significance is unknown. The number of lymph nodes involved by micrometastases should be clearly stated.(6)

The biologic significance of metastasis detected only by special studies (e.g., immunohistochemical staining or molecular analysis) is also unproven at present. It is recommended that metastasis diagnosed on special studies alone also be reported with a note explaining the unknown significance of the findings, but, in contrast to histologically identified micrometastases, be classified as pN0.(6) Currently, the data are insufficient to recommend either the routine examination of multiple tissue levels of paraffin blocks or the use of special/ancillary techniques such as immunohistochemistry for epithelial and/or tumor-associated antigens (e.g., cytokeratin, carcinoembryonic antigen, etc.) or polymerase chain reaction (PCR) techniques to identify tumor RNA/DNA.(6)

Distant Metastasis (M)

MX	Presence of distant metastasis cannot be assessed
M0	No distant metastasis
M1	Distant metastasis*

*Seeding of abdominal organs is classified as M1.

**TNM Stage Groupings**				**Modified Astler-Coller Stage**	
Stage 0	Tis	N0	M0	Stage A	
Stage I	T1	N0	M0	N/A	
		T2	N0	M0	Stage B1
Stage II T3	N0	M0		Stage B2	
		T4	N0	M0	Stage B3
Stage III Any T	N1	M0		Stage C1 (T2); C2 (T3); C3 (T4)	
		Any T	N2	M0	Stage C1 (T2); C2 (T3); C3 (T4)
Stage IVAny T	Any N	M1		Stage D	

G. LYMPHATIC (THIN-WALLED) VESSEL AND PERINEURAL INVASION

In several studies, both lymphatic invasion and perineural invasion have been shown by multivariate analysis to be independent indicators of poor prognosis.(13,32,30,37,38) The prognostic significance, if any, of the anatomic location of these structures is not defined. Furthermore, it is not always possible to distinguish lymphatic vessels from post-capillary venules since both are small, thin-walled structures. Thus, the presence or absence of tumor invasion of small, thin-walled vessels should be reported in all cases and its anatomic location within the colonic wall noted.(6)

H. VENOUS INVASION

Extramural venous invasion has been demonstrated by multivariate analysis to be an independent adverse prognostic factor.(13,32,38-41) Invasion of extramural veins, in particular, has been shown to be an independent indicator of unfavorable outcome and increased risk of occurrence of hepatic metastasis.(40,41) It has been shown that the submission of 5 or more blocks of tumor significantly enhances the likelihood of finding extramural venous invasion when it exists and reduces false negativity due to sampling error.(42)

The significance of intramural venous invasion is less clear, because data specific to this issue are lacking. Nevertheless, it is recommended that the presence or absence of venous invasion and its anatomic location should be reported in all cases.(6)

I. LYMPHOCYTIC RESPONSE TO TUMOR

A conspicuous lymphoid reaction at the leading edge of invasive tumor or the presence of lymphoid aggregates in the surrounding tissues (muscularis external and pericolonic or perirectal fat) have both been shown in small studies to be independent favorable prognostic factors.(11,41,43-45) Intratumoral lymphocytic infiltrates are closely associated with microsatellite instability and medullary architecture (see above) and should be distinguished from peritumoral infiltrates. Only moderate and high density intratumoral lymphocytes (approximately 4 or more per high-power field) should be considered significant.(6) Reporting of host lymphoid response is optional. If reported, distinction should be made between peritumoral and intratumoral lymphoid infiltrates.

J. TUMOR PERIPHERY: GROWTH PATTERN

The growth pattern of the tumor at the advancing edge has been shown to have prognostic significance independent of stage and may predict liver metastasis.(46-52) Specifically, an infiltrating pattern of growth at the tumor border as opposed to a pushing border is an adverse prognostic factor.

Infiltrating borders have been defined as follows:(47)

<u>Naked Eye Examination</u>

- Inability to define limits of invasive border of tumor and/or
- Inability to resolve host tissue from malignant tissue

<u>Microscopic Examination of Slides</u>

- "Streaming dissection" of muscularis propria (dissection of tumor through the full thickness of the muscularis propria without stromal response) and/or
- Dissection of mesenteric adipose tissue by small glands or irregular clusters or cords of cells and/or
- Perineural invasion

Irregular growth at the tumor periphery has also been referred to as "focal dedifferentiation" and "tumor budding" and defined as microscopic clusters of undifferentiated cancer cells just ahead of the invasive front of the tumor.

K. MARGINS

It may be helpful to mark the margin(s) closest to the tumor with ink. Margins marked by ink should be designated in the macroscopic description. Margins include the proximal, distal, and radial margins. The radial margin represents the adventitial soft tissue margin closest to the deepest penetration of tumor. For all segments of the large intestine that are either incompletely encased (ascending colon, descending colon, sigmoid colon, upper rectum) or not encased (lower rectum) by peritoneum, the radial margin is created by blunt dissection of the retroperitoneal or subperitoneal aspect, respectively, at operation.

The radial margin has been demonstrated to be of importance in relation to risk of local recurrence after surgical resection of the rectal carcinomas.(53-55) Multivariate analysis has suggested that tumor involvement of the radial margin is the most critical factor in predicting local recurrence in rectal cancer.(53-55) For this reason, routine assessment of the radial margin is suggested in all colorectal cancers, and measurement of the distance from the tumor to the radial margin, representing the “surgical clearance” around the tumor is suggested.(56) For segments of the colon that are completely encased by a peritonealized (serosal) surface (e.g., transverse colon), the only radial margin is the mesenteric resection margin, and it is relevant when the point of deepest penetration of the tumor is on the mesenteric aspect of the colon and extends to this margin with or without penetrating the serosal surface. For those tumors limited to an antimesenteric peritonealized aspect of the bowel, the radial margin is not relevant.

Because of its association with local recurrence, involvement of the radial margin has implications for adjuvant therapy. Whether the primary tumor is T3 (without serosal penetration) or T4b (with serosal penetration), resection is considered

complete only if all surgical margins are negative including the radial margin. That is, whether or not the tumor penetrates a serosal surface, resection is considered complete if the resection margins (proximal, distal and radial) do not contain tumor. If a radial margin is involved by tumor, adjuvant therapy (e.g., local radiation) may be appropriate.

Sections to evaluate the proximal and distal resection margins can be obtained in two orientations: 1) *en face* sections parallel to the margin or 2) longitudinal sections perpendicular to the margin. Depending on the closeness of the tumor to the margin, select the orientation(s) that best demonstrate the status of the margin. The distance from the tumor edge to the closest resection margin(s) should be measured. In cases of carcinoma arising in a background of inflammatory bowel disease, proximal and distal resection margins should be evaluated for dysplasia and active inflammation.

REFERENCES

1. Fleming ID, Cooper JS, Henson DE, et al. eds, AJCC Manual for Staging of Cancer, 5th ed, Lippincott Raven, Philadelphia, 1997.
2. Fielding LP, Arsenault PA, Chapuis PH, Dent O, Gatright B, Hardcastle JD, Hermanek P, Jass JR, Newland RC. Clinicopathological staging for colorectal cancer: An International Documentation System (IDS) and an International Comprehensive Terminology (ICAT). J Gastroenterol Hepatol. 1991;6:325-344.
3. Hermanek P, Henson DE, Hutter RVP, Sobin LH. TNM Supplement. New York, NY: Springer-Verlag NY Inc. 1993.
4. Jass JR, Sobin LH. Histological Typing of Intestinal Tumours: World Health Organization. 2nd ed. New York, NY: Springer-Verlag NY Inc; 1989.
5. Compton CC, Fenoglio-Preiser CM, Pettigrew N, Fielding LP. American Joint Committee on Cancer Prognostic Factors Consensus Conference: Colorectal Working Group. Cancer, in press.
6. Compton CC, Fielding LP, Burgart LJ, et al. Prognostic Factors in Colorectal Cancer: College of American Pathologists Consensus Statement 1999. Arch Pathol Lab Med, in press.
7. Kim H, Jen J, Vogelstein B, Hamilton SR. Clinical and pathological characteristics of sporadic colorectal carcinomas with DNA replication errors in microsatellite sequences. Am J Pathol 1994;145:148-156.
8. Jass JR, Smyrk TC, Stewart SM, Lane MR, Lanspa SJ, Lynch HT. Pathology of Hereditary Non-polyposis Colon Cancer. Anticancer Research 1994;14:1631-1634.
9. Compton CC. Pathology report in colon cancer: What is prognostically important? Dig Dis 1999;17:67-79.
10. Symrk TC. Colon cancer connections. Cancer syndrome meets molecular biology meets histopathology. Am J Pathol 1994;145:1-6.
11. *Jass JR, Cuzick J, Bussey HJR, Morson BC, Northover JMA, Todd IP. The grading of rectal cancer: historical perspectives and a multivariate analysis of 447 cases. Histopathology 1986;10:437-439.*
12. Hermanek P, Sobin LH. Colorectal Carcinoma. In: Hermanek P, Gospodarowicz MK, Henson DE, Hutter RVP, Sobin LH, eds. Prognostic Factors in Cancer. New York, NY: Springer-Verlag NY Inc; 1995.
13. Hermanek P, Guggenmoos-Holzmann I, Gall FP. Prognostic factors in rectal carcinoma. A contribution to the further development of tumor classification. Dis Colon Rectum 1989;32:593-599.

14. Cooper HS. Surgical pathology of endoscopically removed malignant polyps of the colon and rectum. Am J Surg Pathol 1983;7:613-623.

15. Lipper S, Kahn LB, Ackerman LV. The significance of microscopic invasive cancer in endoscopically removed polyps of the large bowel. Cancer 1983;52:1691-1699.

16. Morson BC, Whiteway JE, Jones EA, Macrae FA, Williams CB. Histopathology and prognosis of malignant colorectal polyps treated by endoscopic polypectomy. Gut 1984;25:437-444.

17. Haggitt RC, Glotzbach RE, Soffer EE, Wruble LD. Prognostic factors in colorectal carcinomas arising in adenomas: implications for lesions removed by endoscopic polypectomy. Gastroenterology 1985;89:328-336.

18. Cranley JP, Petras RE, Carey WD, Paradis K, Sivak MV. When is endoscopic polypectomy adequate therapy for colonic polyps containing invasive carcinoma? Gastroenterology 1986:91:419-427.

19. Wilcox GM, Anderson PB, Colacchio TA. Early invasive carcinoma in colonic polyps. A review of the literature with emphasis on the assessment of the risk of metastasis. Cancer 1986;57:160- 171.

20. Richards WO, Webb WA, Morris SJ, et al. Patient management after endoscopic removal of the cancerous colon adenoma. Ann Surg 1987:205:665-672.

21. Wilcox GM, Beck JR. Early invasive cancer in adenomatous colonic polyps (malignant polyps). Evaluation of the therapeutic options by decision analysis. Gastroenterology 1987;92:1159-1168.

22. Christie JP. Polypectomy or colectomy? Management of 106 consecutively encountered colorectal polyps. Am Surg 1988;54:93-99.

23. Cooper HS. The role of the pathologist in the management of patients with endoscopically removed malignant colorectal polyps. Pathol Annu (Part 1) 1988;23:25-43.

24. Coverlizza S, Risio M, Ferrari, A, Fenoglio-Preiser CM, Rossini FP. Colorectal adenomas containing invasive carcinoma. Pathologic assessment of lymph node metastatic potential. Cancer 1989;64:1937-1947.

25. Muller S, Chesner IM, Egan MJ, et al. Significance of venous and lymphatic invasion in malignant polyps of the colon and rectum. Gut 1989;30:1385-1391.

26. Geraghty JM, Williams CB, Talbot IC. Malignant colorectal polyps: venous invasion and successful treatment by endoscopic polypectomy. Gut 1991;32:774-778.

27. Nivatvongs S, Rojanasakul A, Reiman HM, et al. The risk of lymph node metastasis in colorectal polyps with invasive adenocarcinoma. Dis Colon Rectum 1991;34:323-328.

28. Kyzer S, Bégin LR, Gordon PH, Mitmaker B. The care of patients with colorectal polyps that contain invasive adenocarcinoma. Cancer 1992;70:2044-2050.

29. Cooper HS, Deppisch LM, Gourley WK, et al. Endoscopically removed malignant colorectal polyps: clinicopathologic correlations. Gastroenterology 1995;108:1657-1665.

30. Volk EE, Goldblum JR, Petras RE, Carey WD, Fazio VW. Management and outcome of patients with invasive carcinoma arising in colorectal polyps. Gastroenterology 1995;109:1801-1807.

31. Sobin LH, Wittekind C, eds. TNM Classification of Malignant Tumours: International Union Against Cancer. 5th ed., New York, NY: Wiley , 1997.

32. Chapuis PH, Dent OF, Fisher R, et al. A multivariate analysis of clinical and pathological variables in prognosis after resection of large bowel cancer. Br J Surg 1985;72:698-702.

33. Newland R, Dent O, Lyttle M, Chapuis PH, Bokey EL. Pathologic determinants of survival associated with colorectal cancer with lymph node metastases. A multivariate analysis of 579 patients. Cancer 1994;73:2076-2082.

34. Shepherd N, Baxter K, Love S. The prognostic importance of peritoneal involvement in colonic cancer: A prospective evaluation. Gastroenterology 1997;112:1096-1102.

35. Tominaga T, Sakabe T, Koyama Y, et al. Prognostic factors for patients with colon or rectal carcinoma treated with resection only. Five-year follow-up report. Cancer 1996;78:403-408.

36. Zeng Z, Cohen AM, Hajdu S, Sternberg SS, Sigurdson ER, Enker W. Serosal cytologic study to determine free mesothelial penetration of intraperitoneal colon cancer. Cancer 1992;70:737-740.

37. Knudsen JB, Nilsson T, Sprechler M, Johansen A, Christensen N. Venous and nerve invasion as prognostic factors in postoperative survival of patients with resectable cancer of the rectum. Dis Colon Rectum 1983;26:613-617.

38. Michelassi F, Block GE, Vannucci L, Montag A, Chappell R. A 5- to 21-year follow-up and analysis of 250 patients with rectal adenocarcinoma. Ann Surg 1988;208:379-387.

39. Minsky BD, Mies C, Rich TA, Recht A. Lymphatic vessel invasion is an independent prognostic factor for survival in colorectal cancer. Int J Radiat Oncol Biol Phys 1989;17:311-318.

40. Talbot IC, Ritchie S, Leighton MH, Hughes AO, Bussey HJR, Morson BC. The clinical significance of invasion of veins in rectal cancer. Br J Surg 1980;67:439-442.

41. Talbot IC, Ritchie S, Leighton M, Hughes AO, Bussey H, Morson BC. Invasion of veins by carcinoma of the rectum. Method of detection, histologic features, and significance. Histopathology 1981;5:141-163.

42. Blenkinsopp W, Stewart-Brown S, Blesovsky L, Kearney G, Fielding LP. Histopathology reporting in large bowel cancer. J Clin Pathol 1981;34:509-513.

43. Harrison JC, Dean PJ, El-Zekey F, Vander Zwaag R. From Dukes through Jass: Pathological prognostic indicators in rectal cancer. Hum Pathol 1994;25:498-505.

44. Graham DM, Appelman HD. Crohn's-like lymphoid reaction and colorectal carcinoma: A potential histologic prognosticator. Mod Pathol 1990;3:332-335.

45. Harrison JC, Dean PJ, El-Zeky F, Vander Zwaag R. Impact of Crohn's-like lymphoid reaction on staging of right-sided colon cancer: Results of multivariate analysis. Hum Pathol 1995;26:31-38.

46. Carlon CA, Fabris G, Arslan-Pagnini C, Pluchinotta AM, Chinelli E, Carniato S. Prognostic correlations of operable carcinoma of the rectum. Dis Colon Rectum 1985;28:47-50.

47. Jass JR, Atkin WS, Cuzick J, et al. The grading of rectal cancer: historical perspectives and a multivariate analysis of 447 cases. Histopathology 1986;10:437-459.

48. Jass JR, Love SB, Northover JMA. A new prognostic classification of rectal cancer. Lancet 1987;1:1303-1306.

49. Shepherd NA, Saraga EP, Love SB, Jass JR. Prognostic factors in colonic cancer. Histopathology 1989;14:613-620.

50. Hase K, Shatney C, Johnson D, Trollope M, Vierra M. Prognostic value of tumor "budding" in patients with colorectal cancer. Dis Colon Rectum 1993;36:627-35.

51. Roncucci L, Fante R, Losi L, et al. Survival for colon and rectal cancer in a population-based cancer registry. Eur J Cancer 1996;32A:295-302.

52. Ono M, Sakamoto M, Ino Y, et al. Cancer cell morphology at the invasive front and expression of cell adhesion-related carbohydrate in the primary lesion of patients with colorectal carcinoma with liver metastasis. Cancer 1996;78:1179-1186.

53. Quirke P, Durdy P, Dixon MF, Williams NS. Local recurrence of rectal adenocarcinoma due to inadequate surgical resection. Lancet 1986;2:996-999.

54. Quirke P, Scott N. The pathologists role in the assessment of local recurrence in rectal carcinoma. Surg Oncol Clin N Amer 1992;3:1-17.

55. Adam IJ, Mohamdee MO, Martin IG, et al. Role of the circumferential margin involvement in the local recurrence of rectal cancer. Lancet 1994;344:707-711.

56. Chan KW, Boey J, Wong SKC. A method of reporting radial invasion and surgical clearance of rectal carcinoma. Histopathology 1985;9:1319-1327.

BIBLIOGRAPHY

Andreola S, Leo E, Belli F, et al. Manual dissection of adenocarcinoma of the lower third of the rectum for detection of lymph node metastases smaller than 5 mm. Cancer 1996;77:607-612.

Bosman FT. Prognostic value of pathological characteristics of colorectal cancer. *Eur J Cancer* 1995;31A:1216-1221.

Böttger TC, Potratz D, Stöckle M, Wellek S, Klupp J, Juninger T. Prognostic value of DNA analysis in colorectal carcinoma. Cancer 1993;72:3579-3587.

Carriaga MT, Henson DE. The histologic grading of cancer. Cancer (Suppl) 1995;75;406-421.

Cohen AM, Tremiterra S, Cancela F, Thaler HT, Sigurdson ER. Prognosis of node-positive colon cancer. Cancer 1991;67:1859-1861.

Crissman JD, Zarbo RJ, Ma CK, Visscher DW. Histologic parameters and DNA analysis in colorectal adenocarcinomas. Pathol Ann 1989;24:103-147.

Emdin SO, Stenling R, Roos G. Prognostic value of DNA content in colorectal carcinoma. Cancer 1987;60:1282-1287.

Fielding LP, Arsenault PA, Chapuis P, et al. Clinicopathological staging for colorectal cancer. J Gastroenterol Hepatol 1991;6:325-344.

Fielding LP, Phillips RKS, Fry JS, Hittinger R. Prediction of outcome after curative resection for large bowel cancer. Lancet 1986;2:904-907.

Griffin MR, Bergstralh EJ, Coffey RJ, Beart RW Jr, Melton LJ III. Predictors of survival after curative resection of carcinoma of the colon and rectum. Cancer 1987;60:2318-2324.

Hermanek P, Giecil J, Dworak O. Two programs for examination of regional lymph nodes in colorectal carcinoma with regard to new pN classification. Pathol Res Pract 1989;185:867-873.

Hutter RVP, Sobin LH. A universal staging system for cancer of the colon and rectum: Let there be light. Arch Pathol Lab Med 1986;110:367-368.

Krasna MJ, Flancbaum L, Cody RP, Shneibaum S, Ben Ari G. Vascular and neural invasion in colorectal carcinoma. Cancer 1988;61:1018-1023.

Minksy BD, Mies C, Reclit A, Richt A, Chalfey JT. Resectable adenocarcinoma of the rectosigmoid and rectum. I: Patterns of failure and survival. Cancer 1988;61:1408-1416.

Minsky BD, Mies C, Recht A, Rich TA, Chaffey JT. Resectable adenocarcinoma of the rectosigmoid and rectum. Cancer 1988;61:1417-1424.

Minsky BD, Mies C, Rich TA, Recht A, Chaffey JT. Colloid carcinoma of the colon and rectum. Cancer 1987;60:3103-3112.

Morson BC. Histopathology reporting in large bowel cancer *(Editorial).* BMJ 1981;283:1493-1494.

Nacopoulou L, Azaris P, Paracharalampous N, Davaris P. Prognostic significance of histologic host response in cancer of the large bowel. Cancer 1981;47:930-936.

Patt DJ, Brynes RK, Vardiman JW, Coppleson LW. Mesocolic lymph node histology is an important prognostic indicator for patients with carcinoma of the sigmoid colon: An immunomorphologicstudy. Cancer 1975;35:1388-1396.

Phillips RKS, Hittinger R, Blesovsky L, Fry JS, Fielding LP. Large bowel cancer: Surgical pathology and its relationship to survival. Br J Surg 1984;71:604-610.

Qizilbash AH. Pathologic studies in colorectal cancer. Pathol Annu 1982;17:1-46.

Steinberg SM, Barkin IS, Kaplan RS, Stablein DM. Prognostic indicators of colon tumors. Cancer 1986;57:1866-1870.

Steinberg SM, Berwick KW, Stablein DM. Importance of tumor pathology and morphology in patients with surgically resected colon cancer. Cancer 1986;58:1340-1345.

Tsakraklides F, Wanebo HJ, Sternberg SS, Stearns M, Good RA. Prognostic evaluation of regional lymph node morphology in colorectal cancer. Am J Surg 1975;129:174-180.

Watt AG, Flouse AK. Colonic carcinoma. Cancer 1978;41:279-282.

Wiggers T, Arends JW, Schutte B, Volovics L, Bosman FT. A multivariate analysis of pathologic prognostic indicators in large bowel cancer. Cancer 1988;61:386-395.

Zarbo RJ. Interinstitutional assessment of colorectal carcinoma surgical pathology report adequacy. A College of American Pathologists Q-Probes study of practice patterns from 532 laboratories and 15,940 reports. *Arch Pathol Lab Med* 1992;116:111:1113-1119.

APPENDIX VII

PATHOLOGIC PROGNOSTIC FACTORS IN STAGE II COLON CARCINOMA

CALGB 89803
Appendix VII

PATHOLOGIC PROGNOSTIC FACTORS IN STAGE III COLON CARCINOMA

Study Investigator:

Carolyn Compton, M.D., Ph.D.
Massachusetts General Hospital

1.0 PATHOLOGIC PROGNOSTIC FACTORS

It is self-evident that, compared to data derived from additional assays, prognostic information that can be derived directly from standard histologic sections of a tumor is of the greatest cost-benefit to the patient. In the current era of cost containment, it is essential that the prognostic and/or predictive significance of pathologic prognostic factors be defined. In colorectal cancer, pathologic features were among the first variables studied with reference to biologic behavior and have been the subject of numerous studies. Unfortunately, an understanding of the biological significance of these features is still lacking. Previous studies have been limited by small study size, retrospective analysis, lack of defined guidelines for the assessment of individual pathologic features, and lack of quality control. Besides employing different approaches to the analysis of pathologic features, these studies have employed different staging systems. Since tumor stage is the strongest prognostic indicator in colorectal cancer, all other factors of possible importance must be analyzed against staging parameters in order to demonstrate independent prognostic value. Overall, therefore, the prognostic significance of many of the most familiar and widely reported pathologic variables in colorectal cancer is unclear and remains to be defined by prospective studies with the statistical power of the proposed investigations (1). The studies described herein have the additional advantages of the use of an internationally accepted staging system (the TNM staging system of the American Joint Committee on Cancer and the International Union Against Cancer) (2) and standards of pathological evaluation proposed by the College of American Pathologists (3,4), which will be provided to all participating pathologists by the CALGB. The evaluations will be quality controlled by an expert gastrointestinal pathologist under the auspices of the Pathology Committee of the CALGB.

In this study, pathology reports will be submitted along with the representative blocks of tumor from each patient enrolled to the CALGB Pathology Coordinating Office (PCO). Three H&E slides (top, middle and bottom) will be prepared from each block of tumor tissue submitted. These are prepared routinely, according to PCO standard practice, for purposes of quality control in order to: 1) check the accuracy of the diagnosis; 2) check that the tumor in the block submitted is representative of the tumor described in the pathology report; and 3) check that representative tumor is present in all three of the tissue levels cut from the blocks (which, in turn, guarantees that all unstained sections in between also contain representative tumor for correlative science studies). Dr. Compton, a gastrointestinal pathologist who is the designated study pathologist from the CALGB Pathology Committee and Colon Pathology Cadre, will read these slides for quality control and assessment of tumor grade and proliferative index. Other histopathologic features of possible prognostic significance will be assessed by the submitting pathologist according to the CALGB Pathology Data form (see attachment). The individual pathologic features to be assessed in this study are detailed below.

2.0 PATHOLOGIC FEATURES TO BE EVALUATED FROM H&E SLIDES OF SUBMITTED TUMOR BLOCKS

2.1 Tumor Grade

A number of grading systems for colorectal carcinoma have been proposed in the literature, but a single widely accepted, uniformly employed standard for grading is lacking. Among the suggested grading schemas, both the number of grades and the criteria for distinguishing among them vary markedly. In some systems, grade is defined on the basis of a single microscopic feature, such as the degree of gland formation. In other systems, a large number of histopathologic features are included in the evaluation. Irrespective of the complexity of the criteria, however, most systems stratify tumors into three or four grades as follows:

Grade 1 - Well differentiated

Grade 2 - Moderately differentiated

Grade 3- Poorly differentiated

(Grade 4- Undifferentiated)

Ultimately, however, histologic grading is largely subjective and is associated with a significant degree of interobserver variability (5,6).

Despite these issues, histologic grade has repeatedly been shown to be an independent prognostic factor on multivariate analysis (7-20). In specific, it has been demonstrated that high tumor grade is an adverse prognostic factor. In the vast majority of studies documenting the prognostic power of tumor grade, the number of grades has been collapsed, and a two-tiered stratification schema (i.e., high grade and low grade) has been employed for data analysis. In the two-tiered systems, low grade has included both well differentiated and moderately differentiatedadenocarcinomas, and high grade has included poorly differentiated and undifferentiated cancers. In general practice, a two-tiered grading systems would also be expected to reduce interobserver variability, since the widest variations in grading occur with stratification of low grade tumors into well- or moderately-differentiated categories and diagnosis of poorly differentiated or undifferentiated tumors is more consistent (21). In light of its proven prognostic value as well as its relative simplicity and reproducibility, the use of a two-tiered grading system for colorectal carcinoma would be advisable but, unfortunately, is not common practice.

It has also been demonstrated that RER+ colorectal cancers tend to be less well differentiated than RER- tumors. Thus, it may be possible to recognize RER+ on the basis of their degree of differentiation.

In this study, all tumors will be graded according to a two-tiered system as either low grade or high grade. Correlation with outcome will be established by multivariate analysis to determine the independent prognostic significance of tumor grade in stage II colorectal cancer, high-risk stage II colorectal cancer and stage III disease, respectively. In addition, correlation with other parameters such as RER status will be assessed.

2.2 Tumor Proliferation Index

Cell proliferation is considered an indicator of colorectal tumor progression (22,23). The proliferative index of colorectal tumors has been studied almost exclusively by immunolocalization of cell cycle-related antigens such as Ki-67 and proliferating cell nuclear antigen (PCNA) (24-28) and by flow cytometry (29-33). The results of studies correlating proliferative indices determined by either method with clinical outcome or with tumor stage or grade have been contradictory. The immunolocalization studies for Ki-67 have shown no correlation with survival (26,27), but it subsequently has been demonstrated that Ki-67 is also expressed in noncycling cells (34), a fact that

is likely to skew results significantly. By contrast, studies using immunolocalization for PCNA have shown that proliferation indices are independent predictors of tumor recurrence and poor survival (24,25). Correlation of outcome with proliferation indices determined by flow cytometry have also been contradictory, ranging from strong correlation with adverse outcome (33) to no correlation at all with outcome (30).

The practice of assessing tumor proliferation by mitotic figure counts (i.e., number of mitotic figures per 10 high power fields) is commonplace with regard to sarcomas. In fact, it is the standard method by which tumor proliferation is determined and by which histologic grade assigned in most sarcomas. By contrast, mitotic figure counting is rarely, if ever performed for carcinomas. Historically, mitotic density determination fell out of favor as a method of choice for estimating proliferation indices for colorectal cancer after the 1930s. However, it has been the subject of recent studies (35) and found to correlate well with PCNA staining, but it is not widely practiced. Recently, the subject of mitotic indices as a method for estimating proliferation rates in colorectal cancer was revisited by the Colorectal Working Group of the AJCC (1). Although little data on this method currently exists, the consensus of the group was that the method held great promise in that it is inexpensive, reproducible, universally available, easy to quality control, and correlates with data derived by more complex and expensive methods. Furthermore, it can be performed prospectively or retrospectively with equal accuracy. If shown to be prognostically significant, mitotic counting would be justified as a part of the basic pathologic analysis in all cases of colorectal cancer.

In this study, mitotic counts will be performed on all three of the H&E sections produced by the CALGB PCO for each tumor in these studies. The average of the three counts will be calculated as the mitotic index and the mitotic index will be correlated with outcome.

3.0 PATHOLOGIC DATA COLLECTED BY THE SUBMITTING PATHOLOGIST ACCORDING TO CALGB GUIDELINES

3.1 Tumor Border Configuration

For colorectal cancer, the growth pattern of the tumor at the advancing edge (tumor border) has been shown to have prognostic significance that is independent of stage and may predict liver metastasis. Specifically, an irregular, infiltrating pattern of growth, as opposed to a pushing border, has been demonstrated to be an adverse prognostic factor by several univariate (21,36-38) and multivariate analyses (39-45). In some of these studies, infiltrating tumor borders have been referred to as "focal dedifferentiation" (38) and "tumor budding" (44) and defined as microscopic clusters of undifferentiated cancer cells just ahead of the invasive front of the tumor. In a study by Jass et al. (40), interobserver variability among pathologists evaluating tumor border configuration was found to be about 30% if no specific definitions of infiltrating growth were provided. Concordance was found to improve to 90% when the following diagnostic criteria were employed (40): 1) inability to define limits of invasive border of tumor and/or to resolve host tissue from malignant tissue on naked eye examination of the slide; and 2) “streaming” of tumor through the full thickness of the muscularis propria without stromal response and/or dissection of mesenteric adipose tissue by small glands or irregular clusters or cords of cells and/or perineural invasion on microscopic examination.

In this study tumor border configuration will be analyzed according to the above criteria by the submitting pathologist. The guidelines and rational for tumor border evaluation are detailed in the CALGB Pathology Practice Protocol for Colorectal Cancer which is provided to all participatinginstitutions and is included in Appendix III. The tumor border will be assessed as either infiltrating or pushing. Tumor border

configuration will be correlated with outcome to determine the independent prognostic significance of this feature.

3.2 Peritumoral Host Lymphoid Response

Lymphocytic infiltration of tumor or peritumoral tissue is indicative of a host immunologic response and has been shown by multivariate analysis in several studies to be a favorable prognostic factor (13,21,40,43). However, other studies have either failed to confirm the prognostic significance of a peritumoral lymphoid reaction (39,45) or have demonstrated its significance only by univariate analysis (36,46-48). The specific features that are considered indicative of a host immunologic reaction to tumor include perivascular lymphocytic cuffing in the muscularis propria, perivascular lymphocytic cuffing in the pericolonic fat or subserosa, lymphocytic infiltration at the tumor edge, and a "Crohn's-like" lymphoid reaction (i.e., transmural peritumoral lymphoid follicle formation).

In this study the peritumoral host lymphoid response will be analyzed by the submitting pathologist. The guidelines and rational for evaluation of the host lymphoid response are detailed in the CALGB Pathology Practice Protocol for Colorectal Cancer which is provided to all participating institutions and is included in Appendix III. Peritumoral host lymphoid response will be assessed as either present or absent and will be correlated with outcome to determine the independent prognostic significance of the presence (or absence) of this feature.

3.3 Lymphatic Vessel, Venous Vessel and Perineural Invasion

Venous invasion by tumor has been demonstrated to have an independent adverse impact on outcome by multivariate analysis in at least 10 different studies (7,10-12,20,21,49-51,53) and by univariate analysis in several additional studies (36,53-55). However, some studies identifying venous invasion as an adverse prognostic factor on univariate analysis have failed to confirm an independent effect on multivariate analysis (56,57). Similarly disparate results have been reported for lymphatic invasion as well (14,37,49,50,52,54,56-58). Further complicating the issue, several multivariate analyses demonstrating vascular invasion to be a prognostically significant factor have made no distinction between lymphatic and venous vessels. In yet other studies, the location of the vascular involvement (e.g., invasion of extramural veins) has been a strong determinate of the associated prognostic significance (5,21). Perineural invasion has been shown to be an independent indicator of poor prognosis in some studies (7,10,14,50,52,59,60) but not in others. Overall, the prognostic importance of these features is strongly suggested by the literature but remains to be confirmed by definitive prospective analysis.

In this study, lymphatic, venous, and perineural invasion will be analyzed by the submitting pathologist. The guidelines and rational for evaluation of these features are detailed in the CALGB Pathology Practice Protocol for Colorectal Cancer which is provided to all participating institutions and is included in Appendix III. Each of these features will be assessed as either present or absent and will be correlated with outcome to determine their independent prognostic significance.

4.0 STATISTICS

Power estimates are presented for the primary endpoint of overall survival. The following pathologic features will be studied: tumor grade; tumor mitotic index; tumor border configuration; host lymphoid response to tumor; lymphatic vessel, venous vessel and perineural invasion.

Each pathologic feature will be analyzed separately for an association with survival. The expected median survival in this population of patients with Stage III colon cancer is 8 years. Power calculations are presented in Tables 1 and 2 below to detect differences in

median survival between 6.5 years and 9.0 years (hazard ratio 1.4) and 7.5 years and 10.5 years (hazard ratio 1.4) at three prevalence levels for the pathologic variable: 10%, 30% and 50%. Similarly, Tables 3 and 4 contain power estimates to detect differences in median survival between 6.5 years and 10.5 years (hazard ratio 1.6) and 7.5 years and 12 years (hazard ratio 1.6). All power estimates are based on the following assumptions: 1) accrual of 450 patients (or 337 patients at 75% of total enrollment) per year for 2.8 years; 2) three years follow-up; 3) two-sided log rank test; 4) exponential survival; 5) improved survival in the “smaller” arm for unequal sample sizes. Power estimates are provided for significance levels of 0.01 and 0.05. The Cox Regression Model will also be used to study the simultaneous impact of these variables on survival.

Table 1. Power estimates to detect the difference in median survival between 6.5 and 9.0 years (hazard ratio 1.4) for prevalence of 10, 30 and 50% and significance levels 0.01 and 0.05. Power for 75% total enrollment given in parentheses.

**Prevalence**	α	**Power**
10%	0.01	0.24 (0.17)
	0.05	0.47 (0.37)
30%	0.01	0.64 (0.49)
	0.05	0.84 (0.72)
50%	0.01	0.76 (0.61)
	0.05	0.91 (0.81)

REFERENCES

1. Compton CC, Fenoglio-Preiser CM, Pettigrew N, Fielding LP. American Joint Committee on Cancer. Prognostic factors consensus conference: Colorectal Working Group. Cancer (manuscript submitted to Cancer).

2. Fleming ID, Cooper JS, Henson DE, et al. eds., AJCC Manual for Staging of Cancer, 5th ed, Lippincott Raven, Philadelphia, 1997.

3. Compton CC, Henson DE, Hutter RVP, et al. Protocol for the Examination of Specimens Removed from Patients with Colorectal Carcinoma. Arch Pathol Lab Med 121;1247-54, 1998.

4. Compton CC, Sobin LH. Carcinoma of the Stomach. In: Compton CC, editor. Practice Protocol Manual for the Examination of Specimens Removed from Patients with Cancer. 1st Edition. Northfield (IL): College of American Pathologists, 1998.

5. Blenkinsopp W, Stewart-Brown S, Blesovsky L, et al. Histopathology reporting in large bowel cancer. J Clin Pathol 34:509-513, 1981.

6. Thomas GHD, Dixon MF, Smeeton NC, et al. Observer variation in the histological grading of rectal carcinoma. J Clin Pathol 36:385-391, 1983.

7. Chapuis PH, Dent OF, Fisher R, et al. A multivariate analysis of clinical and pathological variables in prognosis after resection of large bowel cancer. Br J Surg 72:698-702, 1985.

8. D'Eredita G, Serio G, Neri V, et al. A survival regression analysis of prognostic factors in colorectal cancer. Aust NZ J Surg 66:445-451, 1996.

9. Griffin M, Bergstralh E, Coffey R, et al. Predictors of survival after curative resection of carcinoma of the colon and rectum. Cancer 60:2318-2324, 1987.

10. Mulcahy HE, Skelly MM, Husain A, et al. Long-term outcome following curative surgery for malignant large bowel obstruction. Br J Surg 83:46-50, 1996.

11. Newland R, Dent O, Lyttle M, et al. Pathologic determinants of survival associated with colorectal cancer with lymph node metastases. A multivariate analysis of 579 patients. Cancer 73:2076-2082, 1994.

12. Freedman L, Macaskill P, Smith A. Multivariate analysis of prognostic factors for operable rectal cancer. Lancet II:733-736, 1984

13. Deans G, Heatley M, Anderson N, et al. Jass Classification Revisited. J. Am. Coll. Surg. 179:11-17, 1994.

14. Hermanek P, Guggenmoos-Holzmann I, Gall FP. Prognostic factors in rectal carcinoma. A contribution to the further development of tumor classification. Dis Colon Rectum 32:593-599, 1989.

15. Robey-Cafferty SS, el-Naggar AK, Grignon DJ, et al. Histologic parameters and DNA ploidy as predictors of survival in stage B adenocarcinoma of colon and rectum. Mod Pathol 3:261-266, 1990.

16. Böttger TC, Potratz D, Stöckle M, et al. Prognostic value of DNA analysis in colorectal carcinoma. Cancer 72:3579-3587, 1993.

17. Fisher E, Sass R, Palekar A, et al. Dukes' classification revisited. Findings from the National Surgical Adjuvant Breast and Bowel Projects. Cancer 64:2354-2360, 1989.

18. Jessup J, McGinnis L, Steele G, et al. The National Cancer Data Base Report on Colon Cancer. Cancer 78:918-926, 1996.

19. Scott NA, Wieand HS, Moertel CG, et al. Colorectal cancer. Dukes' stage, tumor site, preoperative plasma CEA level, and patient prognosis related to tumor DNA ploidy pattern. Arch Surg 122:1375-1379, 1987.

20. Wiggers T, Arends J, Volovics A. Regression analysis of prognostic factors in colorectal cancer after curative resections. Dis Colon Rectum 31:33-, 1988.

21. Harrison J, Dean P, El-Zeky F, et al. From Dukes through Jass: Pathological prognostic indicators in rectal cancer. Hum Pathol 25:498-505, 1994.

22. Risio M. Cell proliferation in colorectal tumor progression: an immunohistochemical approach to intermediate biomarkers. J Cell Biol 16G:79-87, 1992.

23. Moss SF, Liu TC, Petrotos A, et al. Inward growth of colonic adenomatous polyps. Gastroenterology 111:1425-1432, 1996.

24. Mayer A, Takimoto M, Fritz E, et al. The prognostic significance of proliferating cell nuclear antigen, epidermal growth factor receptor, and *mdr* gene expression in colorectal cancer. Cancer 71:2454-2460, 1993.

25. Al-Sheneber IF, Shibata HR, Sampalis J, Jothy S. Prognostic significance of proliferating cell nuclear antigen expression in colorectal cancer. Cancer 71:1954-1959, 1993.

26. Kubota Y, Petras RE, Easley KA, et al. Ki-67-determined growth fraction versus standard staging and grading parameters in colorectal carcinoma. Cancer 70:2602-2609, 1992.

27. Sahin AA, Ro JY, Brown RW, et al. Assessment of Ki-67-derived tumor proliferation activity in colorectal adenocarcinomas. Mod Pathol 7:17-22, 1994.

28. Neoptolemos JP, Oates KM, Newbold KM, et al. Cyclin/proliferation cell nuclear antigen immunohistochemistry does not improve the power of Dukes' or Jass' classifications for colorectal cancer. Br J Surg 82:184-187, 1995.

29. Rew DA, Wilson GD, Taylor I, Weaver PC. Proliferation characteristics of human colorectal carcinomas measured in vitro. Br J Surg 78:60-66, 1991.

30. Bleiberg H, Buyes M, Van den Heule B, Galand P. Cell cycle parameters and prognosis of colorectal cancer. Eur J Cancer Clin Oncol 20:391-396, 1984.

31. Enker WE, Kimmel M, Cibas ES, et al. DNA/RNA content and proliferative fractions of colorectal carcinomas: A five-year prospective study relating flow cytometry to survival. JNCI 83:701-707, 1991.

32. Wahlstrom B, Branehog I, Stierner U, et al. Association of ploidy and cell proliferation, Dukes' classification, and histopathological differentiation in adenocarcinomas of colon and rectum. Eur J Surg 158:237-243, 1992.

33. Witzig TE, Loprinzi CL, Gonchoroff NJ, et al. DNA ploidy and cell measurements as predictors of recurrence and survival in stages B2 and C adenocarcinoma. Cancer 68:879-888, 1991.

34. Van Oijen MGCT, Medema RH, Slootweg PJ, Rijken G. Positivity of the proliferation marker Ki-67 in noncycling cells. Am J Clin Pathol 110:24-31, 1998.

35. Willett CG, Warland G, Cheek R, et al. Proliferating cell nuclear antigen (PCNA) and mitotic activity in rectal cancer: predictor of response to preoperative irradiation. J Clin Oncol 12:679-82, 1994.

36. Carlon C, Fabris G, Arslan-Pagnini C, et al. Prognostic correlations of operable carcinoma of the rectum. Dis Colon Rectum 28:47-50, 1985.

37. Minsky B, Mies C, Rich T, Recht A. Lymphatic vessel invasion in an independent prognostic factor for survival in colorectal cancer. Int J Radiation Oncology Biol Phys 17:311-318, 1989.

38. Ono M, Sakamoto M, Ino Y, et al. Cancer cell morphology at the invasive front and expression of cell adhesion-related carbohydrate in the primary lesion of patients with colorectal carcinoma with liver metastasis. Cancer 78:1179-1186, 1996.

39. Roncucci L, Fante R, Losi L, et al. Survival for colon and rectal cancer in a population-based cancer registry. Eur J Cancer 32A:295-302, 1996.

40. Jass JR, Atkin W, Cuzick J, et al. The grading of rectal cancer: historical perspectives and a multivariate analysis of 447 cases. Histopathology 10:437-459, 1986.

41. Jass JR, Love S, Northover J. A new prognostic classificationof rectal cancer. Lancet I:1303-1306, 1987.

42. Shepherd N, Saraga E, Love S, Jass JR. Prognostic factors in colonic cancer. Histopathology 14:613-620, 1989.

43. Desmoplasia and survival in colorectal cancer. J Clin Pathol 42:162-166, 1989.

44. Hase K, Shatney C, Johnson D, et al. Prognostic value of tumor “budding” in patients with colorectal cancer. Dis Colon Rectum 36:627-635, 1993.

45. Thynne GS, Weiland LH, Moertel CG, Silvers A. Correlation of histopathologic characteristics of primary tumor and uninvolved regional lymph nodes in Dukes’ C colonic carcinoma with prognosis. Mayo Clin Proc 55:243-245, 1980.

46. Pihl E, Malahy MA, Khankhanian N, et al. Immunomorphological features of prognostic significance in Dukes’ class B colorectal carcinoma. Cancer Res. 37:4145-4149, 1977.

47. Svennevig JL, Lunde OC, Holter J, Bjørgsvik D. Lymphoid infiltration and prognosis in colorectal carcinoma. Br J Cancer 49:375-377, 1984.

48. Zhou XG, Yu BM, Shen YX. Surgical treatment and late results in 1226 cases of colorectal cancer. Dis Colon Rectum 26:250-256, 1983.

49. Michelassi F, Ayala J, Balestracci T, et al. Verification of a new clinicopathologic staging system for colorectal adenocarcinoma. Ann Surg 214:11-18, 1991.

50. Michelassi F, Block GE, Vannucci L, Montag A, Chappell R. A 5- to 21-year follow-up and analysis of 250 patients with rectal adenocarcinoma. Ann. Surg. 208:379-387, 1988.

51. Heys S, Sherif A, Bagley J, et al. Prognostic factors and survival of patients aged less than 45 years with colorectal cancer. Br J Surg 81:685-688, 1994.

52. Knudsen JB, Nilsson T, Sprechler M, et al. Venous and nerve invasion as prognostic factors in postoperativesurvival of patients with resectable cancer of the rectum. Dis Colon Rectum 26:613-617, 1983.

53. Horn A, Dahl O, Morild I. Venous and neural invasion as predictors of recurrence in rectal adenocarcinoma. Dis Colon Rectum 34:798-804, 1991.

54. Lee Y. Local and regional recurrence of carcinoma of the colon and rectum: I. tumour-host factors and adjuvant therapy. Surg Oncol 4:283-293, 1995.

55. Talbot I, Ritchie S, Leighton MH, et al. The clinical significance of invasion of veins by rectal cancer. Br J Surg 67:439-442, 1980.

56. Takebayashi Y, Akiyama S, Yamada K, et al. Angiogenesis as an unfavorable prognostic factor in human colorectal carcinoma. Cancer 78:226-31, 1996.
