# Supplementary material for: MRE11-Deficiency Associated with Improved Long-Term Disease Free Survival and Overall Survival in a Subset of Stage III Colon Cancer Patients in Randomized CALGB 89803 Trial
Source: PLoS One. 2014 Oct 13;9(10):e108483. doi: 10.1371/journal.pone.0108483 (PMC4195600; doi:10.1371/journal.pone.0108483)
Supplement: Institutions S1 — CALBG 89803 Institutions. Institutions and investigators that participated in the initial CALGB 89803 study. (PDF) [file pone.0108483.s003.pdf]

Institutions and investigators that participated in the initial CALGB 89803 study:

- Baptist Cancer Institute CCOP, Memphis, TN - Lee S. Schwartzberg, M.D., supported by CA71323
- Christiana Care Health Services, Inc. CCOP, Wilmington, DE - Stephen Grubbs, M.D., supported by CA45418
- Dana-Farber Cancer Institute, Boston, MA - Eric P. Winer, M.D., supported by CA32291
- Dartmouth Medical School - Norris Cotton Cancer Center, Lebanon, NH - Marc S. Ernstoff, M.D., supported by CA04326
- Duke University Medical Center, Durham, NC - Jeffrey Crawford, M.D., supported by CA47577
- Georgetown University Medical Center, Washington, DC - Minetta C. Liu, M.D., supported by CA77597
- Cancer Centers of the Carolinas, Greenville, SC - Jeffrey K. Giguere, M.D., supported by CA29165
- Hematology-Oncology Associates of Central New York CCOP, Syracuse, NY - Jeffrey Kirshner, M.D., supported by CA45389
- Long Island Jewish Medical Center, Lake Success, NY - Kanti R. Rai, M.D., supported by CA11028
- Massachusetts General Hospital, Boston, MA - Jeffrey W. Clark, M.D., supported by CA12449
- Memorial Sloan-Kettering Cancer Center, New York, NY - Clifford A. Hudis, M.D., supported by CA77651
- Missouri Baptist Medical Center, St. Louis, MO - Alan P. Lyss, M.D., supported by CA114558-02
- Mount Sinai Medical Center, Miami, FL - Rogerio C. Lilenbaum, M.D., supported by CA45564
- Mount Sinai School of Medicine, New York, NY - Lewis R. Silverman, M.D., supported by CA04457
- Nevada Cancer Research Foundation CCOP, Las Vegas, NV - John A. Ellerton, M.D., supported by CA35421
- North Shore-Long Island Jewish Health System, New Hyde Park, NY - Daniel Budman, M.D., supported by CA35279
- Rhode Island Hospital, Providence, RI - William Sikov, M.D., supported by CA08025
- Roswell Park Cancer Institute, Buffalo, NY - Ellis Levine, M.D., supported by CA02599
- Southeast Cancer Control Consortium Inc. CCOP, Goldsboro, NC - James N. Atkins, M.D., supported by CA45808
- State University of New York Upstate Medical University, Syracuse, NY - Stephen L. Graziano, M.D., supported by CA21060
- The Ohio State University Medical Center, Columbus, OH - Clara D. Bloomfield, M.D., supported by CA77658
- University of California at San Diego, San Diego, CA - Barbara A. Parker, M.D., supported by CA11789
- University of California at San Francisco, San Francisco, CA - Alan P. Venook, M.D., supported by CA60138
- University of Chicago, Chicago, IL - Gini Fleming, M.D., supported by CA41287
- University of Illinois MBCCOP, Chicago, IL - Lawrence E. Feldman, M.D., supported by CA74811

- University of Iowa, Iowa City, IA - Daniel A. Vaena, M.D., supported by CA47642
- University of Maryland Greenebaum Cancer Center, Baltimore, MD - Martin Edelman, M.D., supported by CA31983
- University of Massachusetts Medical School, Worcester, MA - William V. Walsh, M.D., supported by CA37135
- University of Minnesota, Minneapolis, MN - Bruce A Peterson, M.D., supported by CA16450
- University of Missouri/Ellis Fischel Cancer Center, Columbia, MO - Michael C. Perry, M.D., supported by CA12046
- University of Nebraska Medical Center, Omaha, NE - Anne Kessinger, M.D., supported by CA77298
- University of North Carolina at Chapel Hill, Chapel Hill, NC - Thomas C. Shea, M.D., supported by CA47559
- University of Tennessee Memphis, Memphis, TN - Harvey B. Niell, M.D., supported by CA47555
- University of Vermont, Burlington, VT - Hyman B. Muss, M.D., supported by CA77406
- Wake Forest University School of Medicine, Winston-Salem, NC - David D Hurd, M.D., supported by CA03927
- Walter Reed Army Medical Center, Washington, DC - Thomas Reid, M.D., supported by CA26806
- Washington University School of Medicine, St. Louis, MO-Nancy Bartlett, M.D., supported by CA77440
- Weill Medical College of Cornell University, New York, NY - John Leonard, M.D., supported by CA07968
